# Halogenase-Assisted Biocatalytic Derivatization of Aminothiazoles and Cephalosporin Antibiotics

**DOI:** 10.1021/acs.joc.4c03043

**Published:** 2025-02-20

**Authors:** Paul J. Branham, Nirmal Saha, Sophia E. Oyelere, Vinayak Agarwal

**Affiliations:** † School of Chemistry and Biochemistry, Georgia Institute of Technology, Atlanta, Georgia 30332, United States; ‡ School of Biological Sciences, Georgia Institute of Technology, Atlanta, Georgia 30332, United States

## Abstract

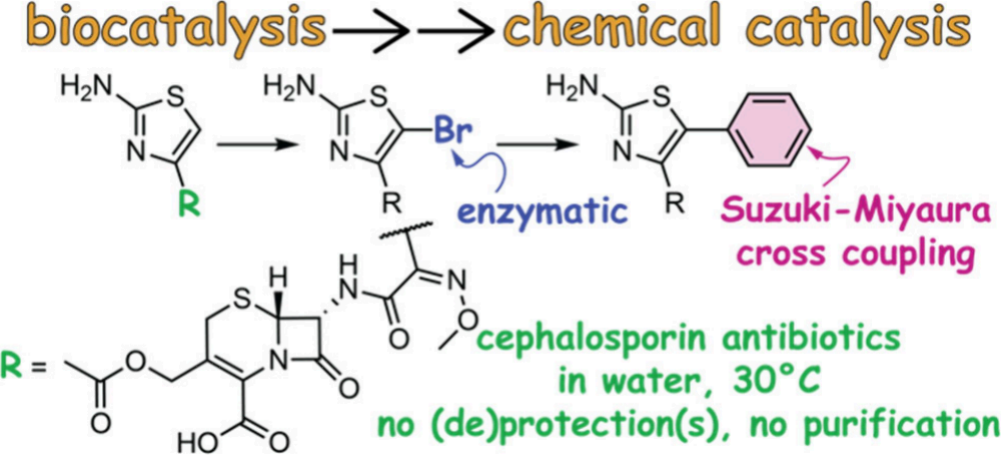

With a view toward the prominence of brominated intermediates in chemical synthesis, we describe here a biocatalytic scheme for the enzymatic bromination of 2-aminothiazoles using a marine macroalgal brominase in reaction conditions that are directly compatible with Suzuki–Miyaura cross-coupling reactions. Enzymatically delivered brominated thiazoles, without intermediary purification, are arylated in high yields. We demonstrate the applicability of the methodology described herein in derivatizing clinically administered cephalosporin antibiotics and prodrugs in an aqueous solvent and mild reaction conditions.

The 2-aminothiazole motif is widely present in pharmaceuticals, including the third, fourth, and fifth generations of cephalosporin antibiotics (shaded, Figure 1).^1^ Thus, derivatization of the 2-aminothiazole moiety under mild conditions that does not require manipulation and/or protection of other reactive handles on a pharmacophore is desirable. Among C–H activation strategies, halogenation provides a straightforward route to downstream cross-coupling reactions.^2^ In this study, we explored biocatalytic routes for 2-aminothiazole halogenation and subsequent transition metal-assisted C–C bond forming reactions for 2-aminothiazoles and 2-aminothiazole-containing cephalosporin antibiotics under mild aqueous conditions.

**Figure 1:**
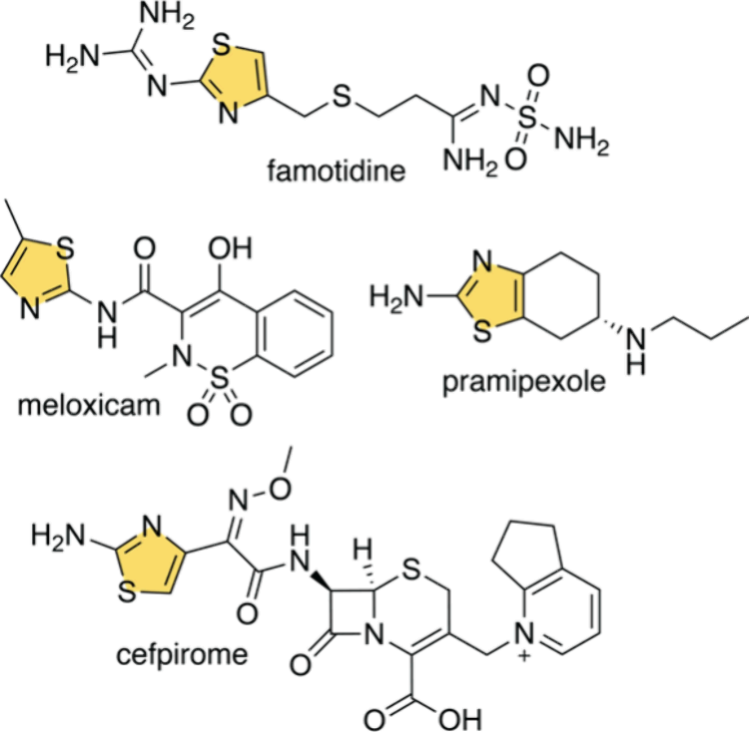
2-Aminothiazole moiety in antacid famotidine, analgesic meloxicam, dopamine agonist used in Parkinson’s disease pramipexole, and fourth-generation cephalosporin antibiotic cefpirome.

Biocatalytic halogenation is rooted in natural product biosynthetic chemistry. Among the different classes of halogenases, the vanadium-dependent haloperoxidases (VHPOs) offer an expansive substrate scope.^3^ These enzymes catalyze halide to hypohalous acid oxidation, leading to halogenation at electron rich sites in hydrocarbon substrates. Chloride, bromide, and iodide oxidation by VHPOs has been reported. En route halide oxidation, electrons are funneled to hydrogen peroxide (H_2_O_2_) via the acyl-vanadate cofactor (Figure S1). Marine phototrophs and actinobacteria are prolific sources of VHPOs with algae-derived VHPOs often offering the unique advantage of catalyzing bromination without contaminating chlorination even in chloride-rich reaction conditions.^3^ With a view toward using halogens as a handle for further derivatization, substrate bromination is desirable due to mild reaction conditions for cross coupling reactions as compared to chlorination.^4^

Building upon foundational literature,^5−8^ we had reported the discovery of brominating VHPOs from marine algae wherein these enzymes participate in the production of bromoform (CHBr_3_).^9,10^ To gauge the relative activity of the brominating VHPOs from model Rhodophytes, four open reading frames encoding VHPOs from seaweeds *Asparagopsis taxiformis* (At) and *Chondrus crispus* (*Cc*) were expressed in *Escherichia coli* and recombinant enzymes were purified (Table S1). Of these, one of the CcVHPOs, henceforth referred to as CcVHPO1, demonstrated maximal bromoform production (Figure S2). Negative control reactions omitting the enzyme, H_2_O_2_, vanadate, or bromide abolished bromoform production (Figure S3). No chlorinated or mixed halogenated products were detected to be produced, establishing CcVHPO1 as an obligate brominase.

Next, we tested the bromination activity of CcVHPO1 for 2-aminothiazole (**1**). Noteworthy here—as compared to methods for bromination in chemical synthesis—is the use of a nontoxic inorganic bromide salt as the source of the bromine atom, aqueous solvent, reaction proceeding at mild temperature (30 °C), low catalyst loading, and no organic byproduct formation (Figure 2A, Table S2, Figures S4–S6). The only caveat was the use of H_2_O_2_ as the oxidant. Hence, we evaluated the substrate conversion against stoichiometric amounts of H_2_O_2_ in the reaction. Maximal conversion was observed at 2 equiv of H_2_O_2_ in the reaction with a 1 h reaction time (Figure S7); all reactions henceforth were conducted with 2 equiv of H_2_O_2_. Product identity as 2-amino-5-bromothiazole was confirmed by comparison with an authentic synthetic standard conforming to an electrophilic aromatic substitution (Figures S8–S11).

**Figure 2:**
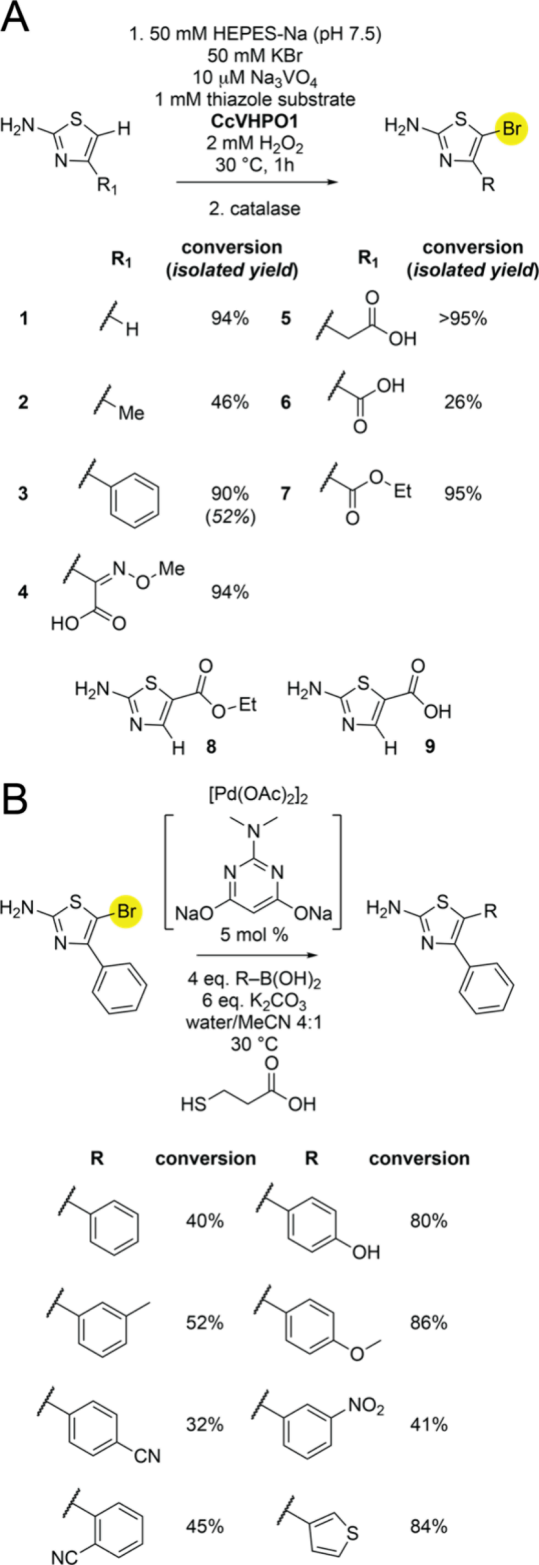
(A) Enzymatic bromination of 2-aminothiazoles **1**–**9**. Note that brominated product formation was not observed for molecules **8** and **9** that are isomers of **7** and **6**, respectively. Reactions in which no leftover starting material was detected are reported to proceed with >95% conversion. Bromination reactions were quenched by the addition of catalase. (B) Reaction conditions for SMCC reactions of 5-bromo-4-phenyl-2-aminothiazole with a panel of boronic acids.

We then evaluated the substrate scope for the biocatalytic bromination reaction. Product formation was observed in excellent conversions for a majority of 4-acyl-2-aminothiazoles (Figure 2A, Table S2, and Figures S12–S29). The brominated product starting from **3**, **3-Br**, was prepared at a preparative scale and characterized by NMR in 52% isolated yield (Figures 2A, S30–S31). Consistent with the electrophilic aromatic substitution reaction mechanism, 2-aminothiazoles acylated at the 5-position—molecules **8** and **9**—were not brominated (Figures S32–S37). Of the substrates tested here, efficient bromination of **4** is particularly noteworthy as **4** is a precursor for the synthesis of cephalosporin antibiotics via condensation with 7-aminocephalosporanic acid and derivatives thereof.^11^ Mass spectral fragmentation established that monobromination was neatly affected upon the aminothiazole 5-position of **4** with the oxime functionality—which is preserved in numerous clinically used semisynthetic cephalosporins—not degraded under the reaction conditions employed here (Figure S38).

With a route for biocatalytic bromination of aminothiazoles in hand, we explored if Pd-assisted Suzuki–Miyaura cross-coupling (SMCC) reactions could be affected by brominated products obtained above. The brominated products were not purified; the only processing that the bromination reactions underwent was the quenching of any residual H_2_O_2_ by the addition of catalase. Without any intermediary purification, excellent conversions for coupling of various boronic acids were obtained starting from **3** with the *p*-methoxyphenyl coupling product characterized by NMR (Figure 2B, Table S3, and Figures S39–S47). These data establish the utility of developing a biocatalytic bromination scheme with reaction conditions that are directly compatible with downstream reactions that are derived from traditional chemical synthesis schemes. Critical here is the observation that no organic byproduct is generated in the bromination reaction that would be incompatible with the SMCC reaction. Quenching the SMCC reactions with mercaptopropionic acid facilitated downstream mass spectrometric experiments.^12^

As mentioned above, molecule **4** serves as a precursor for industrial synthesis of cephalosporin antibiotics.^13^ Progressing from the enzymatically generated brominated-**4**, SMCC reactions enabled arene additions to be affected upon **4**. Mass spectrometric fragmentation demonstrated that arene additions were neatly installed upon the thiazole ring with the oxime functionality unaffected (Figure 3, Figures S48–S49).

**Figure 3:**
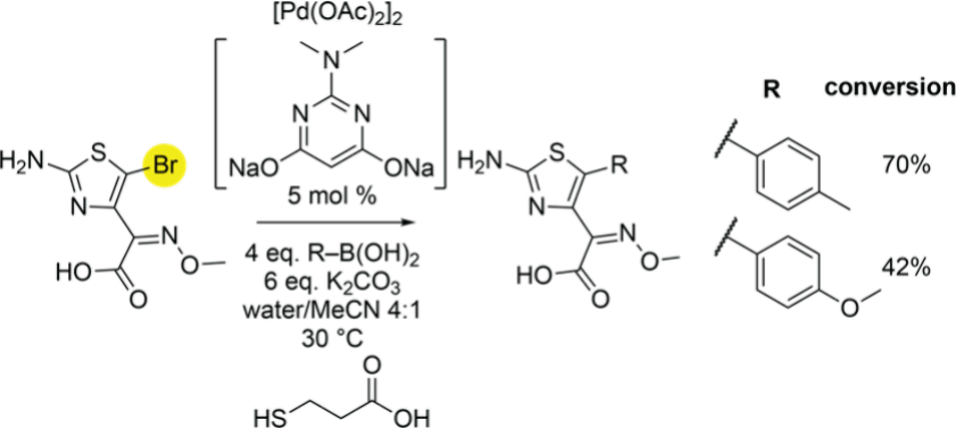
SMCC reactions for arene-addition products generated starting from brominated-**4**.

With methods for enzymatic bromination and SMCC for model substrates established, we turned our attention to clinically used cephalosporin antibiotics that also possess a 2-aminothiazole moiety. Here, we tested enzymatic bromination and SMCC reactions for the third-generation cephalosporin cefotaxime (**10**), fourth-generation cephalosporin cefepime (**11**), and cefcapene pivoxil (**12**), the prodrug form of the cephalosporin cefcapene. While **10** and **11** are delivered via injection, **12** can be administered orally; it undergoes acid-mediated pivoxil ester hydrolysis in the gut to release the active antibiotic.^14^

Treatment with **10**–**12** with CcVHPO1 afforded the brominated products with high conversions as deduced by mass spectrometry (Figure 4A, Table S1). Preparative-scale bromination of **12** and characterization of the isolated brominated product **12-Br** by NMR demonstrated substitution of an aromatic proton thusly localizing the site of bromination to the aminothiazole moiety (Figure 4B, Figures S50–S54). This then allowed for structural annotation of the MS^2^ fragment ions observed for **12-Br** (Figure 4C). Using the characteristic fragmentation patterns thus discerned, bromination upon **10** and **11** was similarly rationalized to occur on the aminothiazole ring (Figure 4D–4E, Figures S55–S66). The bromination reactions were quenched by the addition of catalase, and with no intermediary purification, **10-Br** afforded SMCC products with high conversions (Figure 4A, Table S3, and Figures S67–S68). Mass spectrometric fragmentation demonstrated that the arene addition was afforded upon the 2-aminothiazole moiety (Figure 4F–4G). The SMCC reactions were quenched by the addition of mercaptopropionic acid; acid treatment led to pivoxil ester hydrolysis for **12** affording the deprotected arene addition products (Figure 4A, Table S3, and Figures S69–S70). As before, mass spectrometric fragmentation localized arene addition to the 2-aminothiazole moiety of **12** (Figures 4H–4I). These observations validate the utility of the chemoenzymatic process described here in delivering cephalosporin derivatives in a single-pot reaction under mild reaction conditions. The efficacy of the cephalosporin derivatives generated in this study is underway.

**Figure 4:**
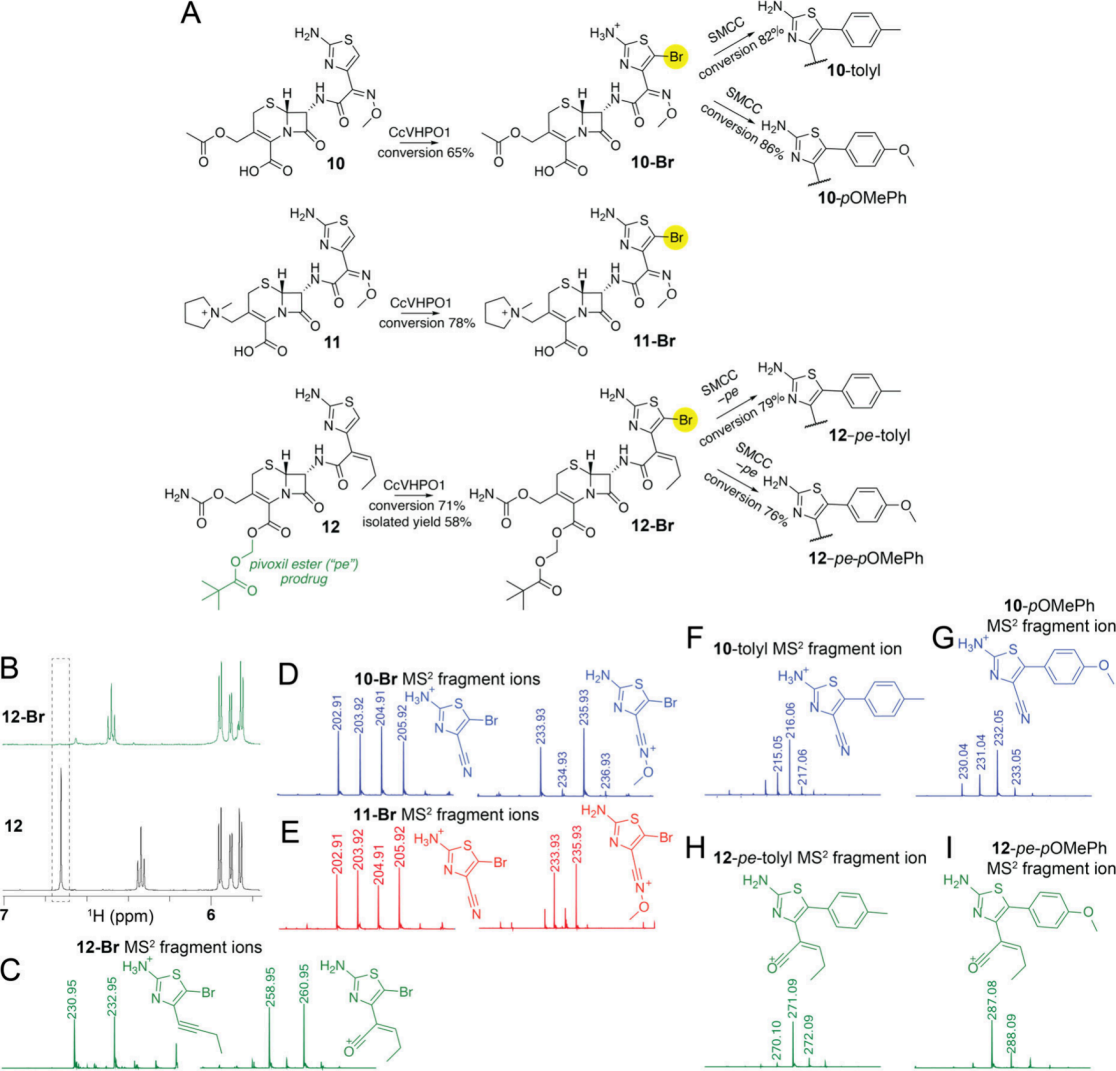
(A) Enzymatic bromination and SMCC reactions for **10**–**12**. Note that the pivoxil ester moiety in **12**, abbreviated as *pe*, was hydrolyzed when the SMCC reactions were quenched by mercaptopropionic acid. (B) Abbreviated ^1^H NMR spectra for **12-Br** (top, in green) and **12** (bottom, in black) with a dashed box demonstrating a singlet in the bottom spectra that is absent in the top spectra implying substitution of an aromatic proton in **12** upon bromination. Also note that the alkene proton is deshielded upon bromination as is implied by the downfield shift of the triplet. (C–I) Abbreviated MS^2^ fragmentation spectra demonstrating structurally annotated characteristic product ions that allow for the bromine atom and the SMCC arene additions to be localized to the 2-aminothiazole moieties. For the brominated product ions, the isotopic signature characteristic of brominated species is evident. The unabbreviated MS^2^ spectra are available in the Supporting Information.

Halogenases are increasingly being recognized as valuable catalysts for late stage halogenation of structurally and chemically elaborate substrates.^15−19^ As is the hallmark of enzyme catalysis, among the VHPOs, halogenation can be exquisitely regiospecific and substrate selective.^20^ However, substrate selectivity among VHPOs is attributed to marine actinobacterial VHPOs while the marine macroalgal VHPOs tend to be broadly substrate promiscuous.^21,22^ In this study itself, a single enzyme—CcVHPO1—brominates substrates that are widely divergent in their size and architecture. As such, a substrate binding site for VHPOs has not been identified, and substrate engagement in the enzyme active site seems to be uncoupled from halide oxidation. Thus, VHPOs are ideal candidates to explore biocatalytic halogenation applications, wherein the inherent reactivity of the substrate guides the enzymatic halogenation outcome.

## Data Availability

The data underlying this study are available in the published article and its Supporting Information.

## Supporting Information Available

Comprehensive description of materials and methods used in this study, synthetic schemes, compound characterization data, and descriptions of enzyme reaction outcomes. (PDF)
