# Supplementary material for: Halogenase-Assisted Biocatalytic Derivatization of Aminothiazoles and Cephalosporin Antibiotics
Source: J Org Chem. 2025 Feb 20;90(9):3507–11. doi: 10.1021/acs.joc.4c03043 (PMC11894640; doi:10.1021/acs.joc.4c03043)
Supplement: Supplementary file 1 — jo4c03043_si_001.pdf [file jo4c03043_si_001.pdf]

SUPPLEMENTARY INFORMATION FOR:

Halogenase-assisted biocatalytic derivatization of aminothiazoles and cephalosporin antibiotics

Paul Branham,[1,‡] Nirmal Saha,[1,‡] Sophia E. Oyelere,[1] Vinayak Agarwal[1,2,*]

[1] School of Chemistry and Biochemistry, Georgia Institute of Technology, Atlanta GA 30332, United States of America

[2] School of Biological Sciences, Georgia Institute of Technology, Atlanta GA 30332, United States of America

‡ Equal contributions authors

* correspondence email:vagarwal@gatech.edu; Ph:404-385-3798

Table of Contents

Supplementary Materials and Methods S2

Supplementary Tables S11

Supplementary Figures S17

Supplementary References S71

SUPPLEMENTARY MATERIALS AND METHODS

General materials

All chemicals, solvents, buffers, and media were obtained through commercial vendors (including Sigma-Aldrich, Fisher Scientific, VWR, and TCI) and used without further purification. The following organic substrates were acquired for use in this study:

- 2-aminothiazole (**1**) from TCI (A0633)
- 2-amino-4-methylthiazole (**2**) from Sigma-Aldrich (A66006)
- 2-amino-4-phenyl-thiazole (**3**) from Fisher Scientific (AAA1848806)
- 2-(2-aminothiazole-4-yl)-2-methoxyiminoacetic (**4**) acid from Sigma-Aldrich (CDS003521)
- 2-amino-4-thiazoleacetic acid (**5**) from Thermo Scientific (B23534.22)
- 2-aminothiazole-4-carboxylic acid (**6**) Sigma-Aldrich (CDS003787)
- ethyl-2-aminothiazole-5-carboxylate (**8**) from Sigma-Aldrich (708437)
- cefotaxime (**10**) from Sigma-Aldrich (C7912)
- cefepime (**11**) from Thermo Scientific (J66237.06)
- cefcapene pivoxil (**12**) from TCI (C2623)

Molecules **7** and **9** were synthesized, as described below.

Enzymatic synthesis of bromoform

The expression and purification of the recombinant VHPOs used in the study—AtMbb1, AtMbb4, CcVHPO1, and CcVHPO2—have been reported previously.[1] The primary sequences and GenBank accession numbers are provided in Table S1. To monitor bromoform production, analytical scale reactions were performed in 500 μL volume in a 1.5 mL microcentrifuge tube containing 50 mM HEPES-Na (pH 7.5), 50 mM KBr, 0.01 mM sodium orthovanadate (Na_3VO_4), 1 mM 2,4-pentanedione, and 1 μM VHPO enzymes. The reaction was initiated by adding 2 μL of 200 mM hydrogen peroxide, and the tube was inverted multiple times for thorough mixing. The reaction was allowed to proceed at 30 °C for 90 min, during which an additional 1 μL of hydrogen peroxide was added every 10 min. At the end of the reaction period, samples were transferred to a 4 mL glass vial. Then, 100 μL of saturated brine and 1 mL of GC/MS-grade diethyl ether were added. The samples were vigorously vortexed and briefly centrifuged. The top organic layer was carefully withdrawn, and the diethyl ether extraction was repeated. The organic layers were pooled, dried *in vacuo*, and reconstituted in 200 μL of diethyl ether for gas chromatography/mass spectrometry (GC/MS) analysis. See Figures S2 and S3 for GC/MS details.

Synthesis of 7

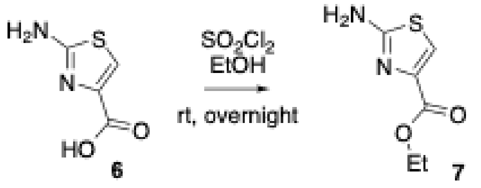

In a reaction vessel, SO_2Cl_2 (165 mg, 1.3 mmol) was added dropwise to a solution of **6** (0.10 g, 0.7 mmol) in 10 mL ice cold ethanol (EtOH). The reaction mixture was stirred overnight at room temperature. The solvent was removed under reduced pressure using a rotary evaporator, and the reaction mixture was washed with saturated sodium bicarbonate solution. The product was extracted using EtOAc (3×10mL), and the solvent was removed using a rotary evaporator. The colorless gum-like product was characterized by ^{1}H-NMR and ^{13}C-NMR comparison to previously reported data.[2]

^{1}H NMR (Methanol-d_4, 500 MHz) δ (ppm) 1.4 (t, J = 7.2 Hz, 3H), 4.3 (q, J = 7.1 Hz, 2H), 7.5 (s, 1H).

^{13}C NMR (Methanol-d_4, 125 MHz): δ (ppm) 13.2, 60.6, 116.7, 142.0, 161.4, 169.6.

Synthesis of 9

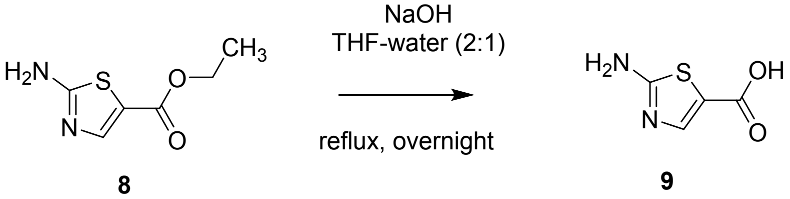

Molecule **9** was synthesized by saponification of **8**; NaOH (0.325 g, 8.15 mmol) was added to a solution of **8** (0.4 g, 2.16 mmol) in a 2:1 mixture of tetrahydrofuran (THF)/water. The reaction mixture was refluxed overnight in a sealed tube. The THF was removed *in vacuo*, and the reaction mixture was neutralized with 1 M HCl, forming an insoluble precipitate. The precipitate was recovered by filtration and washed with diethyl ether. The white solid residue was dried overnight at 60 °C. The product was characterized by ^{1}H-NMR comparison to previously reported data.[3]

^{1}H NMR (DMSO-d_6, 500 MHz) δ (ppm) 7.45 (brs, 2H) 8.48 (s, 1H).

Synthesis of 1-Br

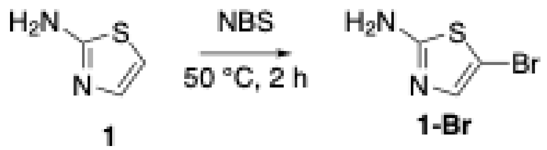

In a 25 mL round bottom flask, **1** (0.102 g, 1.0 mmol) was dissolved in 1 mL glacial acetic acid. Then, N-bromosuccinimide (NBS) (0.231 g, 1.3 mmol) was added and the reaction mixture was stirred for 20 min at 50 °C. The color of the solution turned red, with a corresponding increase in temperature to 65 °C. The reaction mixture was further stirred for 2 h at 50 °C, after which 10 mL water was added, and the solution was transferred to a 50 mL beaker. Diethyl ether (20 mL) was added, and solid Na_2CO_3 was used to adjust the pH of the solution to 8.0. The reaction mixture was extracted twice with diethyl ether, and the combined organic layers were washed three times with water to remove residual succinimide. The orange-red organic layer was evaporated in a rotary evaporator to give a red solid at 50% yield (98 mg). The product was characterized by ^{1}H-NMR and ^{13}C-NMR comparison with previously reported data.[4]

^{1}H NMR ($CDCl_3$, 500 MHz): δ 6.98 (s, 1H), 5.06 (brs, 2H) (Figure S8).

^{13}C NMR ($CDCl_3$, 125 MHz): δ (ppm) 139.0, 168.7, 176.0 (Figure S9).

Enzymatic bromination of 1–12

Analytical scale bromination reactions were performed in 500 µL volume containing 50 mM HEPES-Na (pH 7.5), 50 mM KBr, 0.01 mM Na_3VO_4, 1 µM CcVHPO1, and 1 mM individual **1–12** substrates. The reactions were initiated by adding 5 µL of the 10 mM hydrogen peroxide stock, and the tube was inverted multiple times for thorough mixing. The reaction proceeded at 30 °C for 1 h, during which an additional 1 µL of hydrogen peroxide was added every 10 min, totaling 5 µL of additional hydrogen peroxide and achieving a final concentration of 2 mM. After the 1 h incubation, 100 ng of catalase was added to quench the reaction and remove excess hydrogen peroxide, followed by incubation at room temperature for 30 min. The reaction was diluted 100× in MeOH before analysis by liquid chromatography/mass spectrometry (LC/MS).

For substrates **1**–**9**, LC was performed using an Agilent Poroshell 4.6×100 mm pentafluorophenyl (PFP) column with a 2.7 µm particle size at a 0.2 mL/min flow rate with solvent A: water+0.1% (v/v) formic acid (FA) and solvent B: MeCN+0.1% (v/v) FA. The elution profile was as follows: start at 95% A for 2 min; linear ramp to 95% B over 20 min; hold at 95% B for 2 min. MS spectra were acquired in the positive ion mode using an Agilent 6530C high resolution mass spectrometer equipped with an electrospray

ionization (ESI) source. The drying gas temperature was 250 °C at a flow rate of 8 L/min. Data were analyzed using the Agilent MassHunter software.

For substrates **10–12**, as well as the MS/MS characterization of substrate **4**, the LC parameters were the same, but an Agilent Poroshell C_{18} column with the same dimensions and particle size as above was used. MS^1 spectra were collected from 100 *m/z* to 1000 *m/z*. MS^2 spectra were acquired in the positive ion mode from 50 *m/z* to 3000 *m/z*. Various collision energies (10, 20, 30, and 40) were used to acquire collision induced disassociation (CID) MS^2 spectra.

Time course of bromination of 3

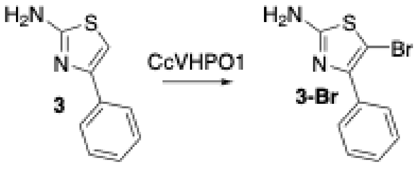

Five bromination reactions were performed in 500 µL reaction volume containing 50 mM HEPES-Na (pH 7.5), 50 mM KBr, 0.01 mM Na_3VO_4, 1 µM VHPO, and 1 mM **3**. The reaction was initiated by adding 10 µL of the 10 mM hydrogen peroxide stock, and the tube was inverted multiple times for thorough mixing, totaling in a 2 mM concentration. The reactions proceeded at 30 °C. At time points of 30, 60, 90, and 120 min, the reactions were quenched with 100 ng of catalase. The 0 min timepoint was immediately quenched. The quenched reactions were allowed to incubate at room temperature for 30 min. The reactions were analyzed by LC/MS as described above.

Preparative scale bromination of 1

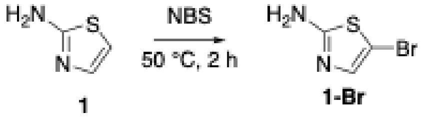

In a 15 mL reaction vessel, **1** (5 mg, 50 µmol) was added 500 µL HEPES-Na buffer (100 mM, pH 7.5), 2 mL KBr (90 mM), 50 µL Na_3VO_4 (0.1 mM), 300 µL of CcVHPO1 (2 µM, final concentration). The bromination reaction was initiated by adding 5 µL H_2O_2 followed by five additional 1 µL aliquots of H_2O_2 (18 mM, 2.0 equiv.) over the course of the reaction. The reaction was performed at 30 °C for 1 h in duplicate and combined post-completion. Upon completion, the reaction was quenched with catalase (1 µg). The resulting mixture was extracted with ethyl acetate (2×5 mL), and the combined organic layers were filtered

through anhydrous Na_2SO_4. The solvent was removed under reduced pressure, and the reddish-brown residue was reconstituted in $CDCl_3$ to record the ^{1}H-NMR spectra illustrated in Figure S10.

^{1}H NMR ($CDCl_3$, 500 MHz): δ 6.98 (s, 1H).

Preparative scale bromination of 3 and purification of 3-Br

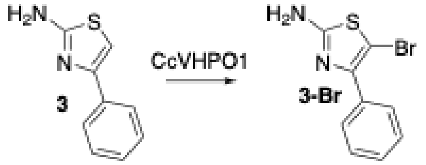

In 80 mL HEPES-Na buffer (100 mM, pH 7.5) was added **3** (20 mg, 112 μmole), 10 mM KBr, 0.01 mM Na_3VO_4, and 1 μM CcVHPO1. The bromination reaction was initiated by adding 2 mL H_2O_2 followed by five additional 200 μL aliquots of H_2O_2 (2 mM, 2.0 equiv.) over the course of the reaction. The reaction was allowed to proceed at 30 °C for 1 h. Upon completion, the reaction was quenched with catalase (1 μg), and the reaction mixture was extracted with EtOAc (3×50 mL). The organic layer was dried over Na_2SO_4, and the solvent was removed under reduced pressure using a rotary evaporator. The crude solid reaction mixture was purified using silica column chromatography. A gradient of 7–10% (v/v) of MeOH in $CHCl_3$ afforded the product **3-Br** as a brown solid in 52% isolated yield.

R_f(Hexane/EtOAc: 7:3) = 0.5

Isolated yield: 15 mg (52%)

^{1}H-NMR ($CDCl_3$, 400 MHz): δ (ppm) 5.18 (brs, 2H), 7.38 (d, J = 5 Hz, 1H), 7.38 (t, J = 10 Hz, 2H), 7.86 (d, J = 10 Hz, 2H) (Figure S30).

^{13}C-NMR ($CDCl_3$, 125 MHz): δ (ppm) 90.0, 128.3, 128.4, 128.6, 129.3, 130.4, 143.4, 168.1 (Figure S31).

Preparative scale bromination of 12 and purification of 12-Br

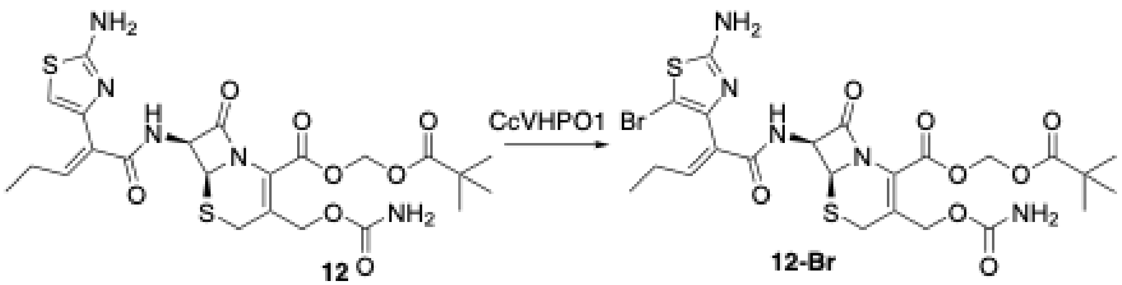

In a 50 mL reaction vessel, **12** (12 mg, 21 μmole) was added to HEPES-Na buffer (100 mM, pH 7.5), 10 mM KBr, 0.1 mM Na_3VO_4, and 1 μM CcVHPO1. The bromination reaction was initiated by adding 20 μL H_2O_2 followed by five additional 2 μL aliquots of H_2O_2 (2 mM, 2.0 equiv.) over the course of the reaction at 30 °C for 1 h. Upon reaction completion, the reaction was quenched with catalase (1 μg), and the reaction mixture was extracted with EtOAc (3×25 mL). The organic layer was dried over Na_2SO_4, and the solvent was removed under reduced pressure using a rotary evaporator. The crude solid reaction mixture was purified using silica column chromatography. 15–25% (v/v) EtOAc in hexane afforded the product **12-Br** as a brown solid with 58% isolated yield.

R_f(MeOH/$CHCl_3$: 1:9) = 0.5

Yield: 8 mg (58%)

^{1}H-NMR (Methanol-d_4, 500 MHz): δ (ppm) 1.12 (t, J = 7.6 Hz, 3H), 1.23 (s, 9H), 2.4 (m, 2H), 3.56 (d, J = 18.6 Hz, 1H), 3.7 (d, J = 18.6 Hz, 1H), 4.7 (d, J = 13.6 Hz, 1H), 5.1 (d, J = 13.6 Hz, 1H), 5.18 (d, J = 4.9 Hz, 1H), 5.85 (d, J = 5.7 Hz, 1H), 5.91 (d, J = 4.5 Hz, 1H), 5.95 (d, J = 5.7 Hz, 1H), 6.48 (t, J = 8 Hz, 1H) (Figure S52).

^{13}C-NMR spectra (Methanol-d_4, 125 MHz): δ (ppm) 12.6, 22.8, 25.8, 29.3, 38.4, 57.5, 59.4, 62.7, 79.6, 89.9, 124.1, 129.0, 141.8, 144.2, 157.8, 160.4, 164.6, 167.8, 168.6, 177.0 (Figure S53).

Suzuki-Miyaura cross coupling (SMCC) reactions for arene addition to 3-Br

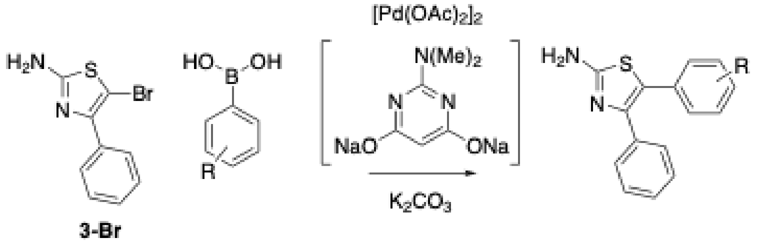

3-Br

The synthesis of the ligand 2-dimethylamino-4,6-dihydroxypyrimidine (DMADHP) has been described before.[5] In a 1.5 mL microcentrifuge tube, **3-Br** (1 μmol), aryl boronic acid (0.4 mg, 4 μmol), potassium carbonate (0.8 mg, 6 μmol) was suspended in water/MeCN (4:1 (v/v), 1.0 mL). A solution of DMADHP-Pd in water (5 mol%, 10 μL) was added. The vial was stirred at 30 °C until reaction completion in open-air condition. The reaction was diluted with MeCN (100 μL supplemented with 2% (v/v) 3-mercaptopropionic acid) and analyzed by LC/MS.

The LC was performed using an Agilent Poroshell 2.7 µm 4.6×100 mm C_{18} column at a 0.3 mL/min flow rate. The elution profile started with 5% B for 2 min, followed by the linear ramp to 95% B over 27 min. MS spectra were acquired in the positive ionization mode.

SMCC reactions for arene addition to 4-Br

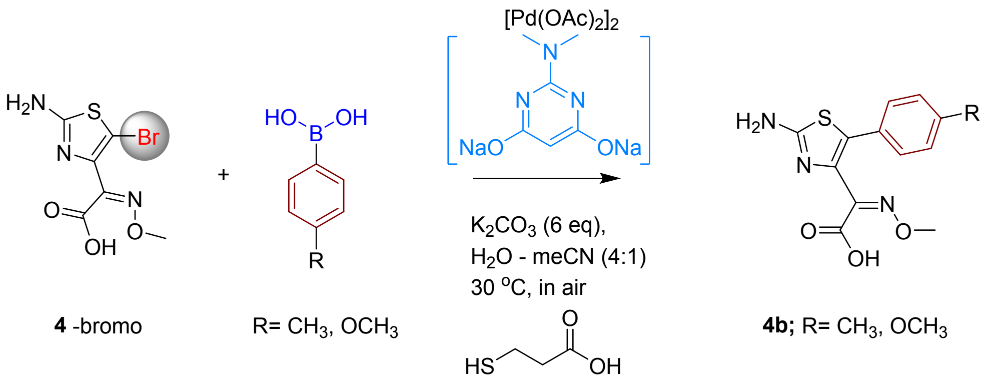

In a 1.5 mL microcentrifuge tube, **4-Br** (1 µmol), aryl-B(OH)$_2$ (0.4 mg, 4 µmol), potassium carbonate (0.8 mg, 6 µmol) was suspended in water/MeCN (4:1, 1.0 ml). A solution of DMADHP-Pd in water (5 mol%, 10 µL) was added. The vial was closed and stirred at 30 °C until reaction completion. The reaction was diluted with MeCN (100 µL supplemented with 2% (v/v) 3-mercaptopropionic acid) and analyzed LC/MS.

The LC was performed using an Agilent Poroshell 2.7 µm 4.6×100 mm C_{18} column at flowrate of 0.3 mL/min. The elution profile started with 5% B for 2 min, followed by the linear ramp to 95% B over 27 min. MS spectra were acquired in the positive ionization mode.

SMCC reactions for arene addition to 10-Br

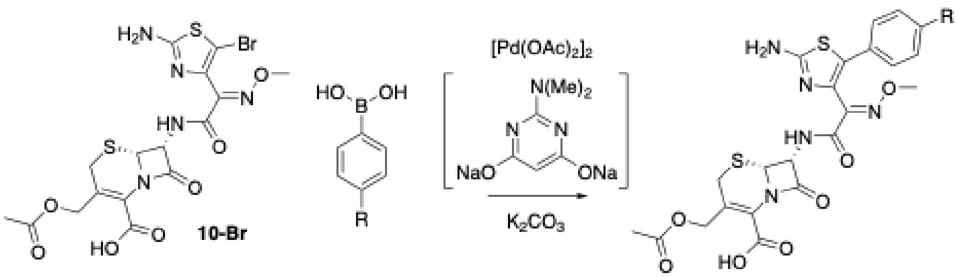

In a 1.5 mL microcentrifuge tube, appropriate **10-Br** (1 µmol), aryl-B(OH)$_2$ (0.4 mg, 4 µmol), potassium carbonate (0.8 mg, 6 µmol) was suspended in water/MeCN (4:1, 1.0 ml). A solution of DMADHP-Pd in water (5 mol%, 10 µL) was added. The vial was closed and stirred at 30 °C until reaction

completion. The reaction was diluted with MeCN (100 μL of 2% (v/v) 3-mercapto propionic acid) and analyzed by LC/MS.

The LC was performed using an Agilent Poroshell 2.7 μm 4.6×100 mm C_{18} column at a 0.3 mL/min flow rate. The elution profile started with 5% B for 2 min, followed by the linear ramp to 95% B over 27 min. MS spectra were acquired in the positive ionization mode.

SMCC reactions for arene addition to 12-Br

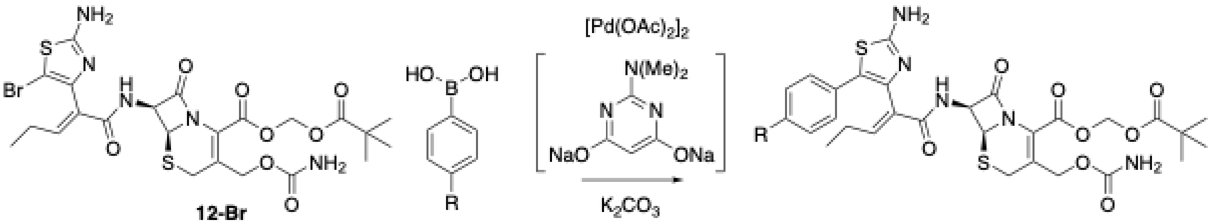

In a 1.5 mL microcentrifuge tube, appropriate **12**-bromo (1 μmol), aryl-$B(OH)_2$ (0.4 mg, 4 μmol), potassium carbonate (0.8 mg, 6 μmol) was suspended in water/MeCN (4:1, 1.0 ml). A solution of DMADHP-Pd in water (5 mol%, 10 μL) was added. The vial was closed and stirred at 30 °C until reaction completion. The reaction was diluted with MeCN (100 μL of 2% (v/v) 3-mercapto propionic acid) and analyzed by LC/MS.

The LC was performed using an Agilent Poroshell 2.7 μm 4.6×100 mm C_{18} column at a 0.3 mL/min flow rate. The elution profile started with 5% B for 2 min, followed by the linear ramp to 95% B over 27 min. MS spectra were acquired in the positive ionization mode. Data analysis was performed using Agilent MassHunter software.

Preparative scale SMCC reaction for *p*-methoxyphenylboronic acid addition to 3-Br

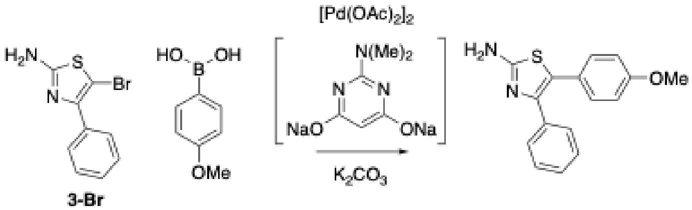

In a 100 mL reaction vessel, **3-Br** (10 mg, 40 μmol), *p*-methoxyphenylboronic acid (30 mg, 200 μmol), potassium carbonate (41.5 mg, 300 μmol) was suspended in water/MeCN (4:1 v/v, 25 mL). A solution of DMADHP-Pd in water (5 mol%, 500 μL) was added. The vial was left open and stirred at 30

°C for 48 h. The reaction progress was monitored by thin layer chromatography (TLC). Then, the reaction mixture was extracted with EtOAc (3×20 mL). The organic layers were combined and dried over Na_2SO_4. The solvent was removed using a rotary evaporator. The crude solid reaction mixture was purified using isocratic silica column chromatography using 5% (v/v) MeOH in $CHCl_3$, affording the product as a brown gummy solid with 26% isolated yield (3 mg).

R_f(MeOH/$CHCl_3$: 1:9) = 0.4

^{1}H-NMR ($CDCl_3$, 500 MHz): δ (ppm) 3.37 (s, 3H), 5.14 (brs, 2H), 6.94 (m, 3H), 7.0 (d, *J* = 8.5 Hz, 2H), 7.72 (d, *J* = 8.4 Hz, 1H), 7.78 (d, *J* = 8.6 Hz, 1H), 8.2 (d, *J* = 8.5 Hz, 2H) (Figure S47).

Supplementary Tables

Table S1: Primary sequences and GenBank accession numbers for VHPOs used in this study

ccVHPO1	MSAARRQEAFQTRVRAARLARDRDHQVFEPNGEEDRYFRPNSQIFSYLGSYTKG LPHNRRTALITSPNHFVRFVRAIDSGEPADFIKVPLGPRPALQFESEEECRAANLFK SGIAGRNNTFPSSCLRAWESMGAGLVFDLEGPDAQAVGMPPAPTLDSDELVAEIS EVYWMALLRDVRFTDFTNPGPIGRAINTLNKTAWIRAARDPPDSLTPQERARLRG PFNPSNIFRGTLPGDDVGPYLSQFLLVGTKGIGGAQDMADGFISYGAHRIDQRVR HARKELDYMTSWEAFLDVQNGADVTGRDTFESGSDAFRFITTPRDLATYVHFDA LYQAYLNACLIMLDSGVRFDKGIPFGEPDFKDHQRGFAHFGGPHILSLVTEVATR ALKAVRFQKFNTHRRLRPEAVGGLIERFNSNPDDPQFQDVKPLFEALDEDMMRR VARHNREQNQGSDFGMPRADDFNPAGDTLETMLLPMAFPEGSPMHPSYGAGHA TVAGACVTVLKAFFQHDTELDFCFVPSDDGSRLVDASHNMNKKLTVEGELNKV CSNISIGRNWAGVHYFTDYIESILLGEQIALGILEEQMLTFPETFTMTVPLFSGELK VLRST<br><br>GenBank accession number XP_005714237.1
ccVHPO2	MATPCFAQAPAPTLRRSPRRLPLTPTSFLPRPFLPKPFPRSPRHPHPRLNAASARTS AAQPPDRAATPCATRQQQALELRRAAAQLAHDRAHPAHVSNGSEELHLHPKTG APLLLANFTKGLPHERDTGLAADAAHYDLFVAAVHSGLPDDIRAIPLGPQAAPGV RLPRSLRFRSGIARSSHLFPHTDVRAWESMAAGNAFDLQGPDAQAVTMPPAPAL ASPELALEMTENYWMALLRDVRFSQFAADPTVAAAMESMNATEWVALACGDA SAVAEEERKRLRGPFTAQNVFRGNTQGDCKGPYISQFLLSGSAGLGQVNSGADG YIQYGAMRMDQRVRVALEGLDYMTTWESWLDVQNGADLRGRELYEDGERFRFI CTPRDLATYVHYDALYQAYLNACIIMLNKKIPFDKGLPFQKDDDIDKQQGFALFG PPHILTLCTEVATRALKGIRFQKYNVHRRLRPEAVGGRVERYHHNCEDPLFADVK PLYDALDKDMLERVAAHNAEQNAKDDFGNSRFEDYSPTGSSGRTYLLPMAFPEG SPMHPAYGAGHAAVAGACTTILKAFFDHEHELDFAYVPTADGSKLEDVVDSLGE KLTVEGELNKVCSNISVGRNWAGVHYFTDYRESIILGEKIALGLLEEQKLTYAEEF SMTIPLFDGSTVTI<br><br>GenBank accession number XP_005719435.1
AtMbb1	MTDTQNPNRAEVAFKVRVSAAELARARGSPAHLSNNSESRFRNPDGTRSLLANF TKGLPHIKETALVESAIDYDRFVRAIDSGDPRDFADLPLGPQGVEPRFTSGIASDPE VGTRAWESGGAGLVFDLEGPDAQAVTMPPAPELDSDELVAEVTECYWMSLLRD VPFPTFESNSHIQAAAESINNTQWIKFKNNPPAHLTAAERSRLRGPVTTANVFRGI TPGDEVGPYLSQFLLVGTTGIANGNEVGDGFIQYGGMRMDQRVRVAKPHIDYMT TFGAYLDVQNAANVSGRELYNEEEPRFRFIHTPRDLATYVHFDALYQAYLNACII LLDIGAPFDSGIPFQLDNDIDKQQGFATFGGPHILSLVTEVATRALKAVRFQKFNV HRRLRPEAIGARVDRYCATKAPEFAGAAKLSEALDKELLQKVHDHNKKQNLLSD RGNPRANDFNPDGEVSEGNLLMPMAFPEGSPMHPAYGAGHATVAGACVTVLKA FFDGGYRLPFCYITDEDGTGLQAVEIDEPLTVDGELNKICSNISIGRNWAGVHYFT DYIESIRIGEEIAIGILQEQKLTFSENFSMTLNKFDGSTIRI<br><br>GenBank accession number QKI80102.1
AtMbb4	MAEERRQNALEIRIQAAKLAKKRDHPTHKANGDEDRYPETLIGSFTKGLPHEKET GLLSNPADFADFVRAINTGAIKDMRRLKLGIDEDPRFISGIASKGDKHPFADTRAW

	ESMAAGLTYDLEGPDAQAVTMPPAPKLDSDELVTEITESYWMALLRDVPFTEFE<br>RDGNTAAAAASISRTRWVQYNEQPSQRPSTLTDEEIARLRGPYTKKNVFRGVTN<br>GENVGPYLSQFLLVGTKGIGDAQQVSDGYVQYGGMRMDQRVRVAVPKRDYMT<br>TWASWLDVQNAGDLRGREIYDDDTPFRFITTPRDLATWVHFDALYQAYLNACIIL<br>LDIKAPFDPHIPFQADDDVDKQQGFATFGGPHILSLCTEVATRALKAVRFQKYNL<br>HRRLRPEAIGGLVERFKKTNGDPKFAPVKKLVNDLDGDMLRRVEQHNCEQNKLS<br>DDGHARREDYSPEGESSQSYLLPMAFPEGSPMHPSYGAGHATVAGACVTVLKAF<br>FDHEYELDFCYVPTTDGKRLEKVNINEKLTVEGELNKLCANISIGRNWAGVHYYS<br>DYFESIKVGEEIAIGILQEQKLTYGEDFFMTLPKFDGEKIRI<br><br>GenBank accession number QKI80105.1

Table S2: Mass spectrometric detection of brominated products generated by CcVHPO1

substrate	Brominated product[a]	Brominated product formula	*m/z* theoretical	*m/z* observed	Conversion [b](%)
**1**	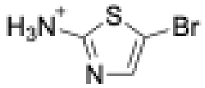	$C_3H_4BrN_2S^+$	178.9273	178.9281	94
**2**	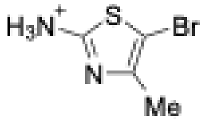	$C_4H_7N_2S^+$	192.9430	192.9425	46
**3**	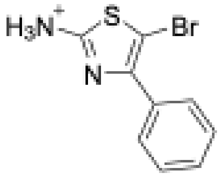	$C_9H_8BrN_2S^+$	254.9586	254.9583	90<br><br>isolated yield 52%
**4**	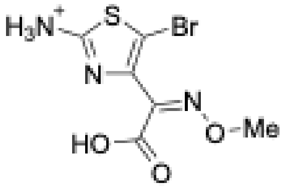	$C_6H_7BrN_3O_3S^+$	279.9386	279.9390	94
**5**	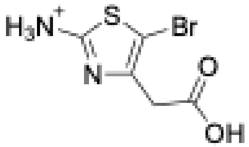	$C_9H_{12}BrN_2O_3S^+$	236.9328	236.9321	>95
**6**	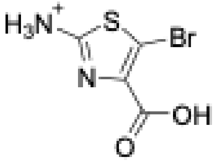	$C_4H_4BrN_2O_2S^+$	222.9171	222.9164	26
**7**	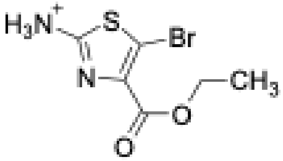	$C_6H_8BrN_2O_2S^+$	250.9485	250.9485	>95
**8**	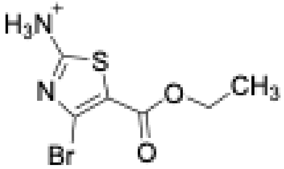	$C_6H_8BrN_2O_2S^+$	250.9485	n.d.[c]	
**9**	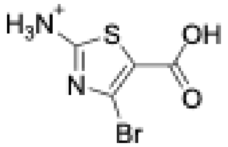	$C_4H_5N_2O_2S^+$	222.9171	n.d.	

**10**	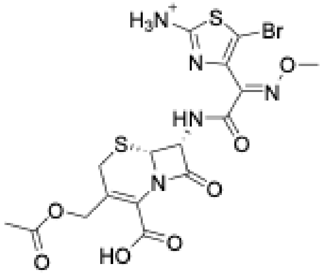	$C_{16}H_{17}BrN_5O_7S_2^+$	533.9747	533.9739	65
**11**	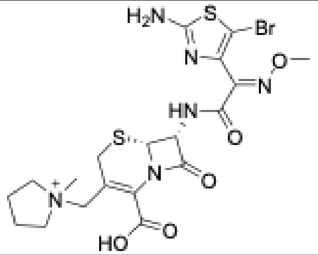	$C_{19}H_{24}BrN_6O_5S_2^+$	559.0427	559.0412	78
**12**	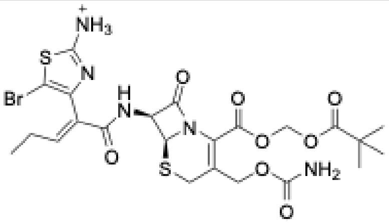	$C_{23}H_{29}BrN_5O_8S_2^+$	646.0635	646.0631	71<br>isolated yield 58%

[a] structures are illustrated in their singly ionized states

[b] conversions, expressed as % values, were determined using the area under the peaks in extracted ion chromatograms (EICs)

[c] brominated product not detected

Table S3: Mass spectrometric detection of Suzuki-Miyaura cross coupling (SMCC) products and yields of SMCC reactions

SMCC product structure	Formula	*m/z* theoretical	*m/z* observed	Conversion[a](%)
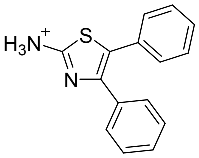	$C_{15}H_{13}N_2S^+$	253.0794	253.0798	40
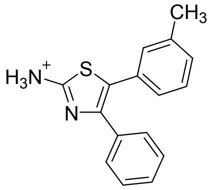	$C_{16}H_{15}N_2S^+$	267.0951	267.0964	52
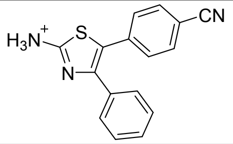	$C_{16}H_{15}N_2S^+$	278.0747	278.0757	32
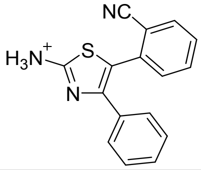	$C_{16}H_{12}N_3S^+$	278.0747	278.0779	45
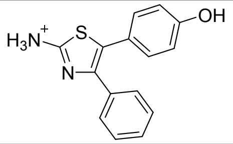	$C_{15}H_{13}N_2OS^+$	269.0744	269.0748	80
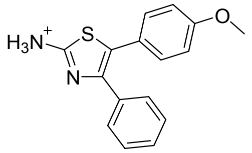	$C_{16}H_{15}N_2OS^+$	283.0900	283.0927	86
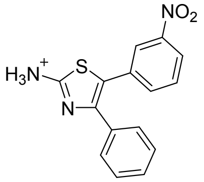	$C_{15}H_{12}N_3O_2S^+$	298.0645	298.0650	41
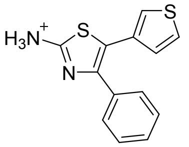	$C_{13}H_{11}N_2S_2^+$	259.0359	259.0379	84
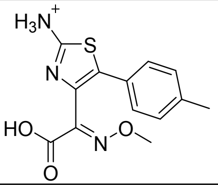	$C_{13}H_{14}N_3O_3S^+$	292.0750	292.0755	70

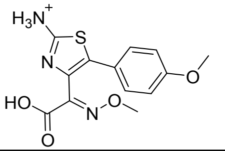	$C_{13}H_{14}N_3O_4S^+$	308.0700	308.0697	42
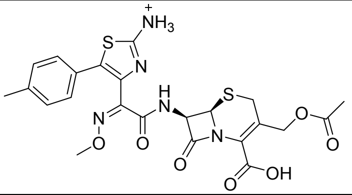	$C_{23}H_{24}N_5O_7S_2^+$	546.1112	546.1103	82
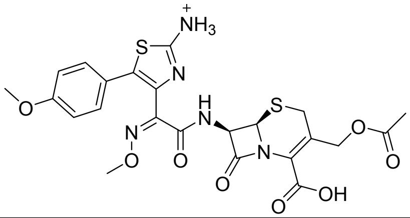	$C_{23}H_{24}N_5O_8S_2^+$	562.1061	562.1080	86
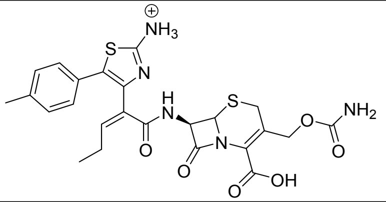	$C_{24}H_{26}N_5O_6S_2^+$	544.1319	544.1298	79
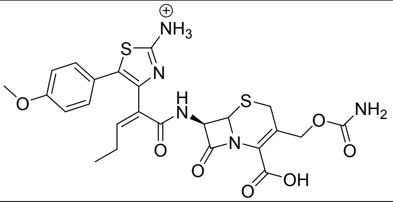	$C_{24}H_{26}N_5O_7S_2^+$	560.1268	560.1251	76

[a] conversions, expressed as % values, were determined using the area under the peaks in extracted ion chromatograms (EICs)

SUPPLEMENTARY FIGURES

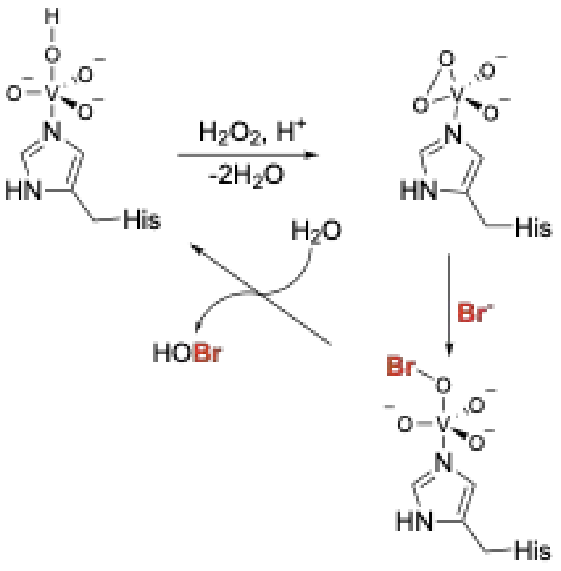

Figure S1: The presently accepted catalytic mechanism for bromide oxidation and hypobromous acid production by vanadium-dependent haloperoxidases (VHPOs) wherein the vanadate cofactor is covalently ligated to a His side chain imidazole in the enzyme active site. Note that the 2e⁻ oxidation of the halide is accompanied by the reduction of H_2O_2.

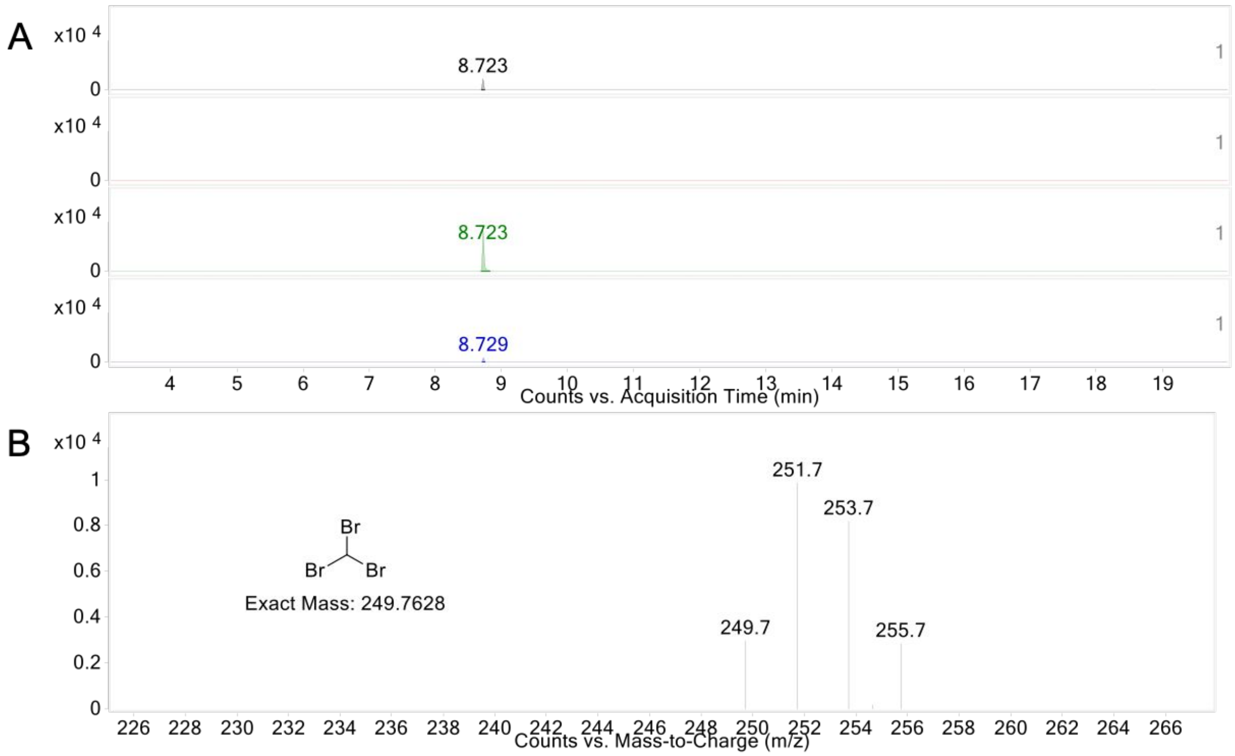

Figure S2: (**A**) GC/MS extracted ion chromatograms (EICs) (*m/z* 251.5–252.0) demonstrating relative abundance of bromoform detected to be produced in *in vitro* reactions using 2,4-pentanedione as the hydrocarbon substrate by the four VHPO enzymes used in this study. 2,4-pentanedione has been established as a viable substrate for bromoform production.[1, 6] From top to bottom, the VHPOs employed are: AtMbb1, AtMbb4, CcVHPO1 and CcVHPO2. The reactions were extracted twice with diethyl ether and the solvent removed *in vacuo*. Samples were reconstituted in EtOAc for analysis. GC/MS was performed using an Agilent 8860A GC equipped with a WCOT DB5 column. The samples were injected with a volume of 1 µL with a flow rate of 1.2 mL/min. The temperature gradient started at 40 °C with a hold time of 3 min before increasing to 200 °C over the course of 10 min. Then, it held for 1 min. The MS was performed with an Agilent 5977 MSD. Data were analyzed using the Agilent MassHunter software. Note that CcVHPO1 offers the maximum bromoform production. (**B**) MS[1] spectra for bromoform demonstrating the characteristic isotopic signature of tribrominated species.

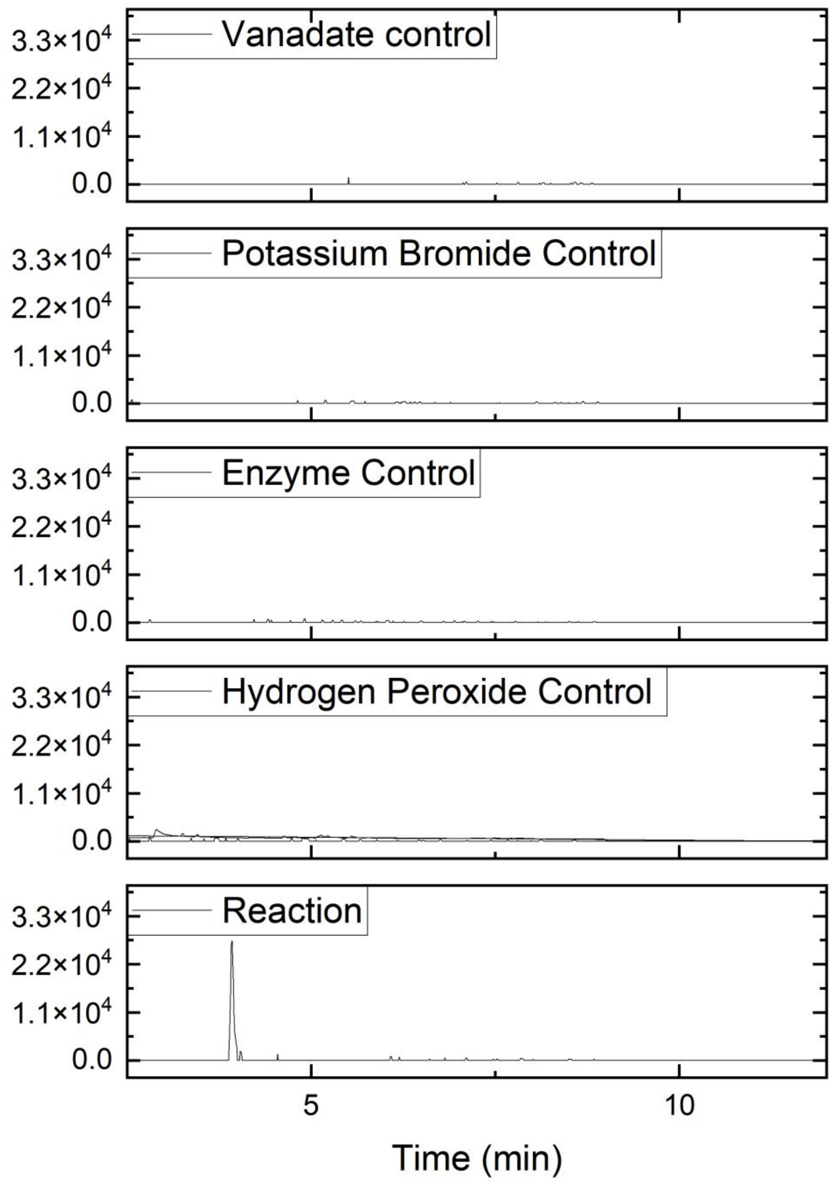

Figure S3: EICs (*m/z* 251.5–252.0) for negative control reactions demonstrating lack of production of bromoform as compared to the reaction with all components added (bottom panel). The components omitted for each individual reaction are listed. The reactions were extracted twice with diethyl ether and the solvent removed *in vacuo*. Samples were reconstituted in EtOAc for analysis. GC/MS was performed using an Agilent 7890A GC equipped with a WCOT DB5 column. The samples were injected with a volume of 1 µL with a flow rate of 1.2 mL/min. The temperature gradient started at 50 °C with a hold time of 3 min before increasing to 300 °C over the course of 5 min. Then, it held for 10 min. The MS was performed with an Agilent 5975 MSD. Data were analyzed using Agilent Enhanced Data Analysis software.

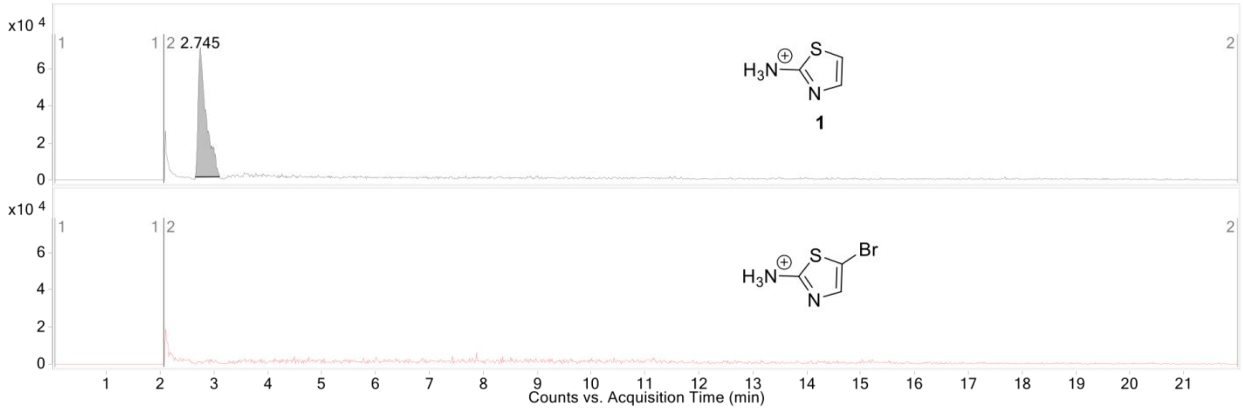

Figure S4: [M+H]$^{1+}$ EICs demonstrating presence of substrate **1** (top panel) and monobrominated product (bottom panel) detected to be produced in a negative control enzymatic reaction wherein the catalyst CcVHPO1 was omitted from the reaction.

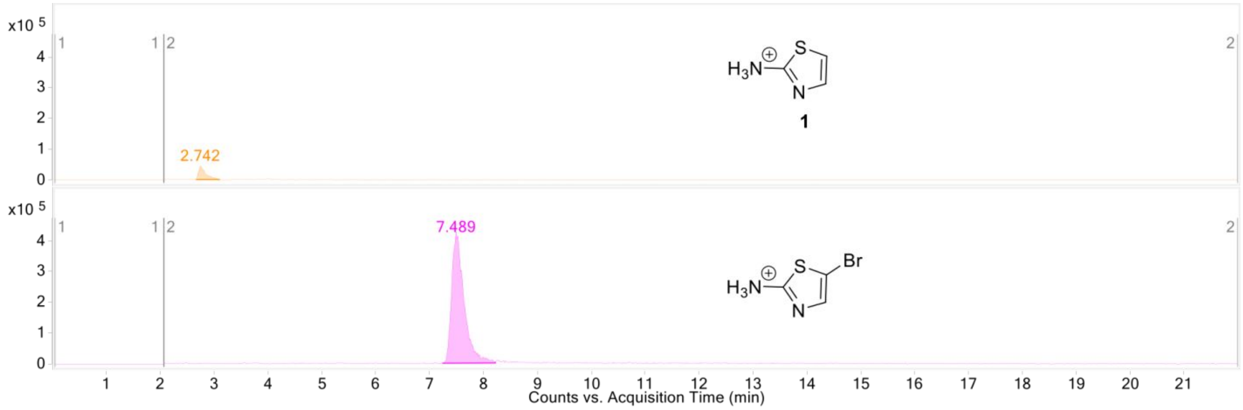

Figure S5: [M+H]$^{1+}$ EICs demonstrating presence of substrate **1** (top panel) and monobrominated product (bottom panel) detected to be produced in an enzymatic reaction using CcVHPO1. The brominated product has a higher retention time as compared to the substrate. The %conversion listed in Table S2 and in Figure 2 in the manuscript are calculated using the areas under the EIC peaks corresponding to the substrate and the product.

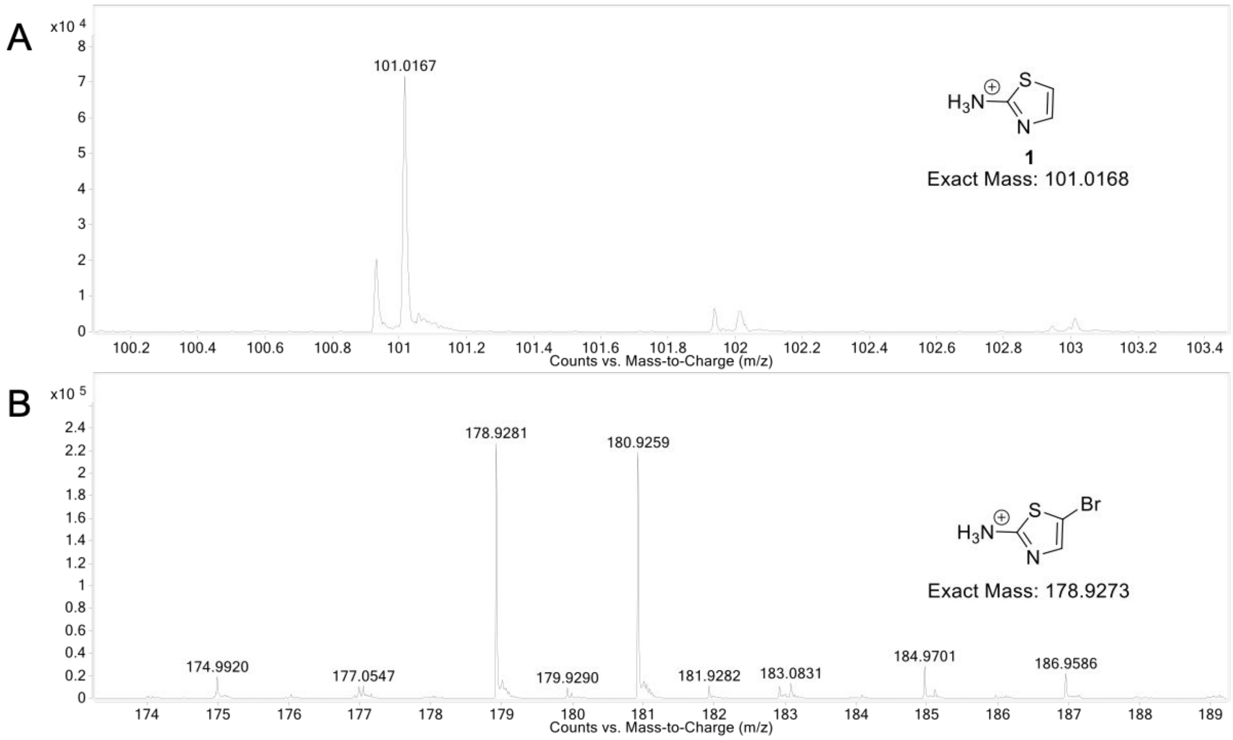

Figure S6: (**A**) $[M+H]^{1+}$ MS^1 spectra of **1**. (**B**) $[M+H]^{1+}$ MS^1 spectra for **1-Br**.

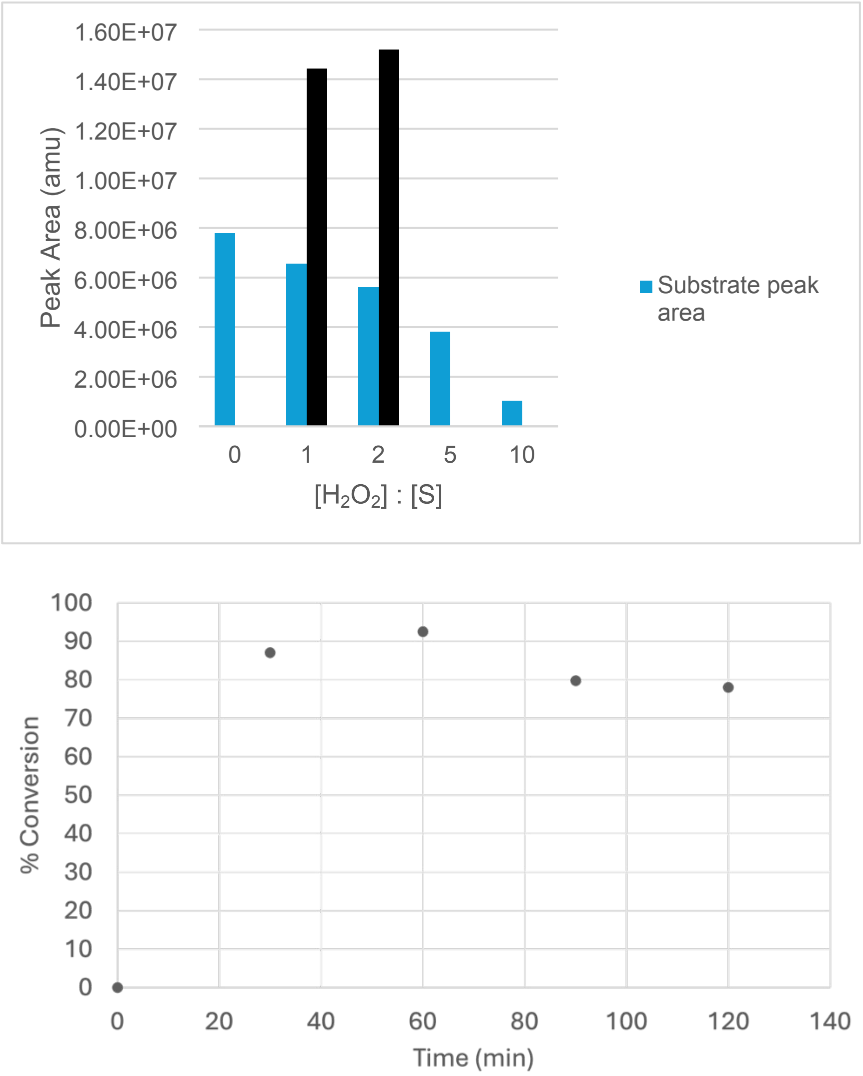

Figure S7: (top) Histogram of product yield vs relative hydrogen peroxide to substrate ratio. Area under the curve values taken from EIC's of substrates (EIC: *m/z* 101.0168) and product (*m/z* 178.9274). (bottom) %Conversion of substrate to product with different reaction times. Note that the highest conversion was observed at 1 h reaction time.

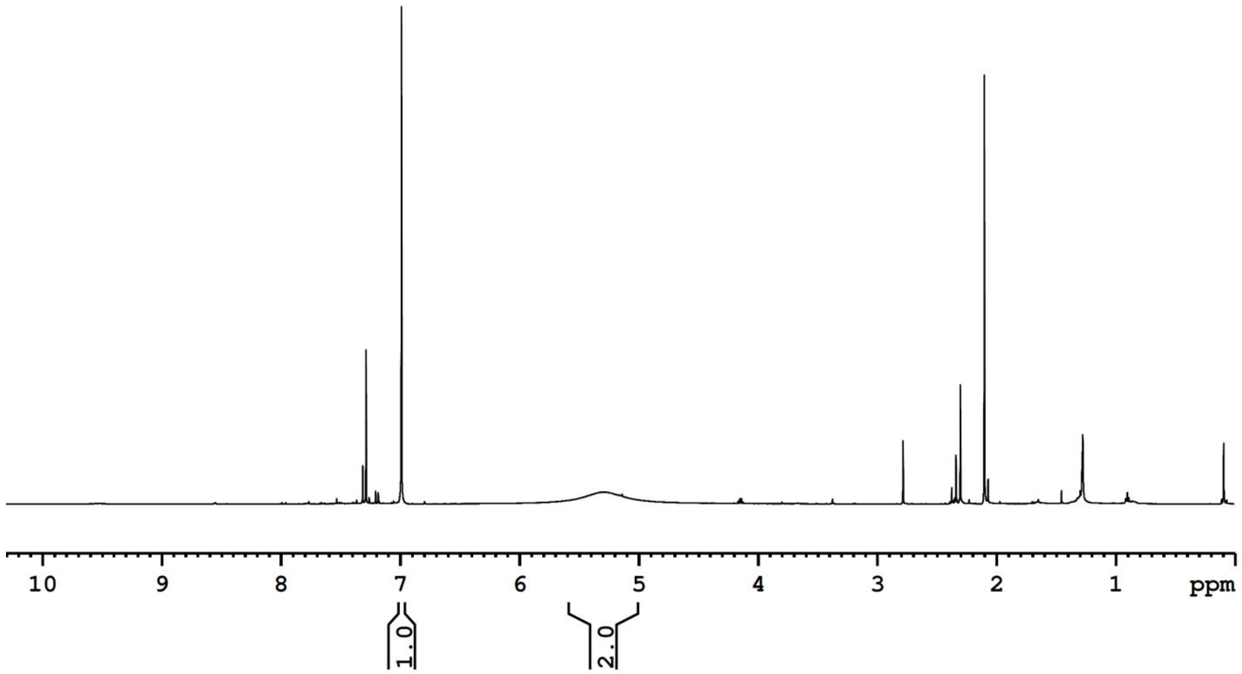

Figure S8: ^{1}H-NMR spectrum ($CDCl_3$, 500 MHz) of synthetic **3-Br**.

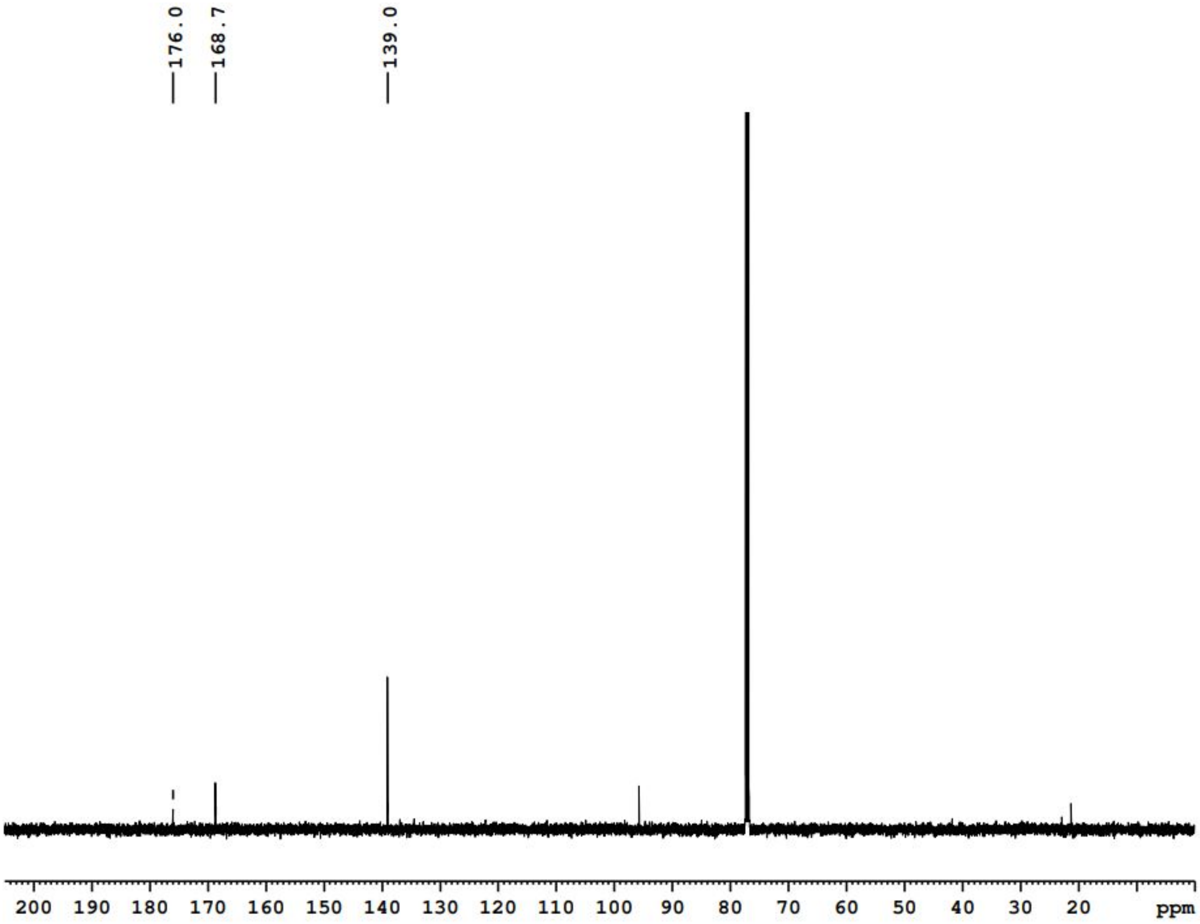

Figure S9: $^{13}C\{^{1}H\}$ NMR spectrum ($CDCl_3$, 125 MHz) of synthetic **3-Br**.

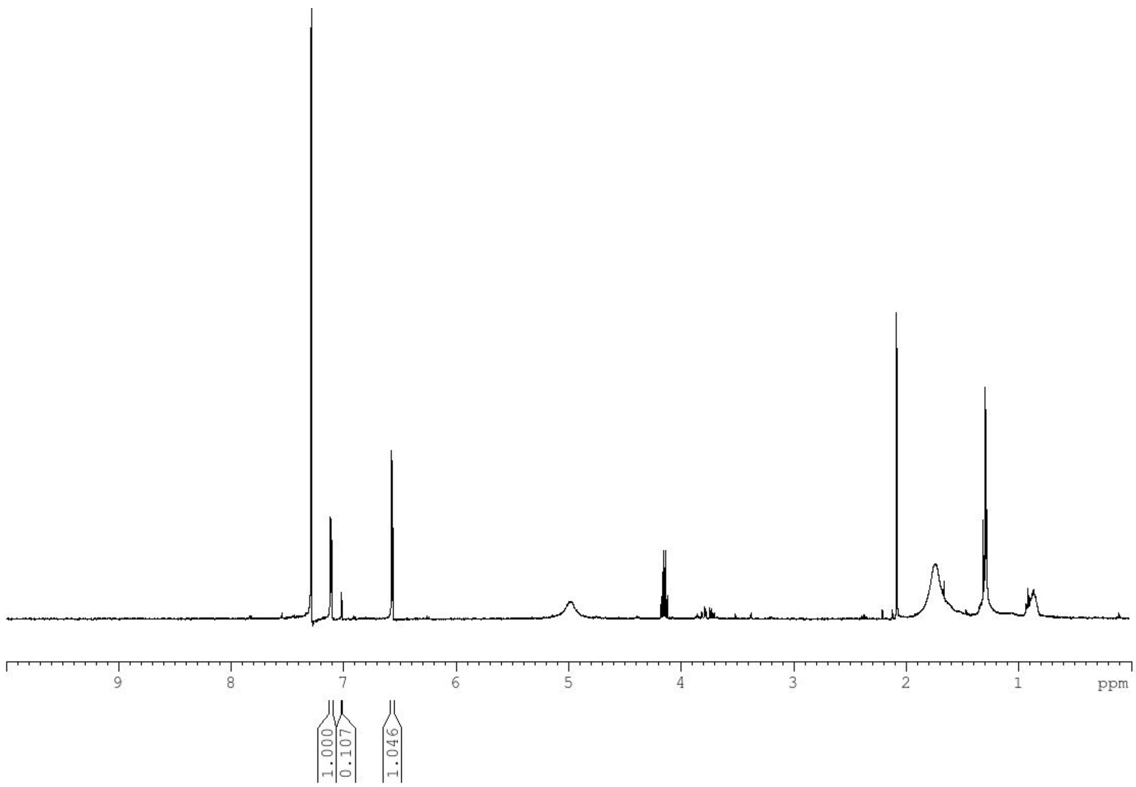

Figure S10: ^{1}H NMR spectrum ($CDCl_3$, 500 MHz) of the reaction mixture of VHPO-catalyzed bromination of **1** at a preparative scale. The brominated product is found to be formed by comparing the singlet at δ 6.98 ppm.

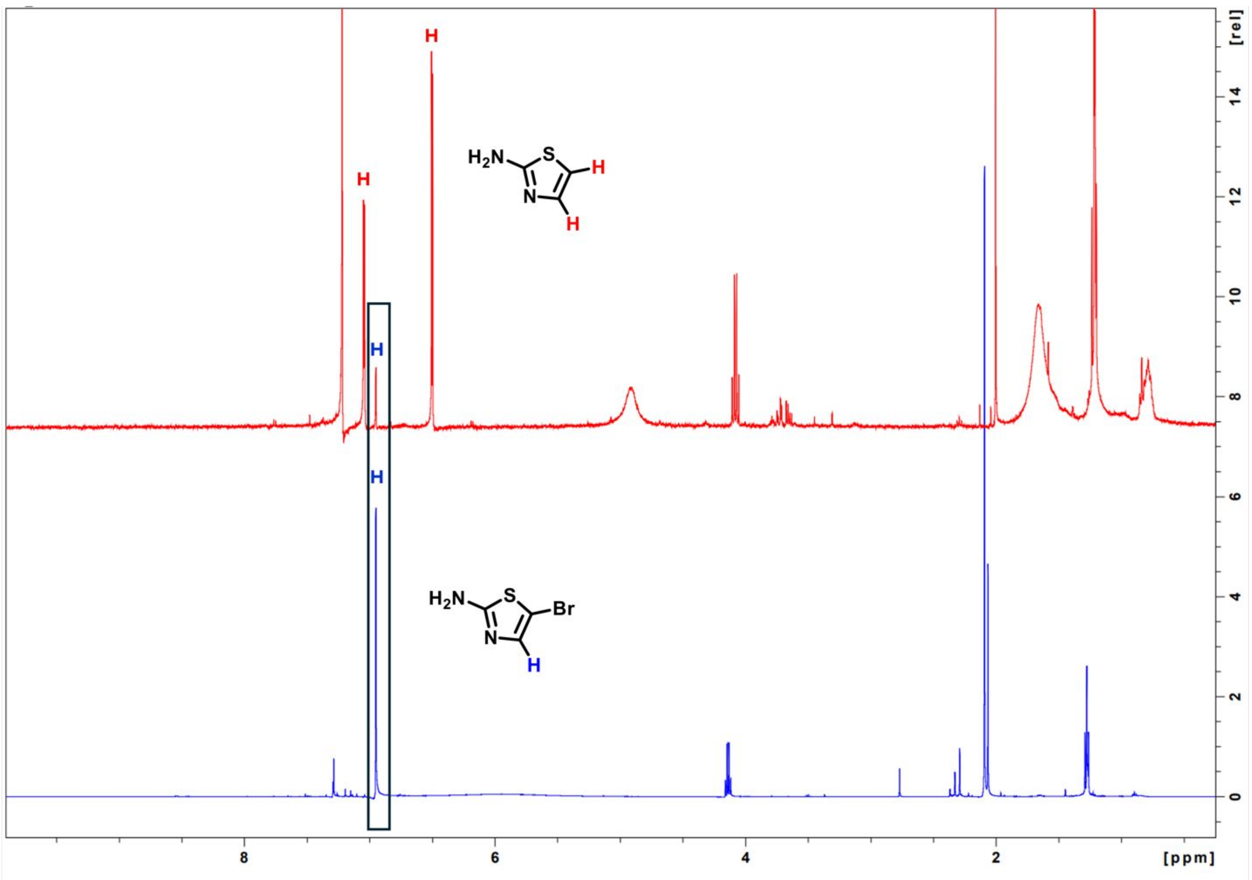

Figure S11: Overlaid ^{1}H-NMR spectra for the enzymatic product(s) generated by CcVHPO1-catalyzed bromination of **1** (in red, top), and synthetic standard of brominated product generated starting from **1**. The singlet that characterizes the brominated product is boxed.

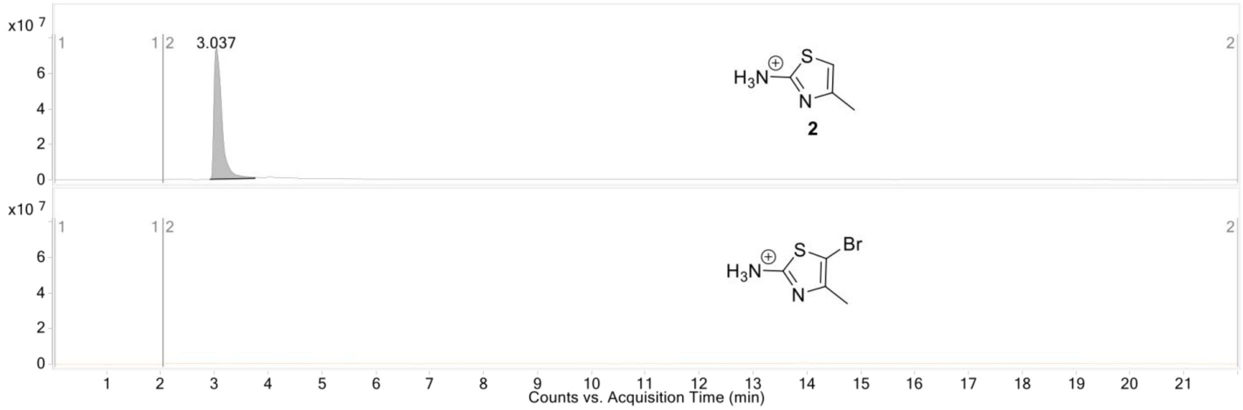

Figure S12: [M+H]$^{1+}$ EICs demonstrating presence of substrate **2** (top panel) and monobrominated product (bottom panel) detected to be produced in a negative control enzymatic reaction wherein the catalyst CcVHPO1 was omitted from the reaction.

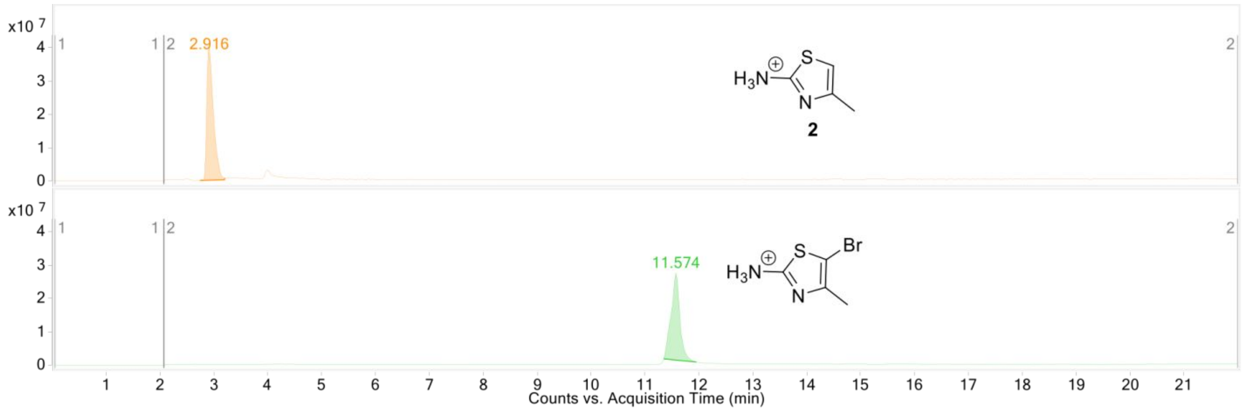

Figure S13: [M+H]$^{1+}$ EICs demonstrating the presence of substrate **2** (top panel) and monobrominated product (bottom panel) detected to be produced in an enzymatic reaction using CcVHPO1. The brominated product has a higher retention time than the substrate.

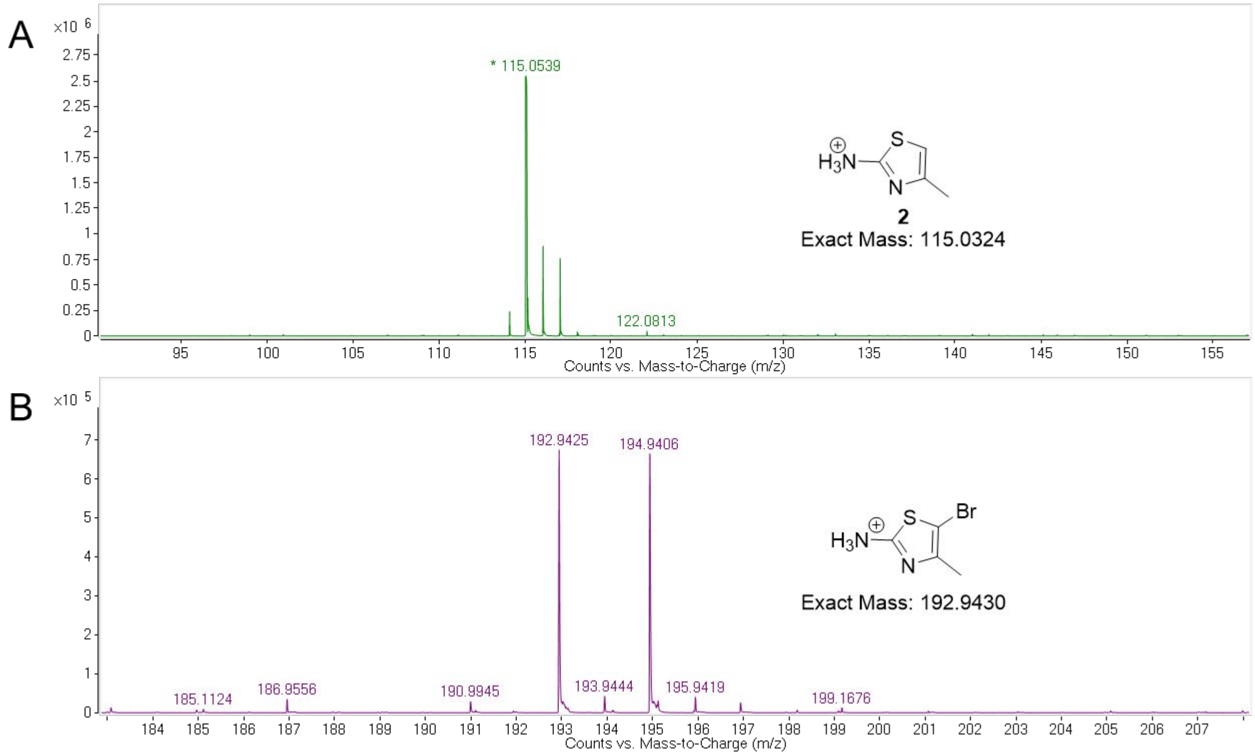

Figure S14: (**A**) [M+H]$^{1+}$ MS^1 spectra of **2**. (**B**) [M+H]$^{1+}$ MS^1 spectra for mono brominated **2**.

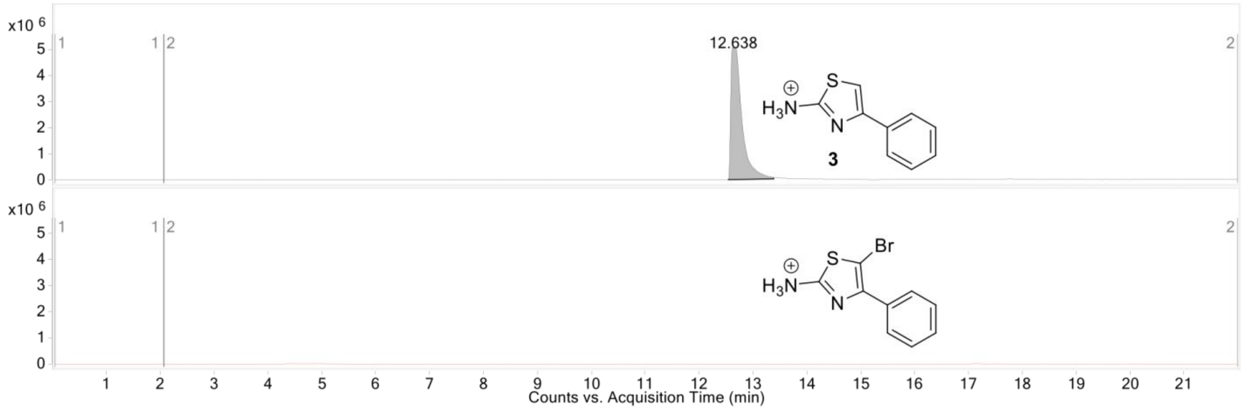

Figure S15: [M+H]$^{1+}$ EICs demonstrating the presence of substrate **3** (top panel) and monobrominated product (bottom panel) detected to be produced in a negative control enzymatic reaction wherein the catalyst CcVHPO1 was omitted from the reaction.

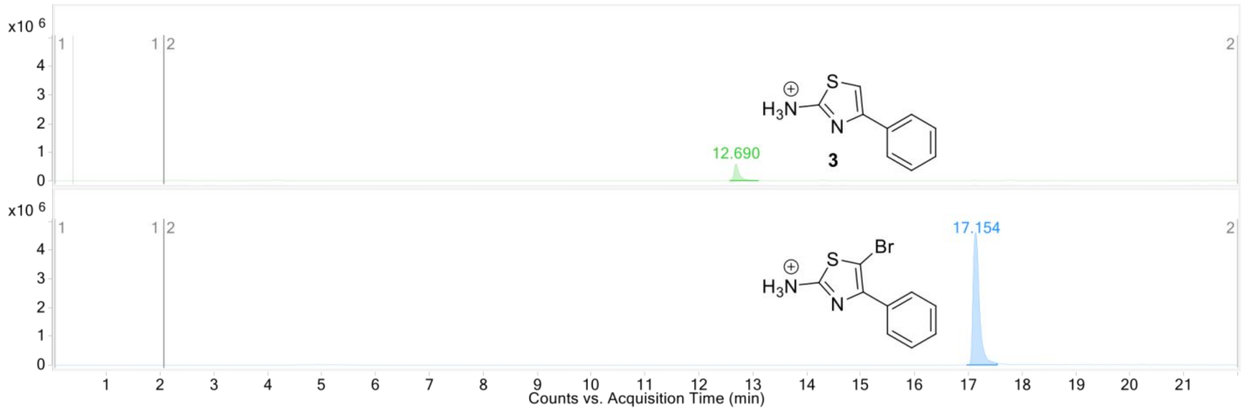

Figure S16: [M+H]$^{1+}$ EICs demonstrating the presence of substrate **3** (top panel) and monobrominated product (bottom panel) detected to be produced in an enzymatic reaction using CcVHPO1. The brominated product has a higher retention time than the substrate.

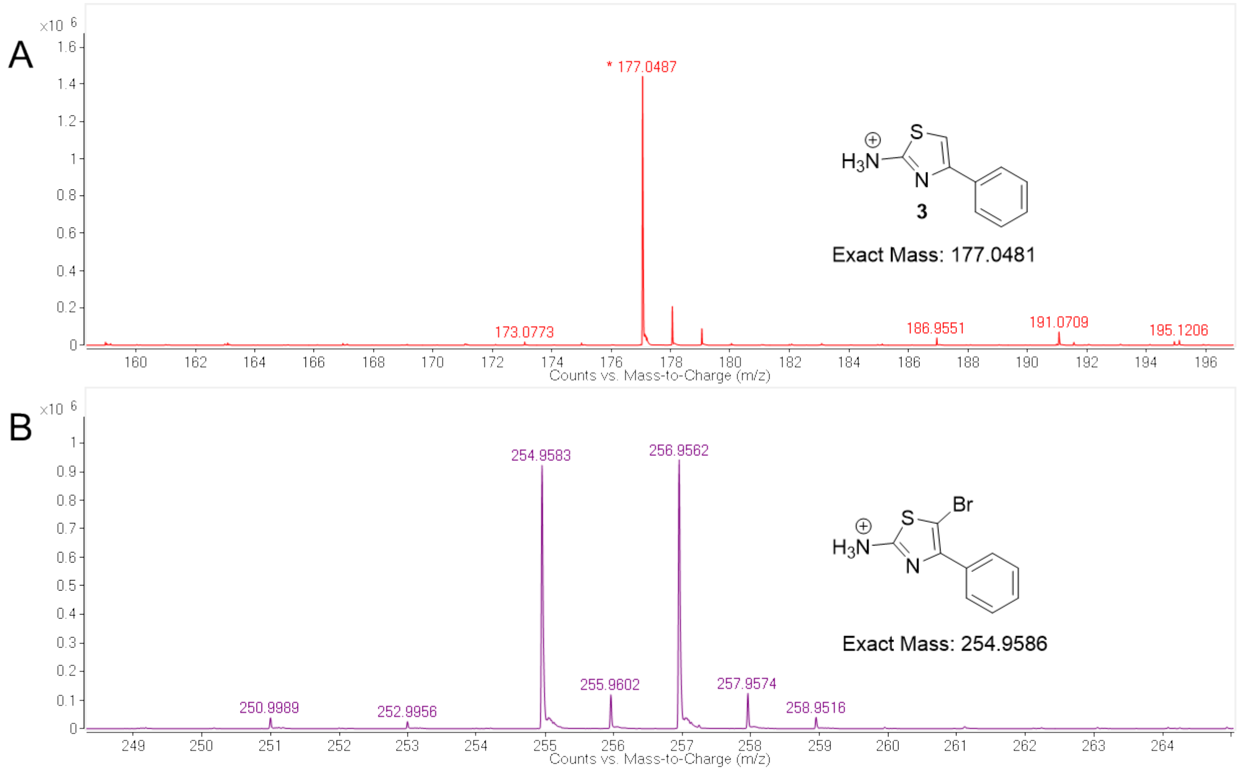

Figure S17: (**A**) $[M+H]^{1+}$ MS^1 spectra of **3**. (**B**) $[M+H]^{1+}$ MS^1 spectra for mono brominated **3**.

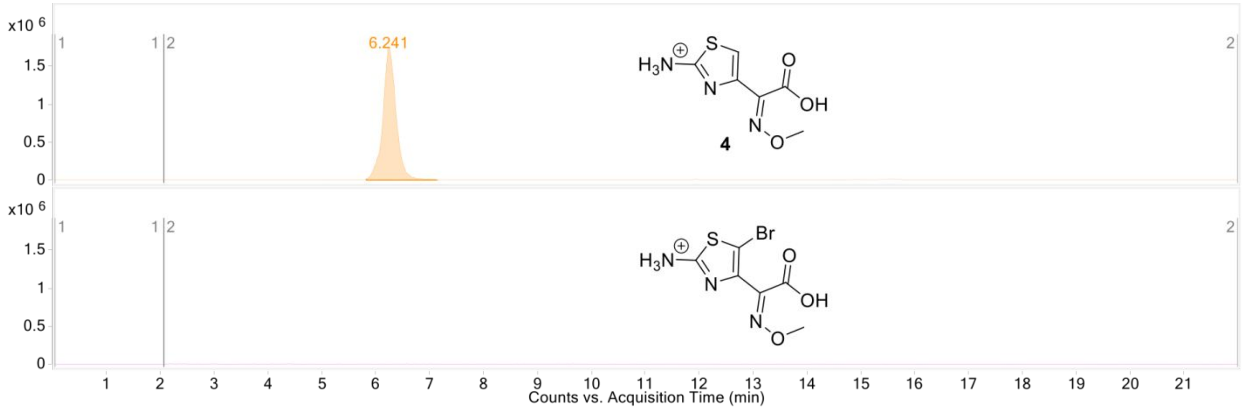

Figure S18: [M+H]$^{1+}$ EICs demonstrating the presence of substrate **4** (top panel) and mono-brominated product (bottom panel) detected to be produced in a negative control enzymatic reaction wherein the catalyst CcVHPO1 was omitted from the reaction.

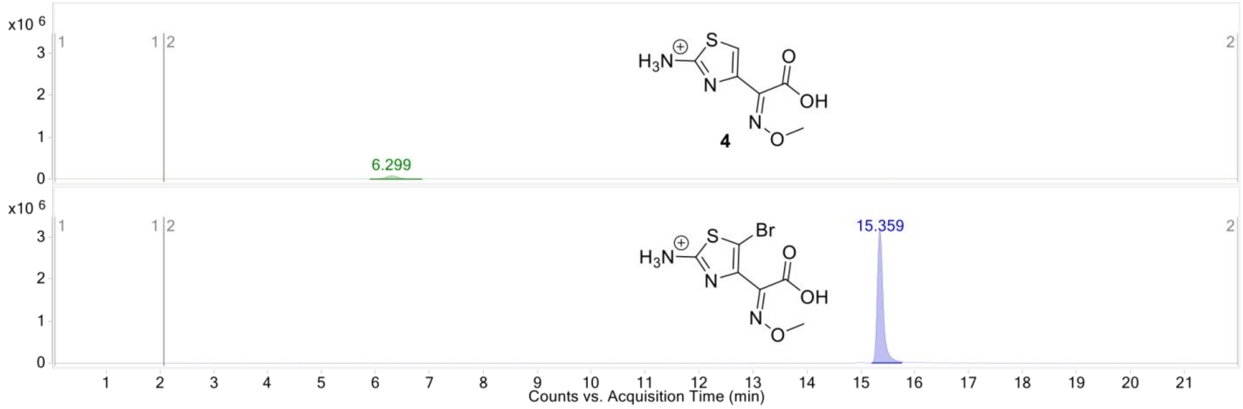

Figure S19: [M+H]$^{1+}$ EICs demonstrating the presence of substrate **4** (top panel) and monobrominated product (bottom panel) detected to be produced in an enzymatic reaction using CcVHPO1. The brominated product has a higher retention time than the substrate.

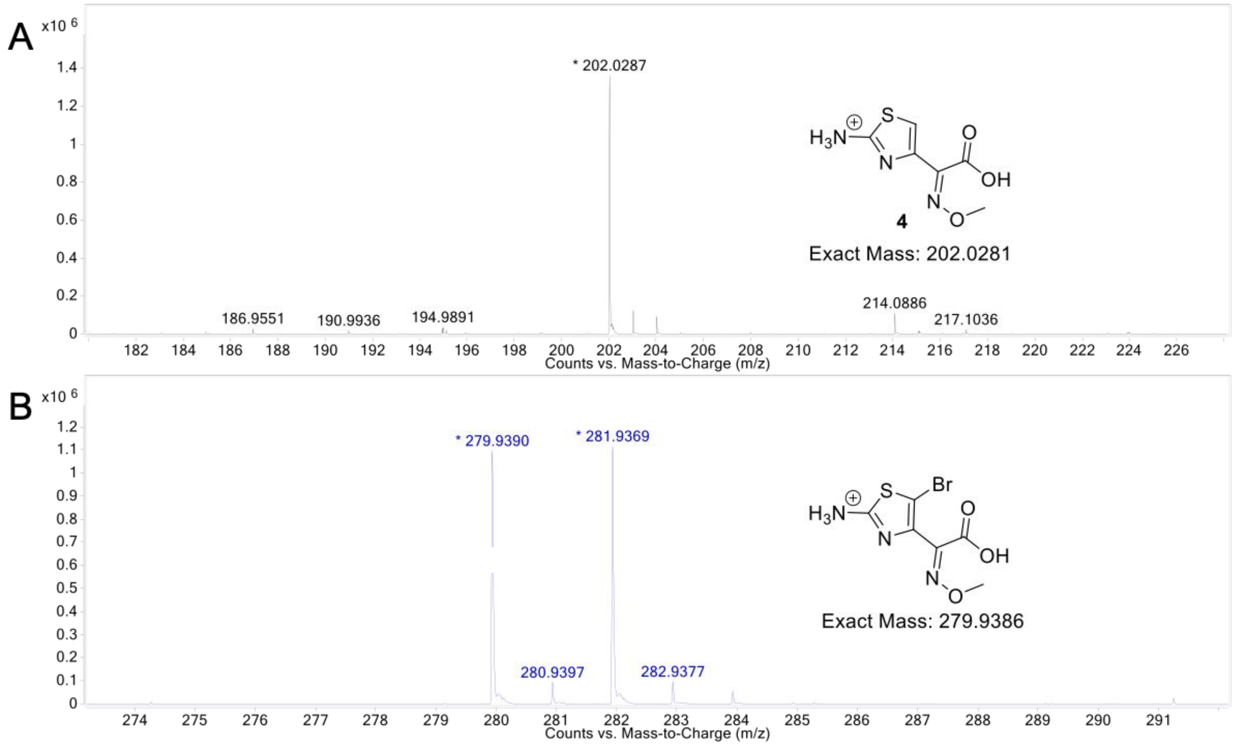

Figure S20: (**A**) [M+H]$^{1+}$ MS^1 spectra of **4**. (**B**) [M+H]$^{1+}$ MS^1 spectra for mono brominated **4**.

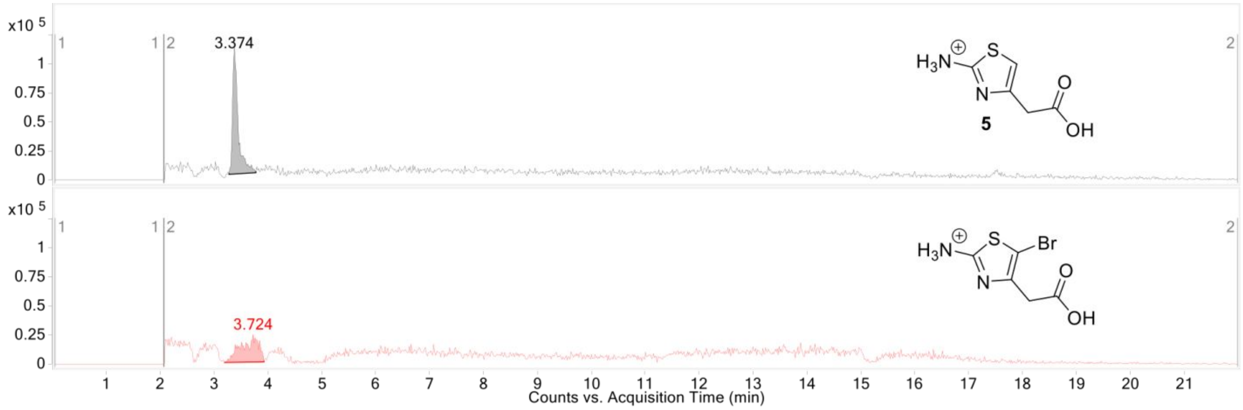

Figure S21: $[M+H]^{1+}$ EICs demonstrating presence of substrate **5** (top panel) and monobrominated product (bottom panel) detected to be produced in a negative control enzymatic reaction wherein the catalyst CcVHPO1 was omitted from the reaction.

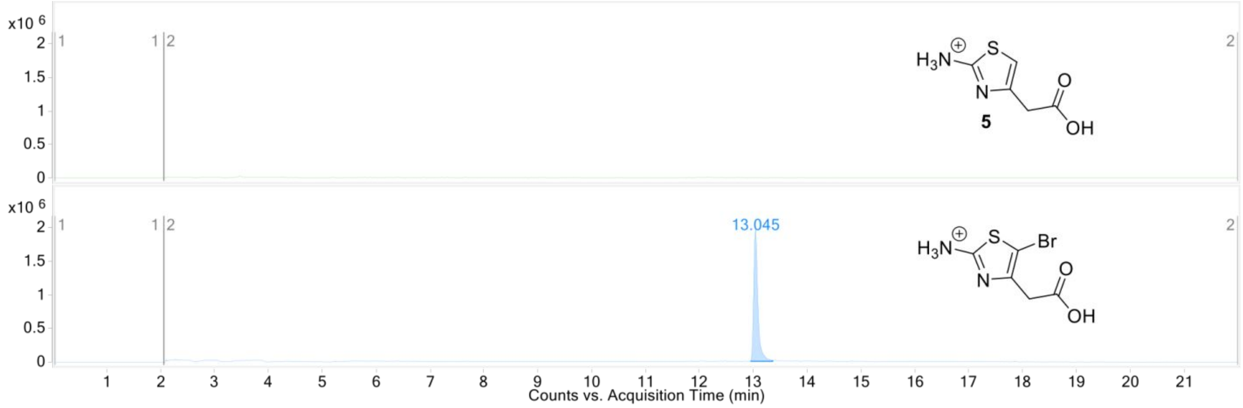

Figure S22: $[M+H]^{1+}$ EICs demonstrating the presence of substrate 5 (top panel) and mono-brominated product (bottom panel) detected to be produced in an enzymatic reaction using CcVHPO1. The brominated product has a higher retention time than the substrate.

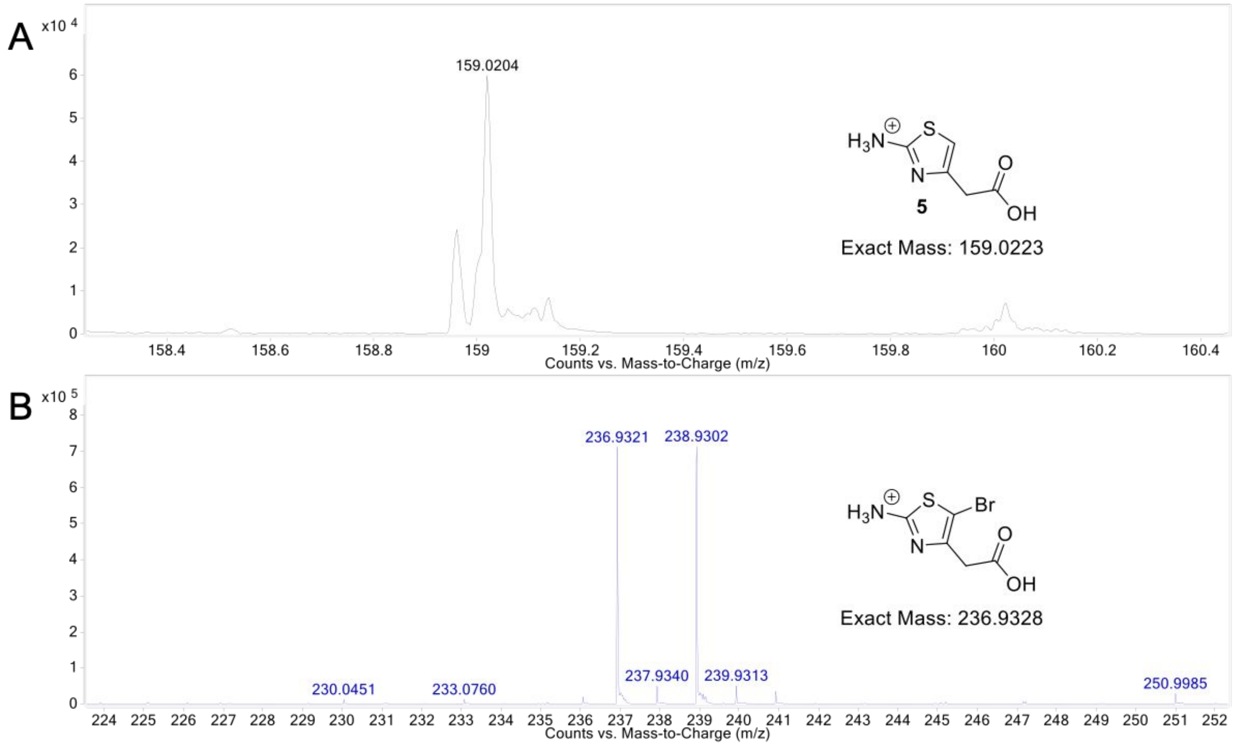

Figure S23: (**A**) $[M+H]^{1+}$ MS^1 spectra of **5**. (**B**) $[M+H]^{1+}$ MS^1 spectra for mono brominated **5**.

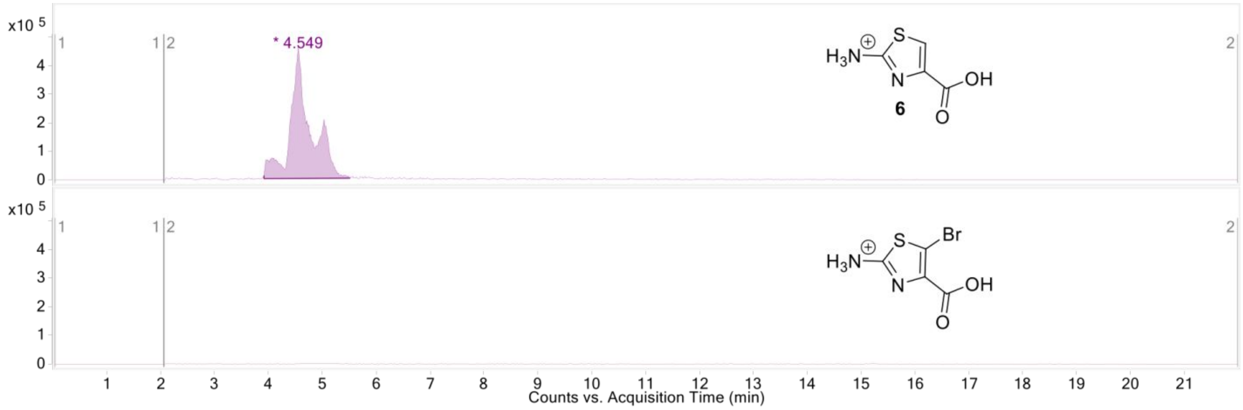

Figure S24: $[M+H]^{1+}$ EICs demonstrating presence of substrate **6** (top panel) and monobrominated product (bottom panel) detected to be produced in a negative control enzymatic reaction wherein the catalyst CcVHPO1 was omitted from the reaction. The zwitterionic and highly polar molecule **6** consistently demonstrated poor chromatography profiles.

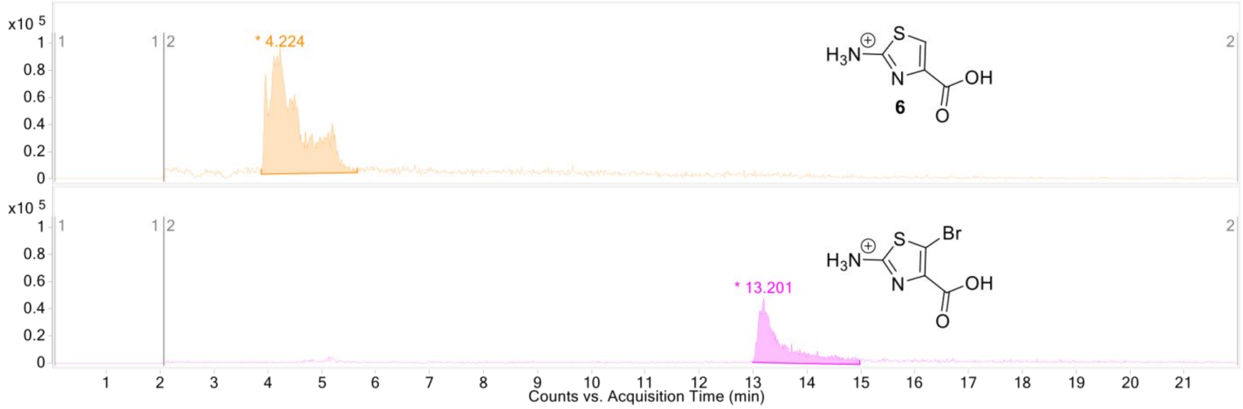

Figure S25: $[M+H]^{1+}$ EICs demonstrating the presence of substrate 6 (top panel) and mono-brominated product (bottom panel) detected to be produced in an enzymatic reaction using CcVHPO1. The brominated product has a higher retention time than the substrate.

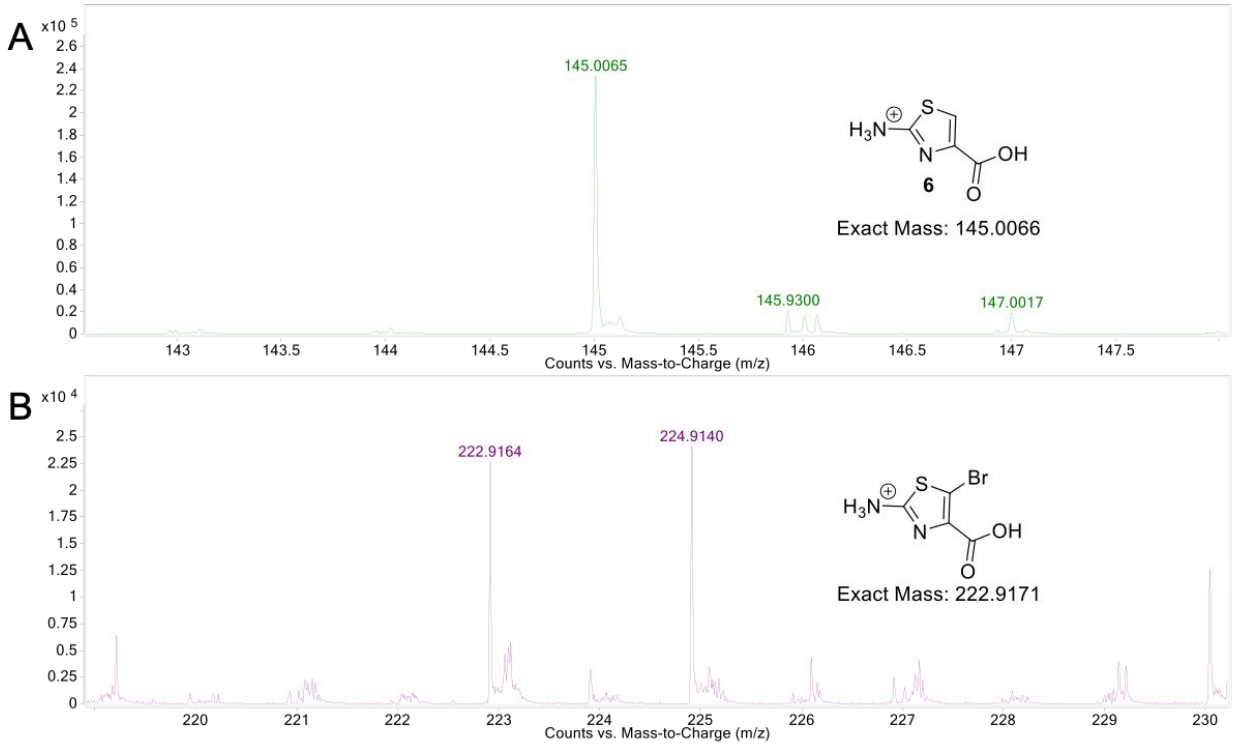

Figure S26: (**A**) [M+H]$^{1+}$ MS^1 spectra of **6**. (**B**) [M+H]$^{1+}$ MS^1 spectra for monobrominated **6**.

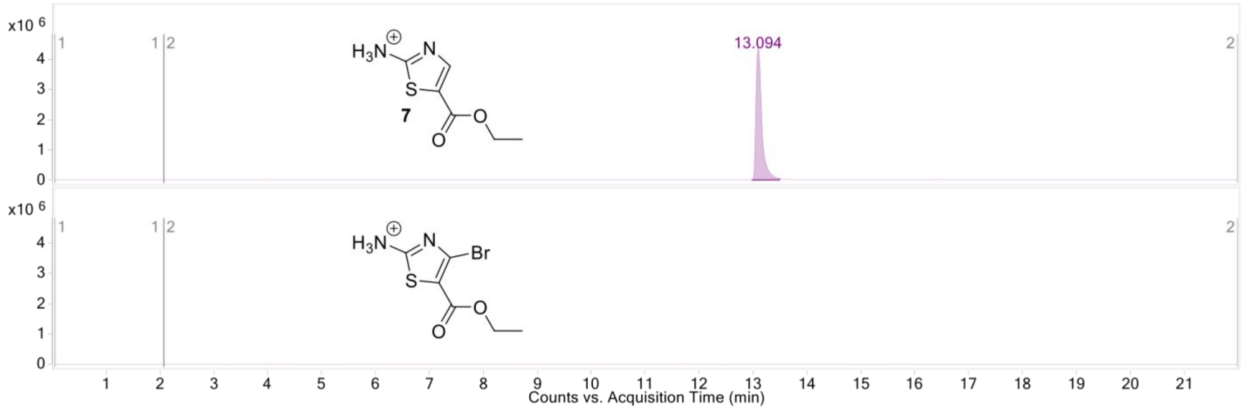

Figure S27: [M+H]$^{1+}$ EICs demonstrating presence of substrate **7** (top panel) and monobrominated product (bottom panel) detected to be produced in a negative control enzymatic reaction wherein the catalyst CcVHPO1 was omitted from the reaction.

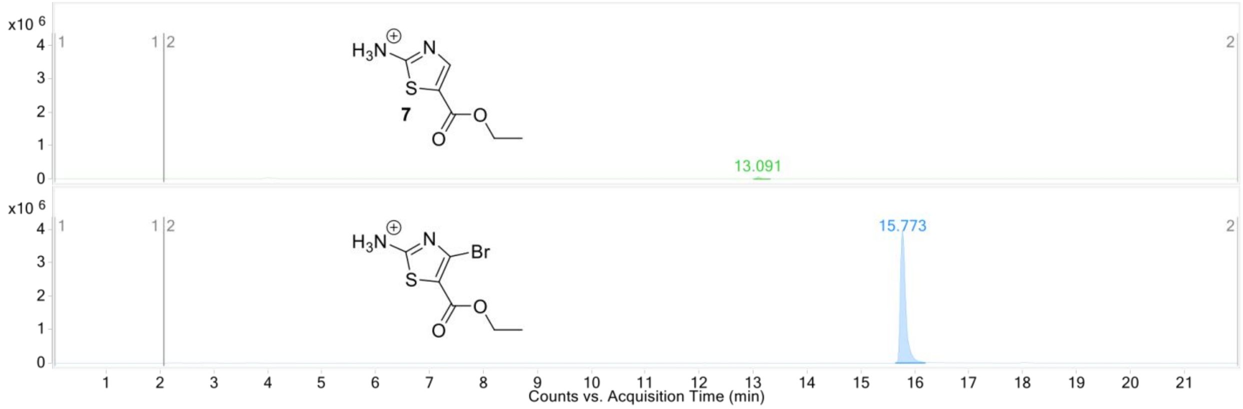

Figure S28: [M+H]$^{1+}$ EICs demonstrating presence of substrate **7** (top panel) and monobrominated product (bottom panel) detected to be produced in an enzymatic reaction using CcVHPO1. The brominated product has a higher retention time as compared to the substrate.

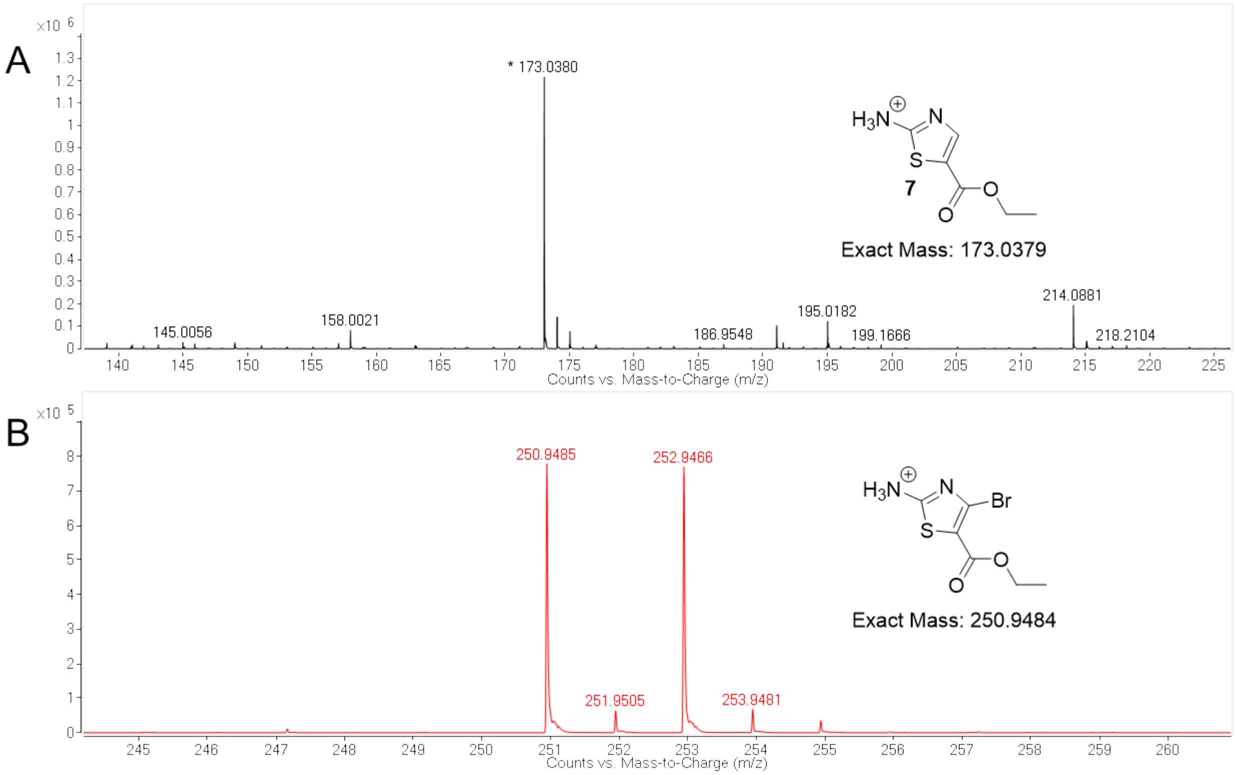

Figure S29: (**A**) $[M+H]^{1+}$ MS^1 spectra of **7**. (**B**) $[M+H]^{1+}$ MS^1 spectra for mono brominated **7**.

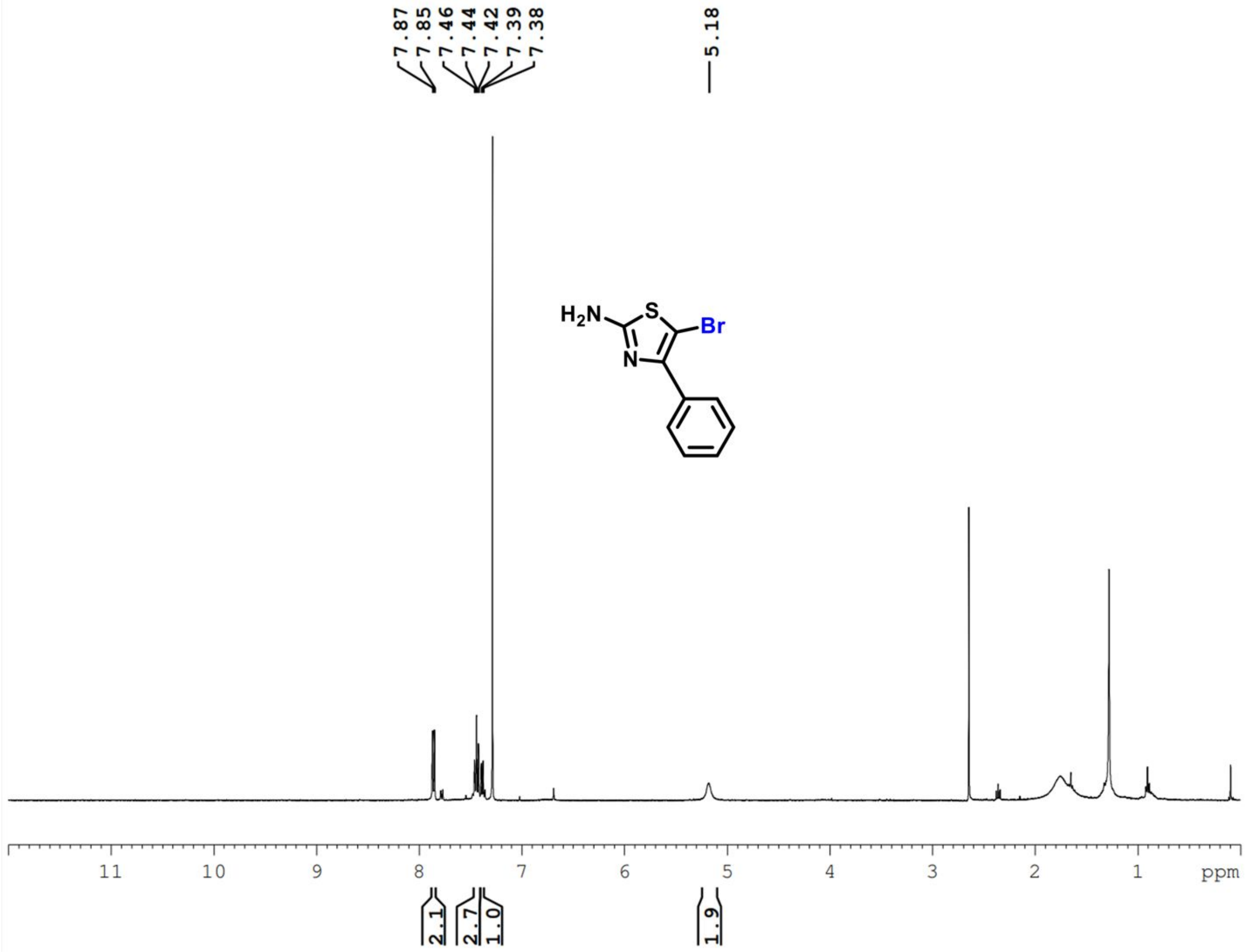

Figure S30: ^{1}H-NMR spectrum ($CDCl_3$, 400 MHz) of **3-Br**.

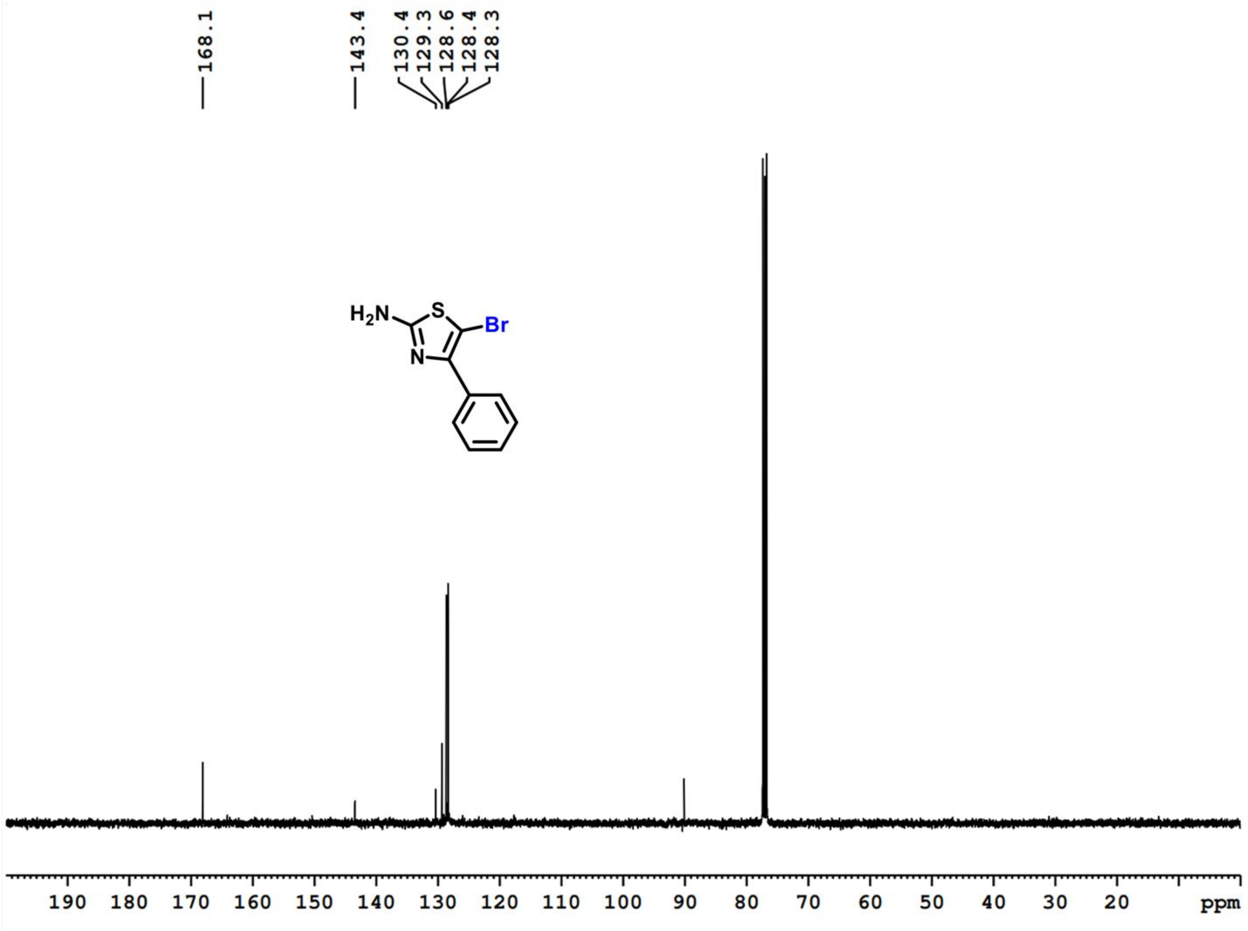

Figure S31: $^{13}C\{^{1}H\}$ NMR spectrum ($CDCl_3$, 125 MHz) of **3-Br**.

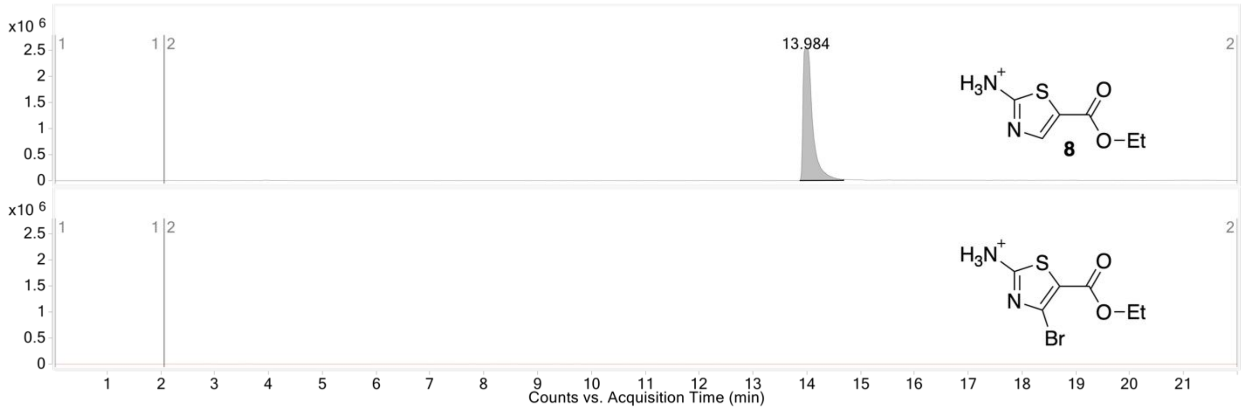

Figure S32: [M+H]$^{1+}$ EICs demonstrating presence of substrate **8** (top panel) and monobrominated product (bottom panel) detected to be produced in a negative control enzymatic reaction wherein the catalyst CcVHPO1 was omitted from the reaction.

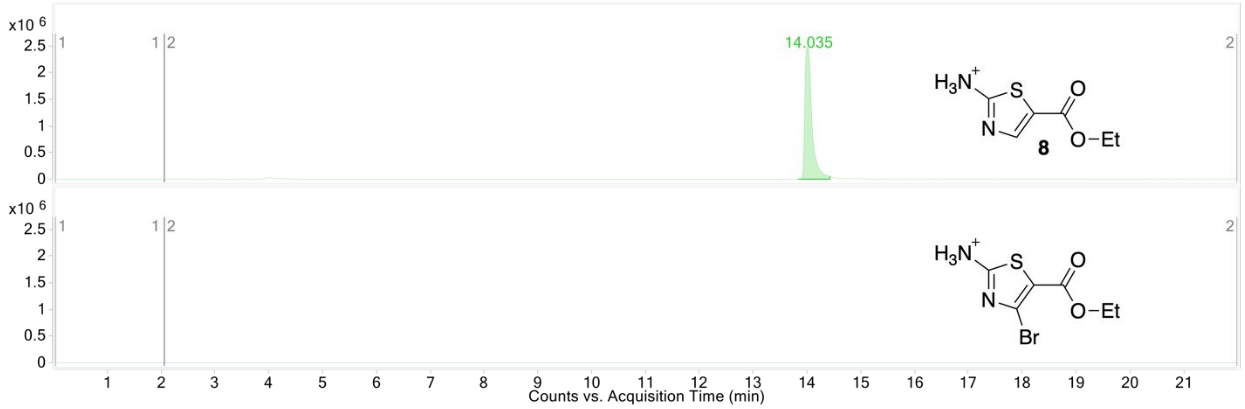

Figure S33: [M+H]$^{1+}$ EICs demonstrating the presence of substrate 8 (top panel) and mono-brominated product (bottom panel) detected to be produced in an enzymatic reaction using CcVHPO1. Note that no brominated product formation is observed.

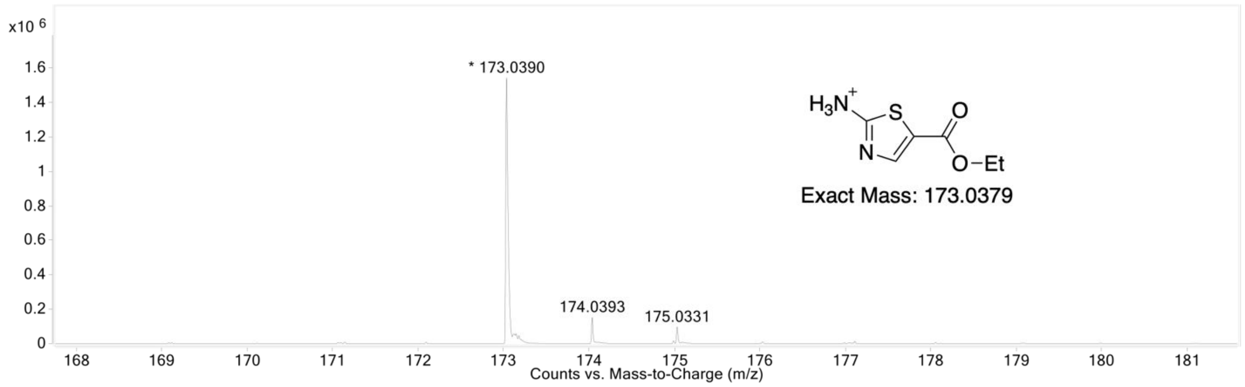

Figure S34: $[M+H]^{1+}$ MS^1 spectra of **8**. No brominated product formation for **8** was observed.

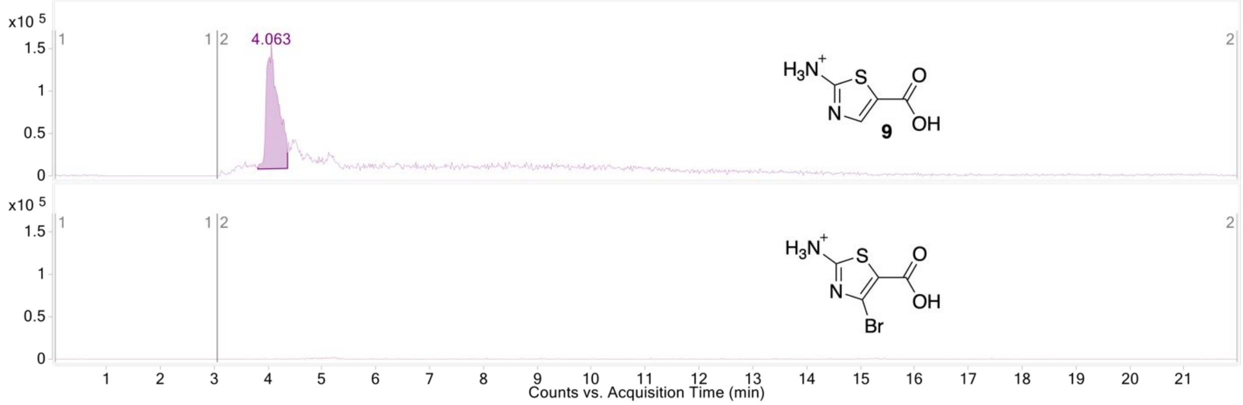

Figure S35: $[M+H]^{1+}$ EICs demonstrating the presence of substrate **9** (top panel) and mono brominated product (bottom panel) detected to be produced in a negative control enzymatic reaction wherein the catalyst CcVHPO1 was omitted from the reaction. As for molecule **6**, molecule **9** also demonstrated poor chromatography profiles.

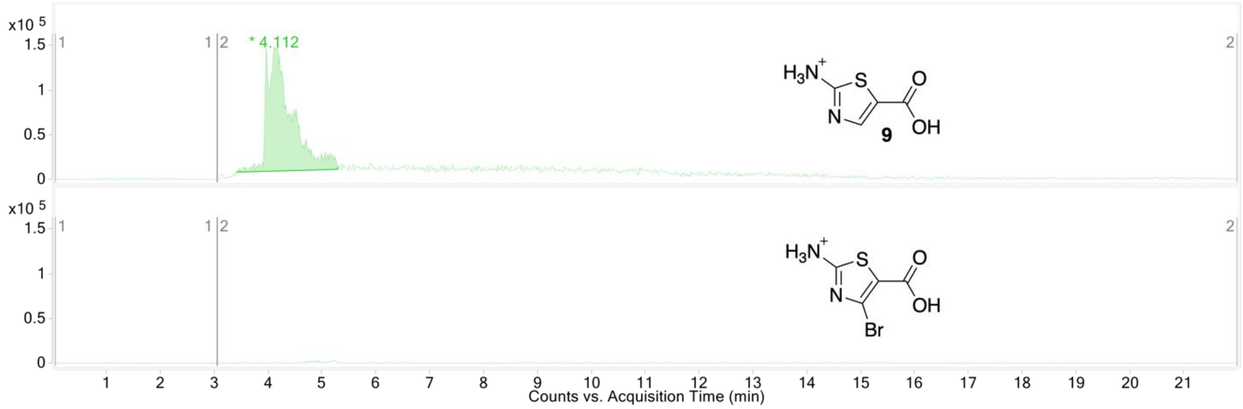

Figure S36: $[M+H]^{1+}$ EICs demonstrating the presence of substrate **9** (top panel) and mono-brominated product (bottom panel) detected to be produced in an enzymatic reaction using CcVHPO1. Note that no brominated product formation is observed.

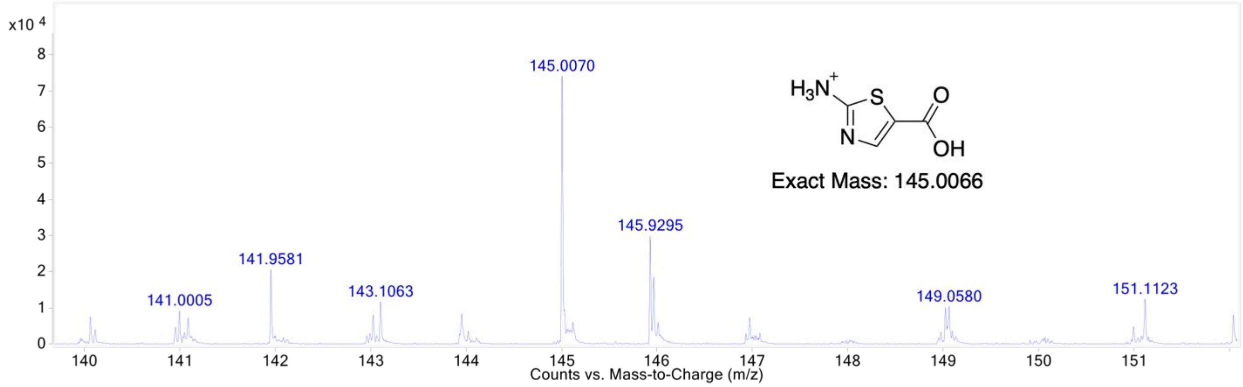

Figure S37: [M+H]$^{1+}$ MS^1 spectra of **9**. No brominated product formation for **9** was observed.

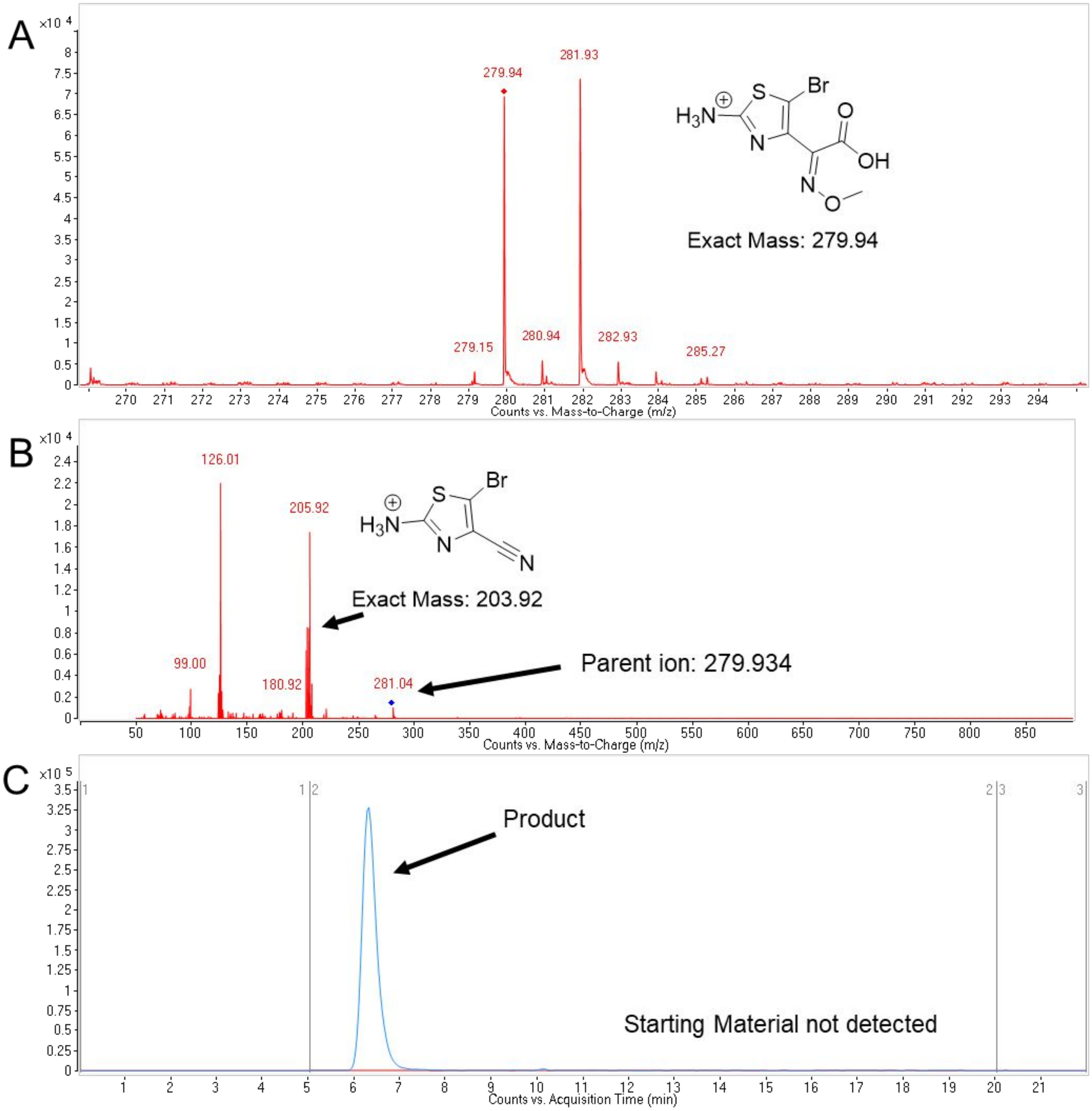

Figure S38: (**A**) Isotopic distribution of the brominated product starting from substrate molecule **4**. (**B**) MS^2 fragmentation spectra for the brominated product with a key product ion structurally annotated. This product ion demonstrates that the bromination did not alter the oxime functionality. (**C**) EICs monitoring product formation and unconverted substrate in the bromination reaction in blue and red, respectively. The EICs were generated using the most abundant $[M+H]^{1+}$ ions.

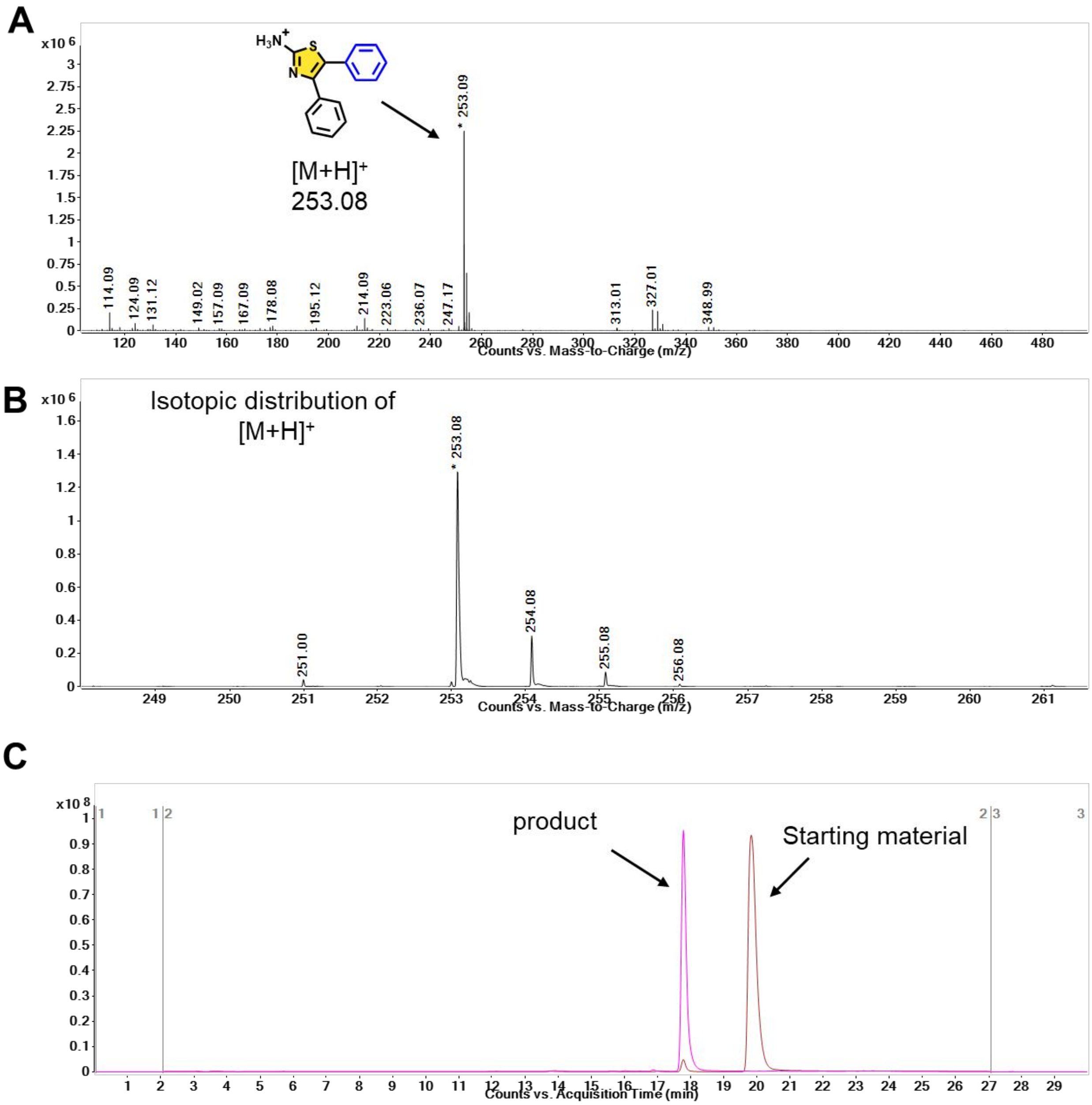

Figure S39: (**A**) MS[1] spectra for the SMCC product 4,5-biphenyl-2-aminothiazole. (**B**) Isotopic distribution of the product ($[M+H]^{1+}$ ions). (**C**) EICs monitoring product formation and unconverted substrate in SMCC reaction in pink and brown, respectively. The EICs were generated using the most abundant $[M+H]^{1+}$ ions.

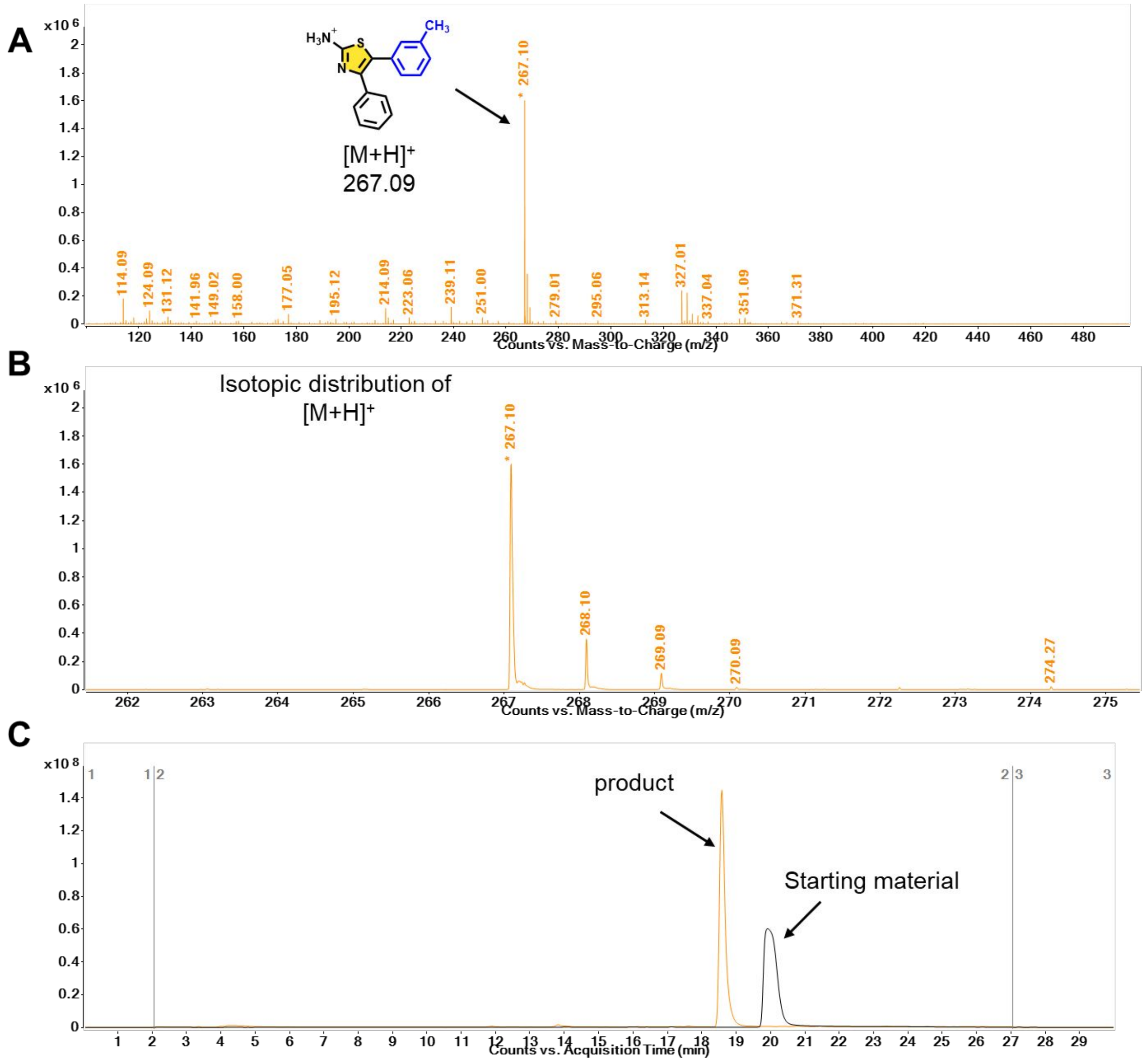

Figure S40: (**A**) MS[1] spectra for the SMCC product 5-(3-methylphenyl)-4-phenyl-2-aminothiazole. (**B**) Isotopic distribution of the product ($[M+H]^{1+}$ ions). **(C)** EICs monitor product formation and unconverted substrate (5-bromo-4-phenyl-2-aminothiazole) in SMCC reactions in orange and black, respectively. The EICs were generated using the most abundant $[M+H]^+$ ions.

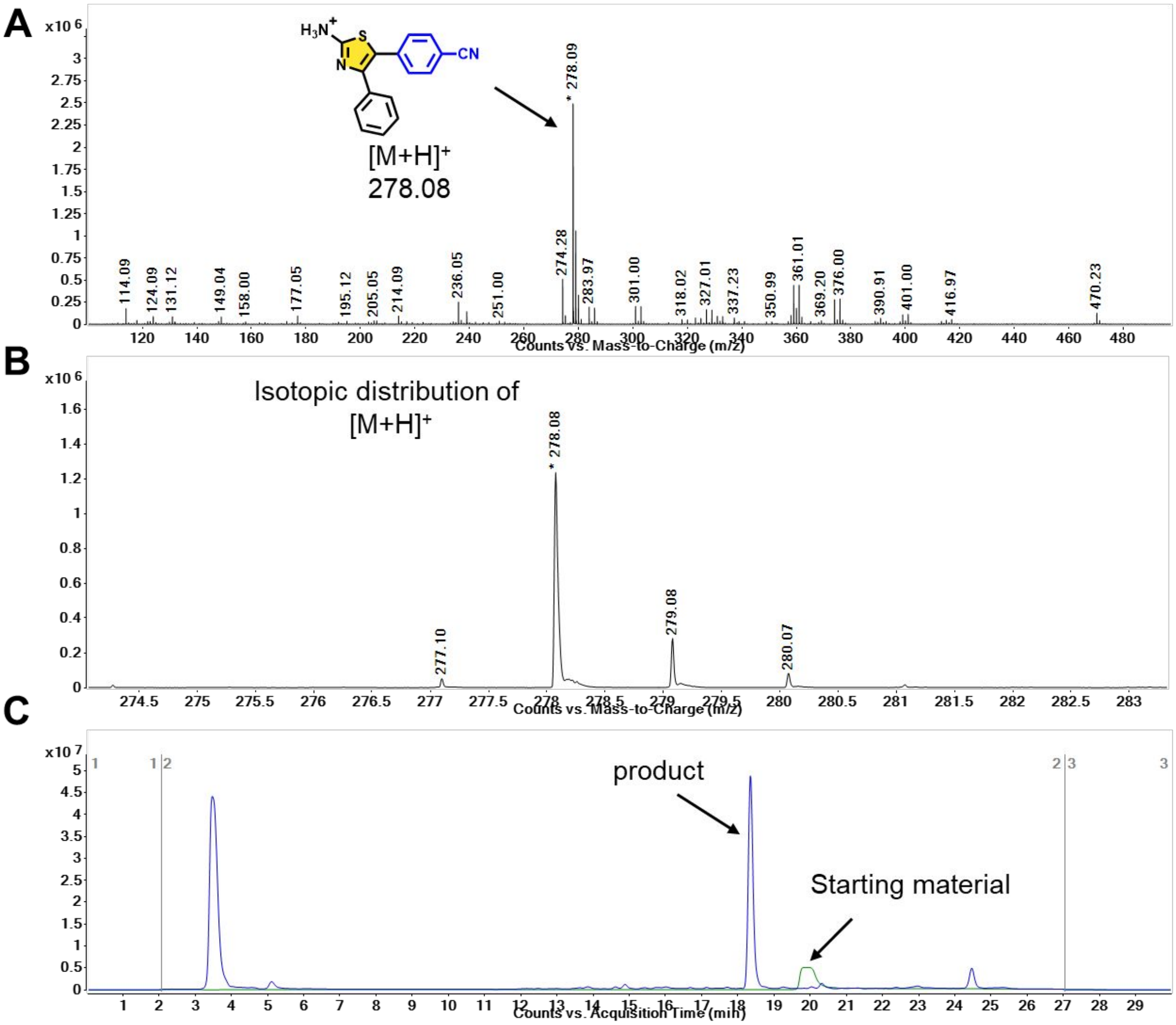

Figure S41: (**A**) MS[1] spectra for the SMCC product 5-(4-cyanophenyl)-4-phenyl-2-aminothiazole. (**B**) Isotopic distribution of the product ($[M+H]^{1+}$ ions). **(C)** EICs monitoring product formation and unconverted substrate in SMCC reaction in blue and green, respectively. The EICs were generated using the most abundant $[M+H]^{1+}$ ions.

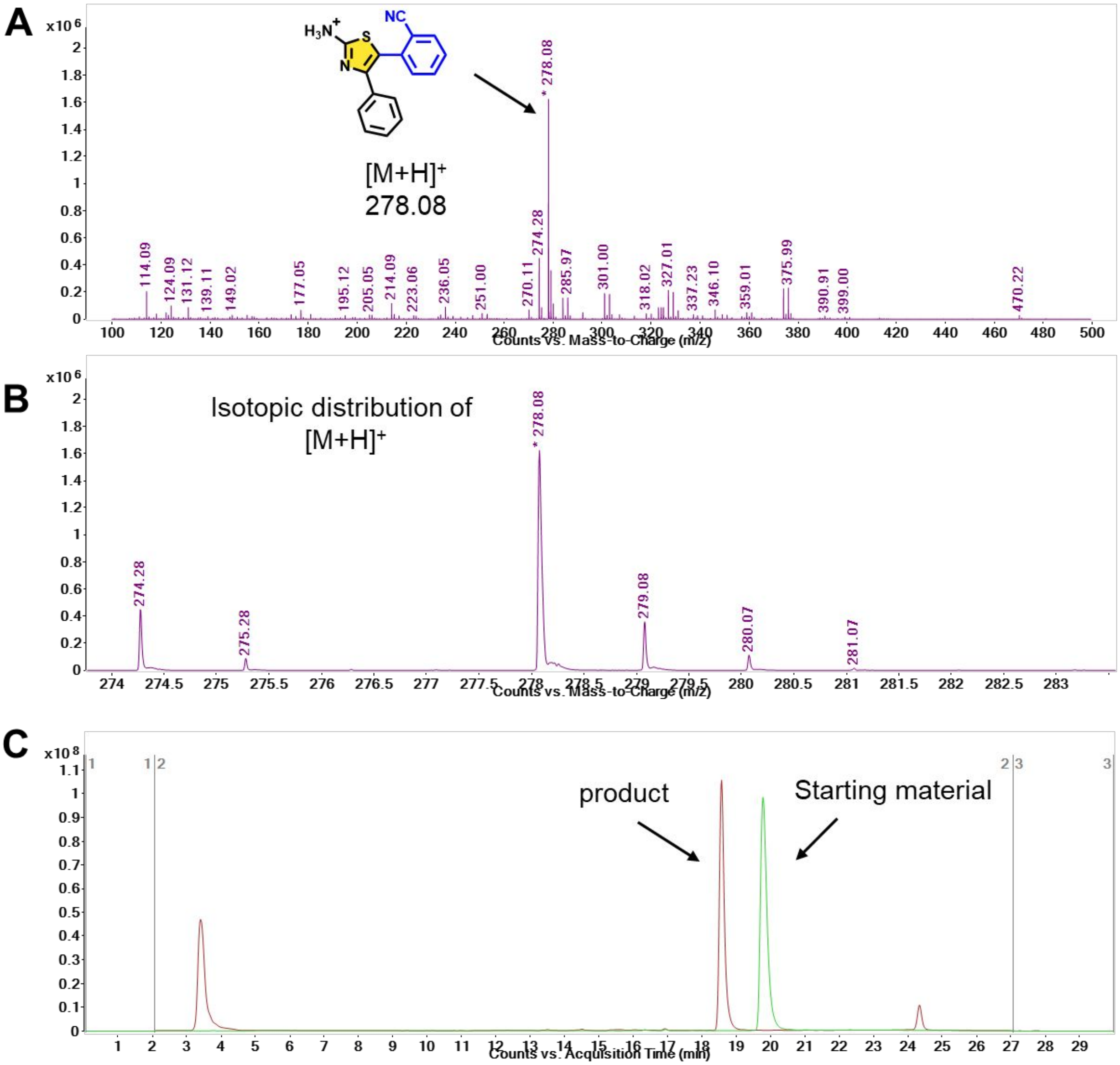

Figure S42: (**A**) MS[1] spectra for the SMCC product 5-(2-cyanophenyl)-4-phenyl-2-aminothiazole. (**B**) Isotopic distribution of the product ($[M+H]^{1+}$ ions). **(C)** EICs monitoring product formation and unconverted substrate in SMCC reaction in brown and green, respectively. The EICs were generated using the most abundant $[M+H]^+$ ions.

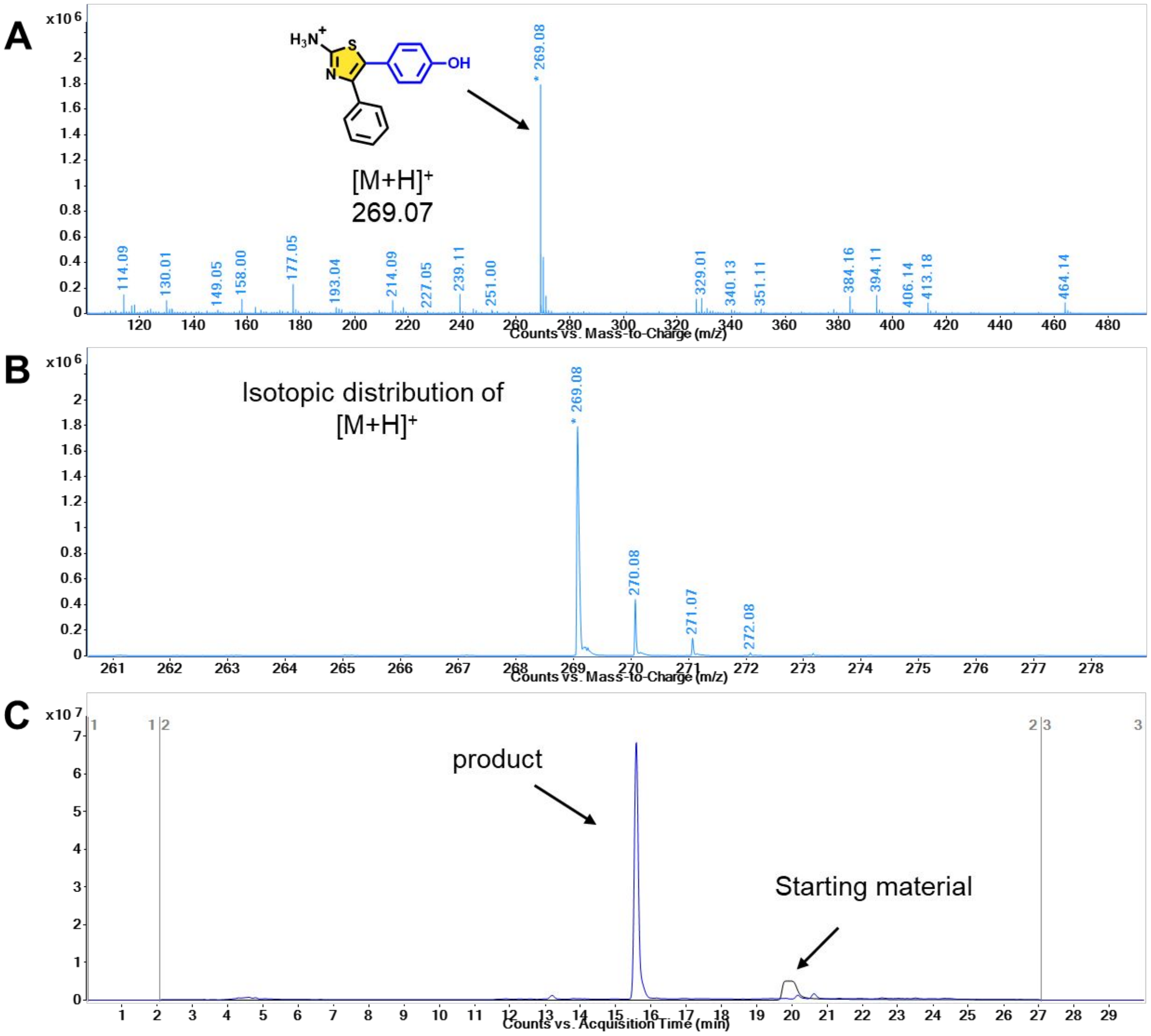

Figure S43: (**A**) MS[1] spectra for the SMCC product 5-(4-hydroxyphenyl)-4-phenyl-2-aminothiazole. (**B**) Isotopic distribution of the product ($[M+H]^{1+}$ ions). **(C)** EICs monitoring product formation and unconverted substrate in SMCC reaction in blue and black, respectively. The EICs were generated using the most abundant $[M+H]^{1+}$ ions.

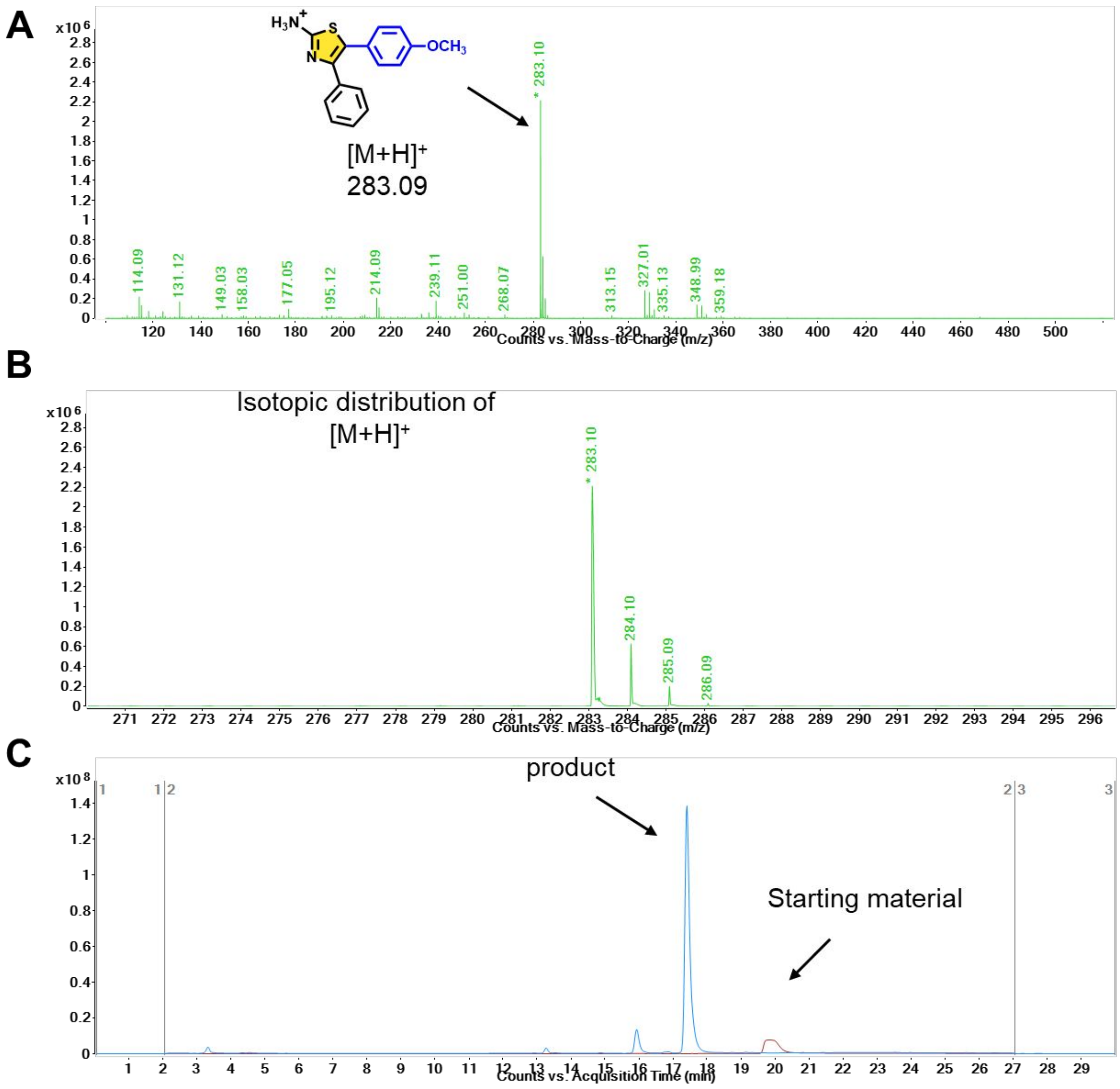

Figure S44: (**A**) MS[1] spectra for the SMCC product 5-(4-methoxyphenyl)-4-phenyl-2-aminothiazole. (**B**) Isotopic distribution of the product ($[M+H]^{1+}$ ions). **(C)** EICs monitoring product formation and unconverted substrate in SMCC reaction in cyan and brown, respectively. The EICs were generated using the most abundant $[M+H]^{1+}$ ions.

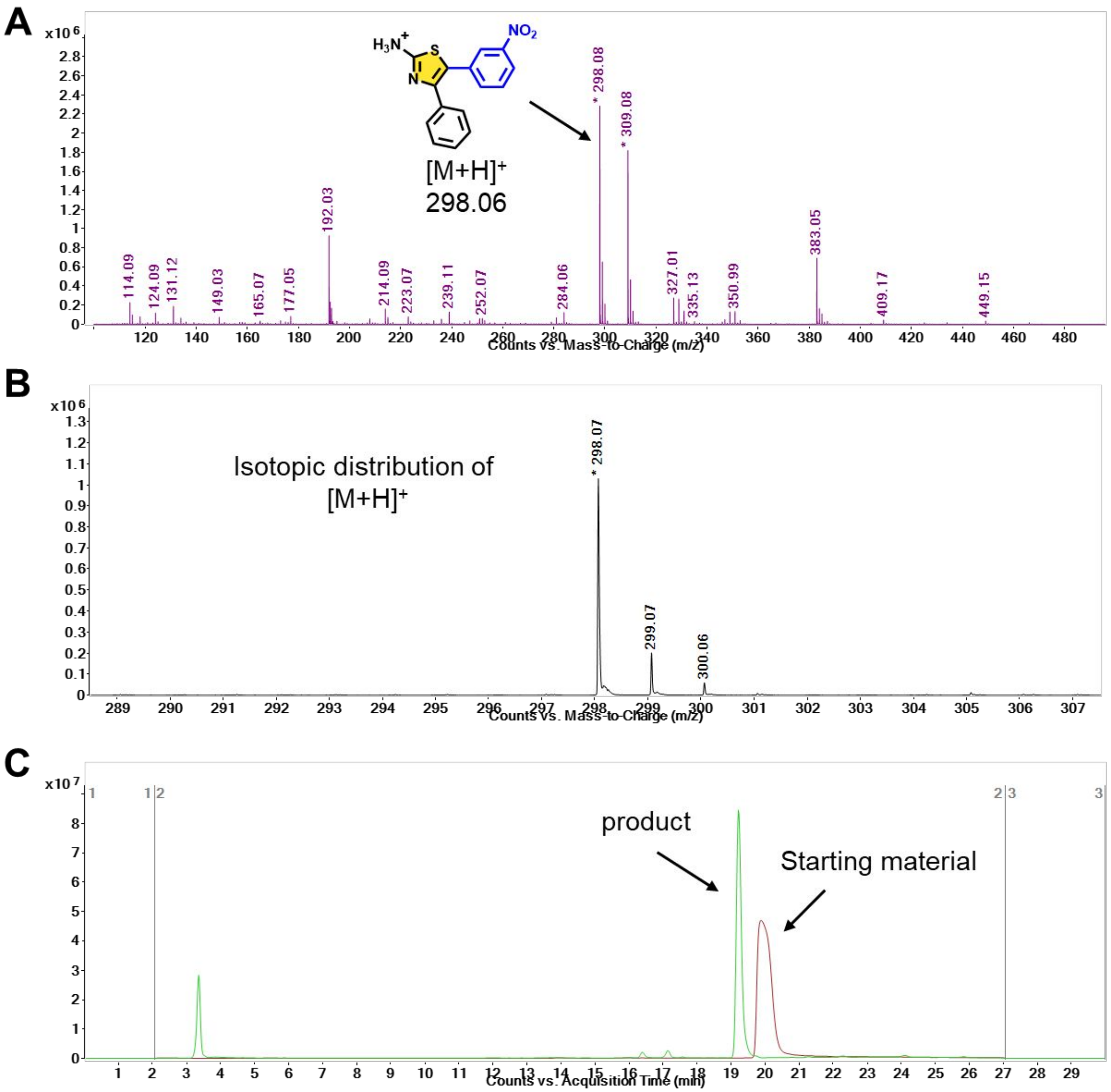

Figure S45: (**A**) MS[1] spectra for the SMCC product 5-(3-nitrophenyl)-4-phenyl-2-aminothiazole. (**B**) Isotopic distribution of the product ($[M+H]^{1+}$ ions). **(C)** EICs monitoring product formation and unconverted substrate in SMCC reaction in green and brown, respectively. The EICs were generated using the most abundant $[M+H]^{1+}$ ions.

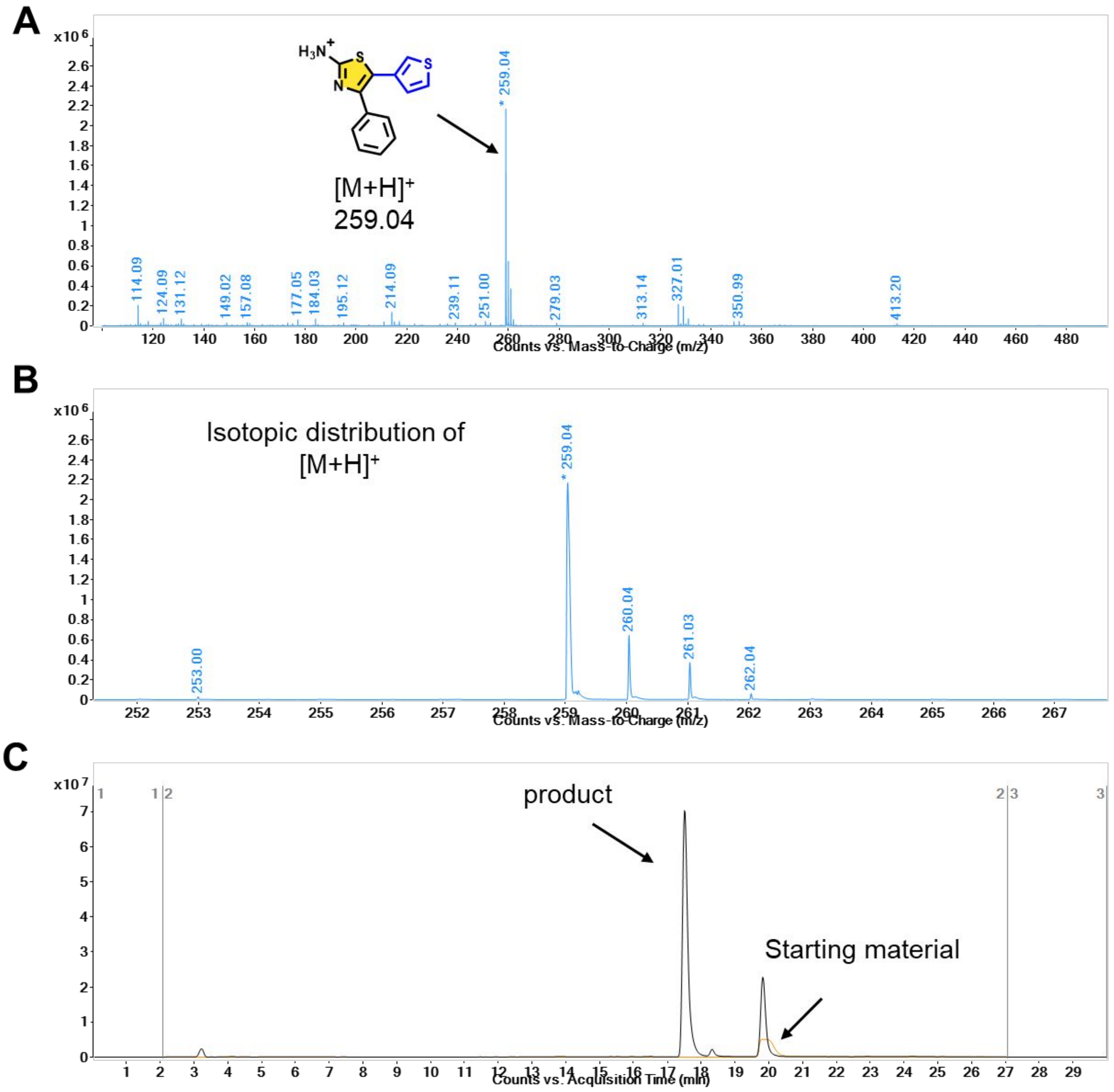

Figure S46: (**A**) MS[1] spectra for the SMCC product 5-(thiophen-3-yl)-4-phenyl-2-aminothiazole. (**B**) Isotopic distribution of the product ($[M+H]^{1+}$ ions). **(C)** EICs monitoring product formation and unconverted substrate in SMCC reaction in black and orange, respectively. The EICs were generated using the most abundant $[M+H]^{1+}$ ions.

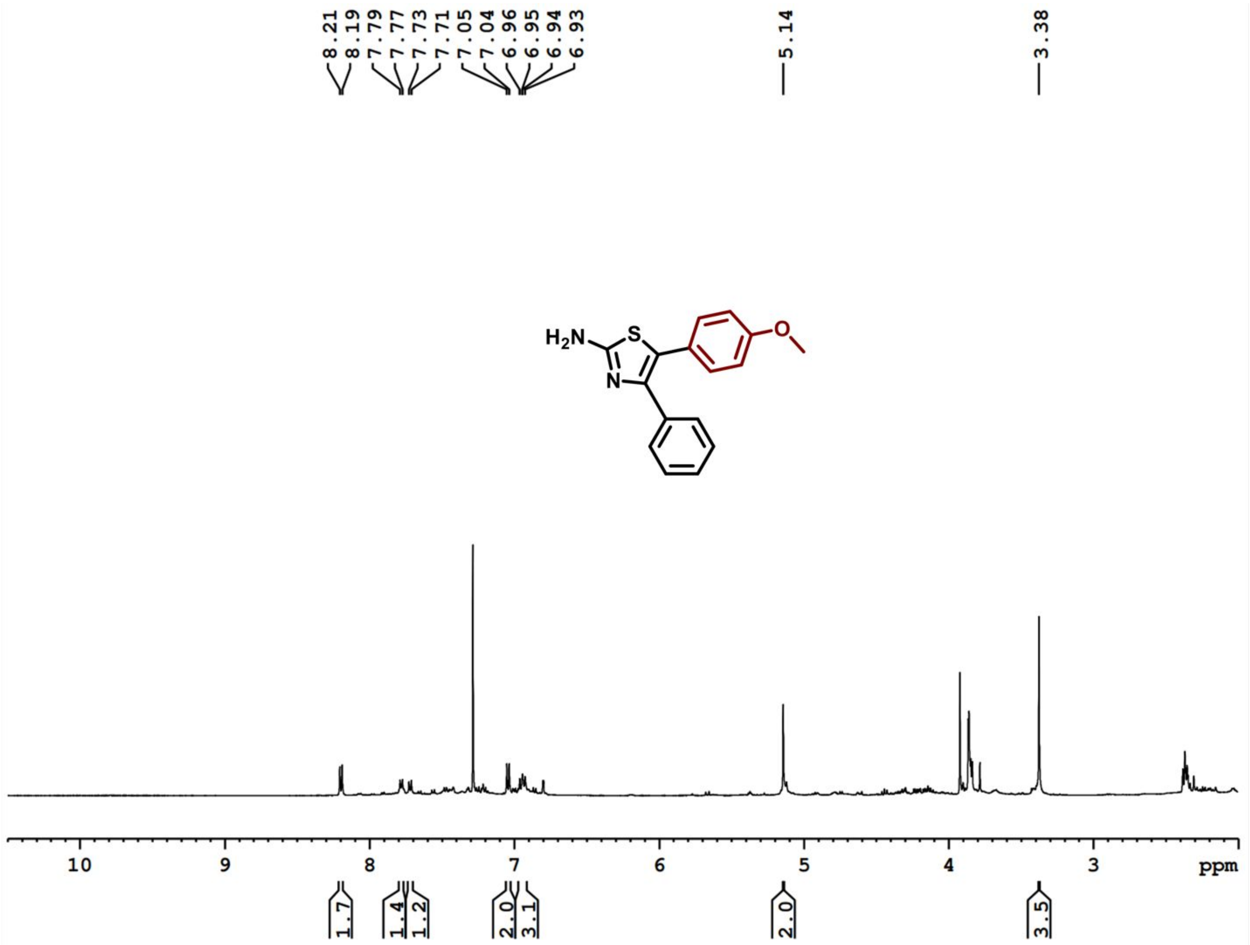

Figure S47: ^{1}H-NMR spectrum ($CDCl_3$, 500 MHz) of Suzuki-Miyaura cross coupling product 2-amino-4-phenyl-5-(*p*-methoxyphenyl)thiazole.

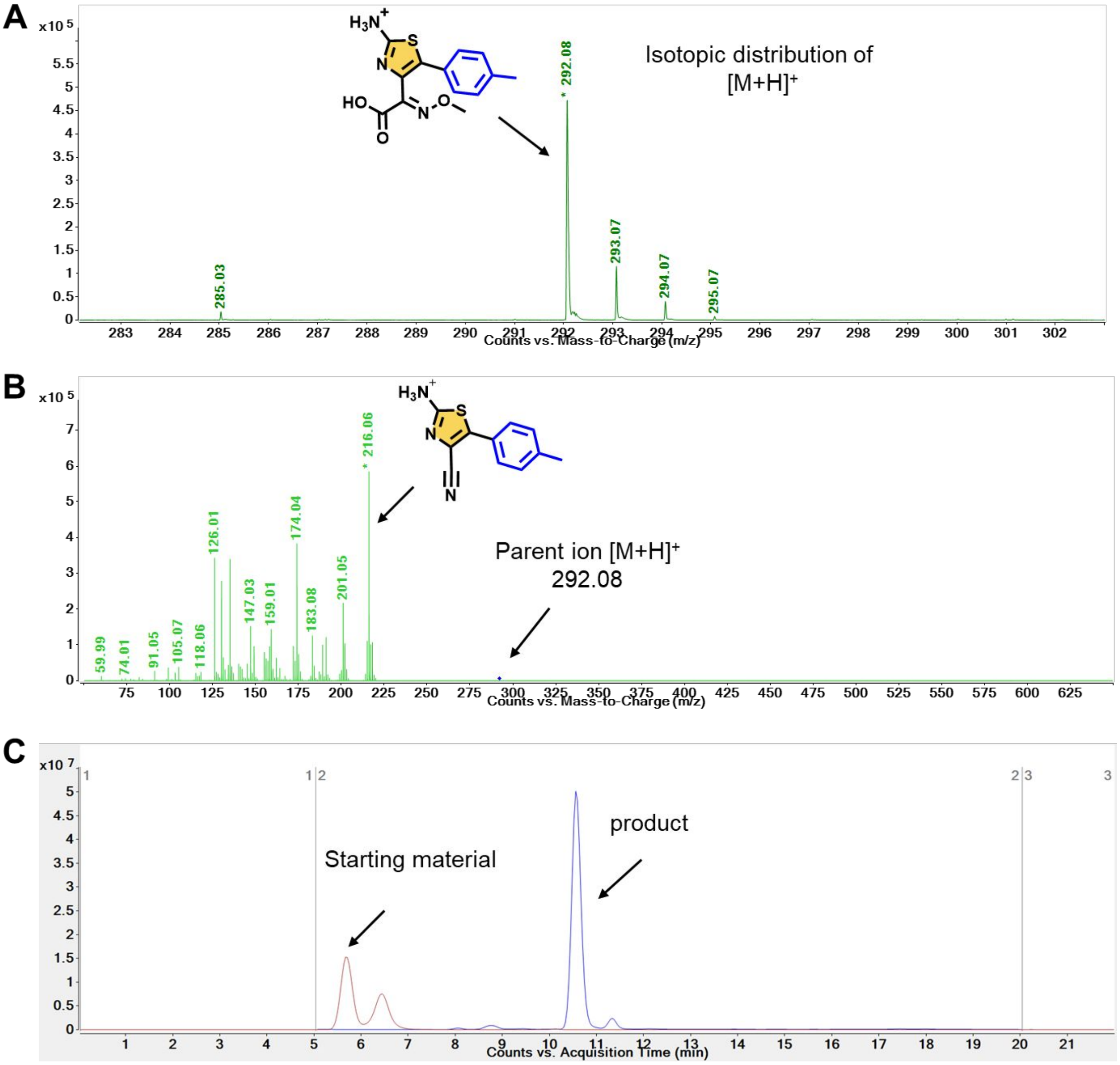

Figure S48: (**A**) Isotopic distribution of the SMCC product wherein a tolyl group has been added to the 5-position of substrate molecule **4**. (**B**) MS^2 spectra for the SMCC product with a key product ion structurally annotated. **(C)** EICs monitoring product formation and unconverted substrate in the SMCC reaction in blue and brown, respectively. The EICs were generated using the most abundant $[M+H]^+$ ions.

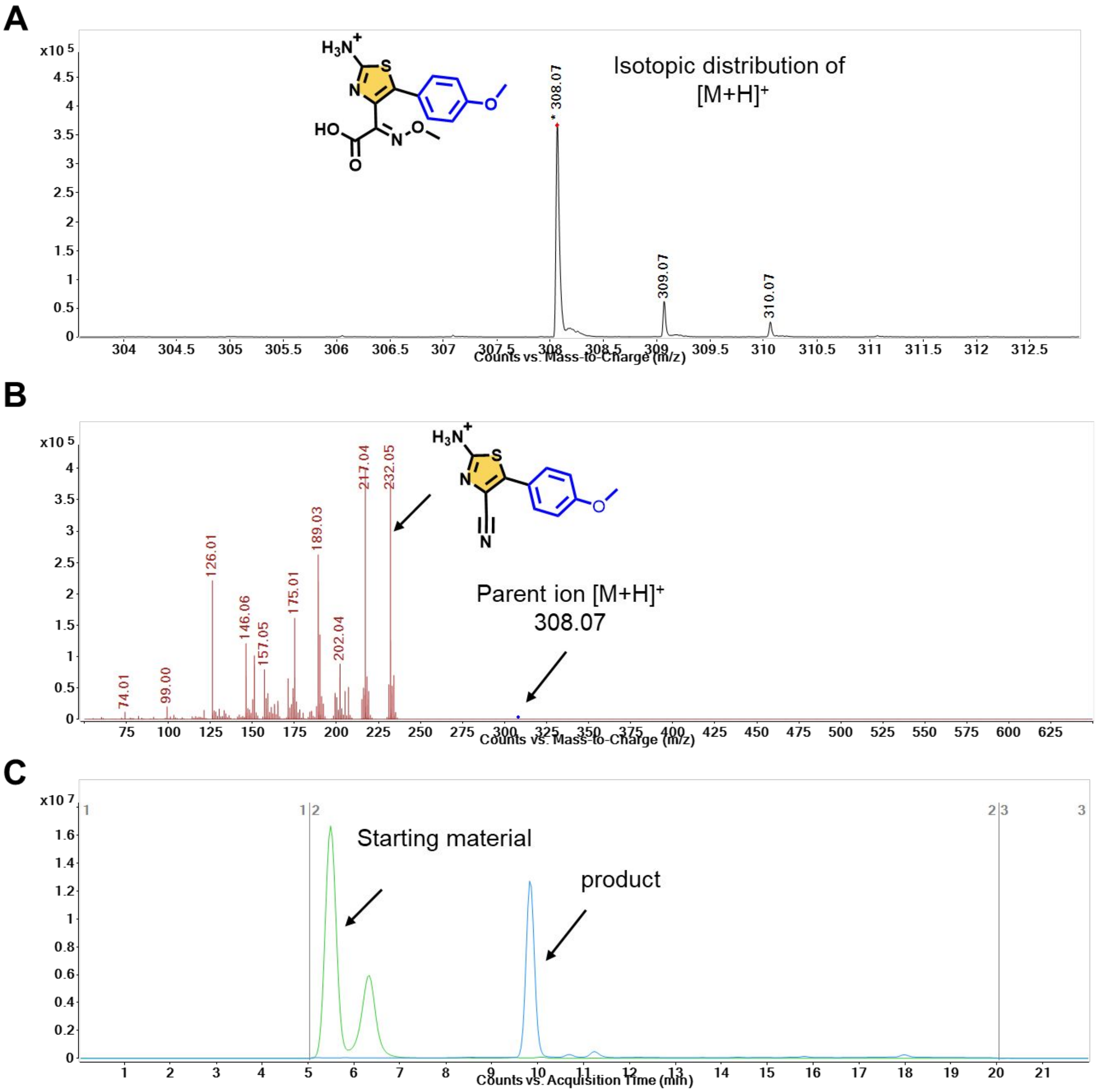

Figure S49: (**A**) Isotopic distribution of the SMCC product wherein a *p*-methoxyphenyl group has been added to the 5-position of substrate molecule **4**. (**B**) MS^2 spectra for the SMCC product with a key product ion structurally annotated. **(C)** EICs monitoring product formation and unconverted substrate in the SMCC reaction in cyan and green, respectively. The EICs were generated using the most abundant $[M+H]^+$ ions.

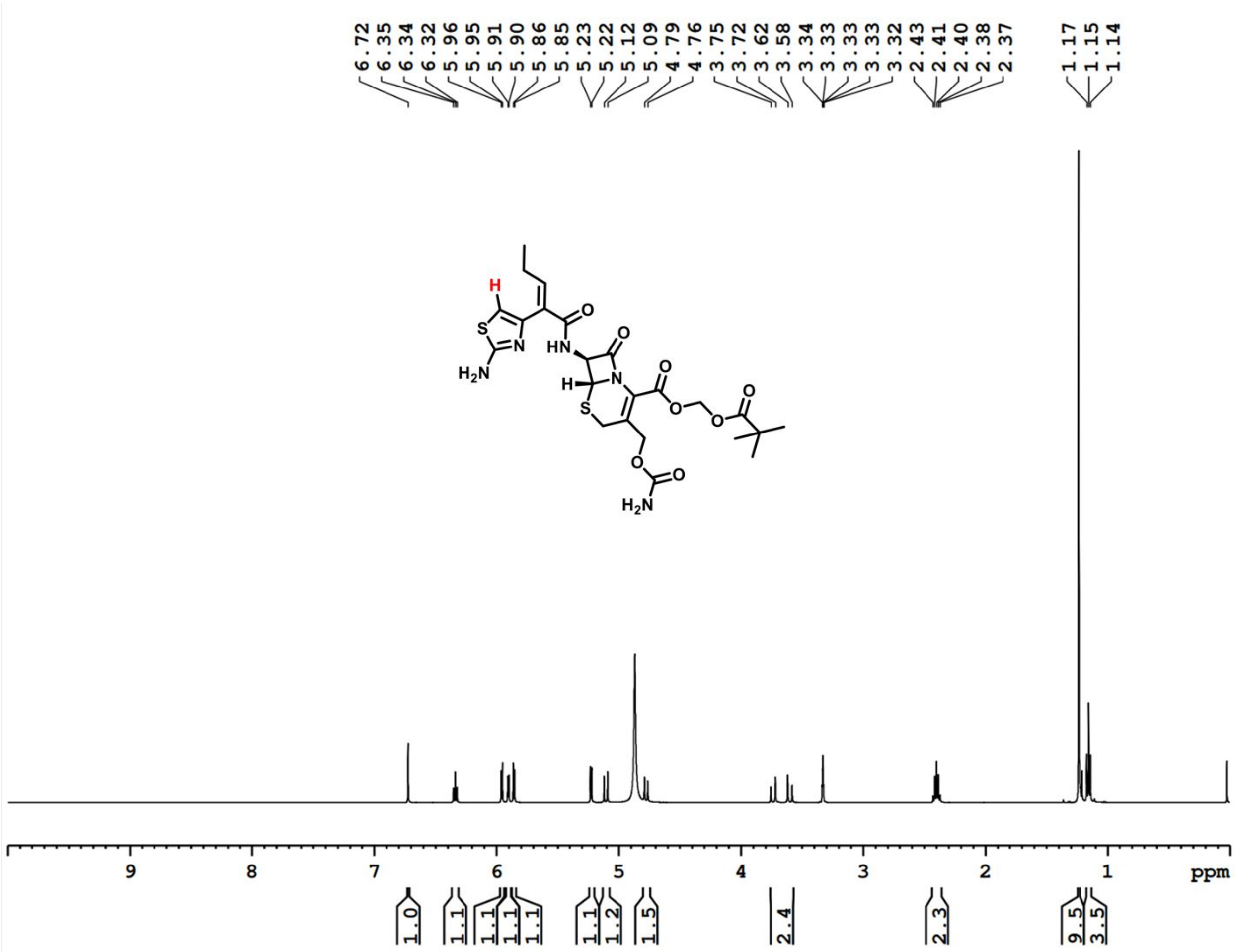

Figure S50: ^{1}H-NMR spectrum (Methanol-d_4, 500 MHz) of **12**.

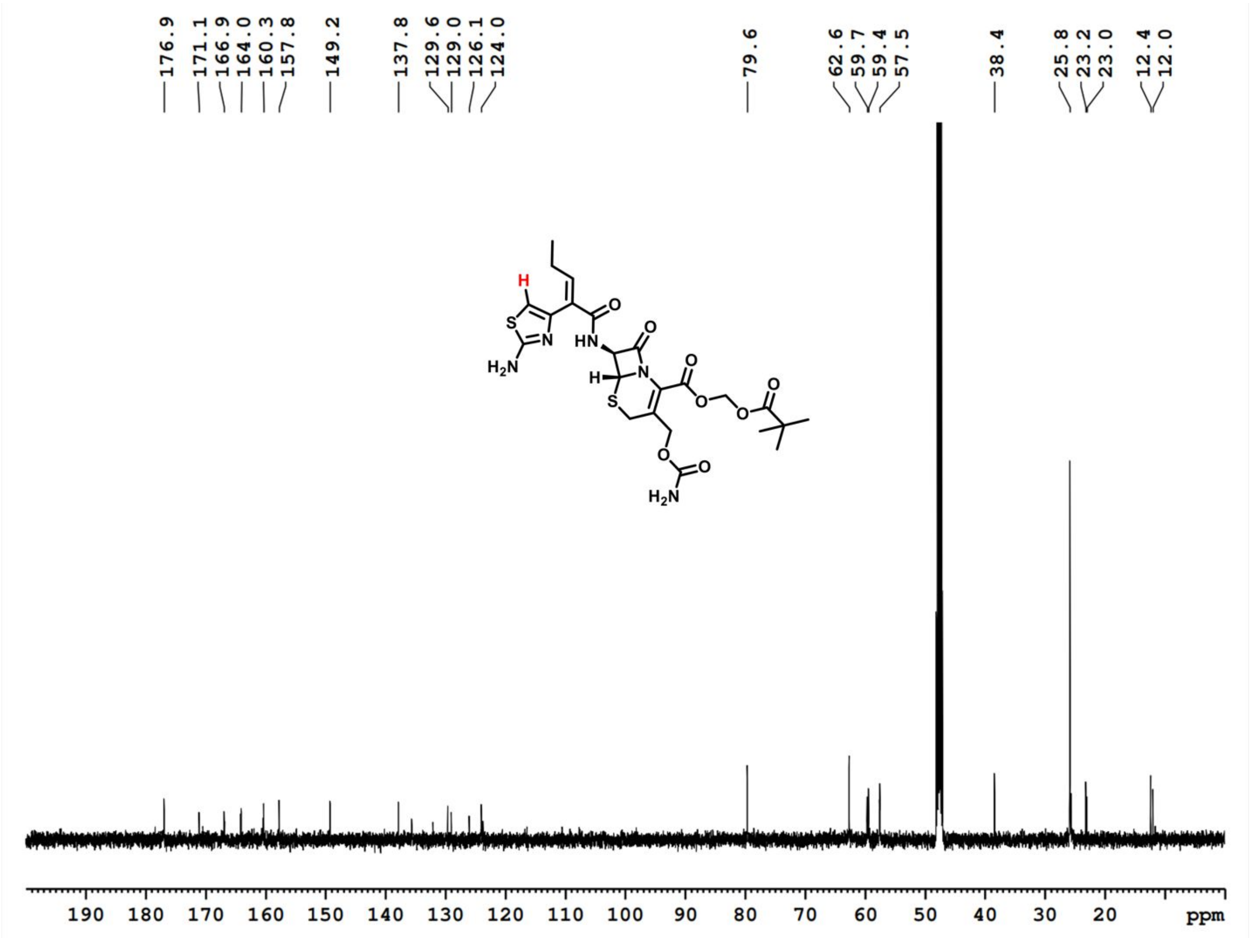

Figure S51: $^{13}C\{^{1}H\}$ NMR spectrum (Methanol-d_4, 125 MHz) of **12**.

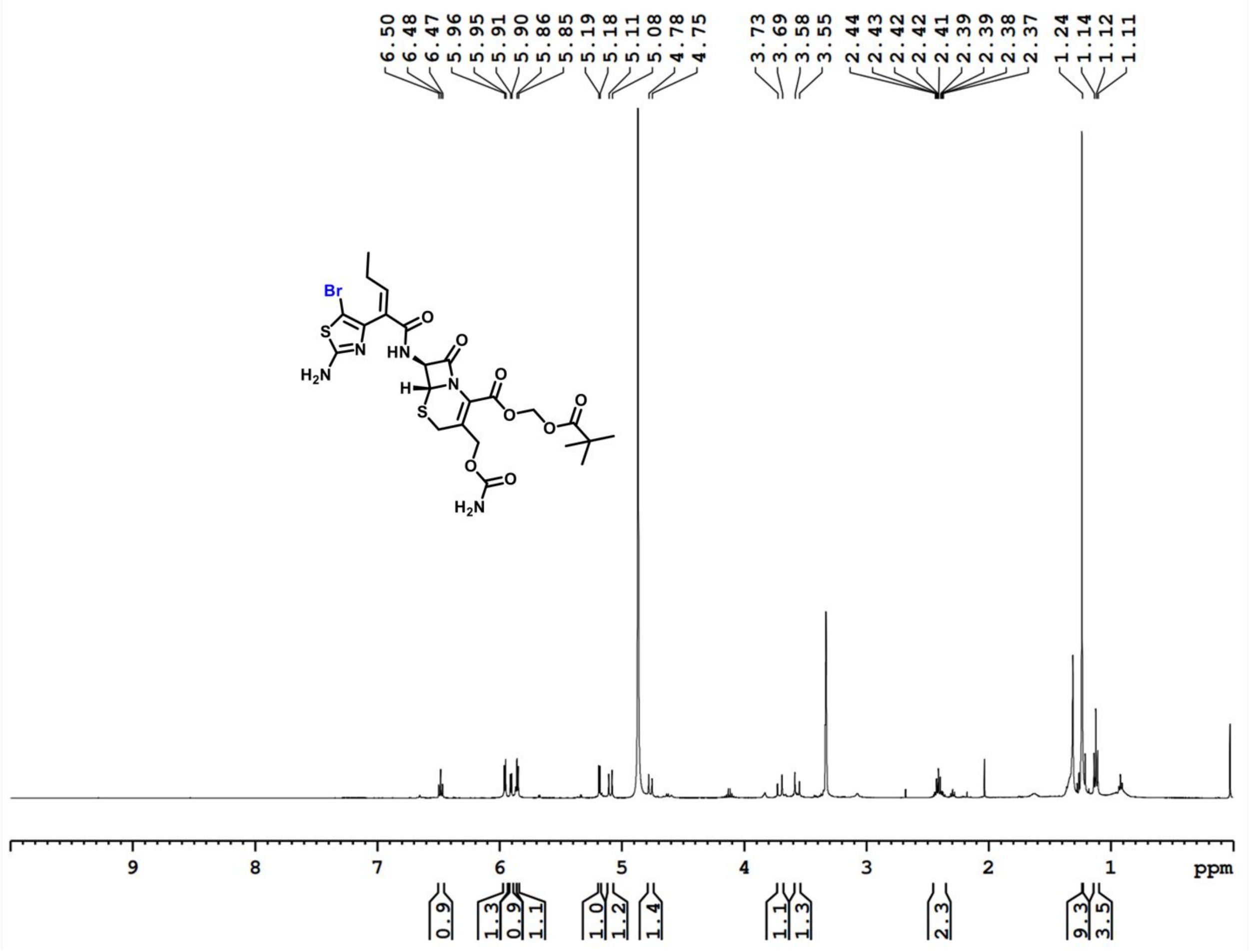

Figure S52: ^{1}H-NMR spectrum (Methanol-d_4, 500 MHz) of **12-Br**.

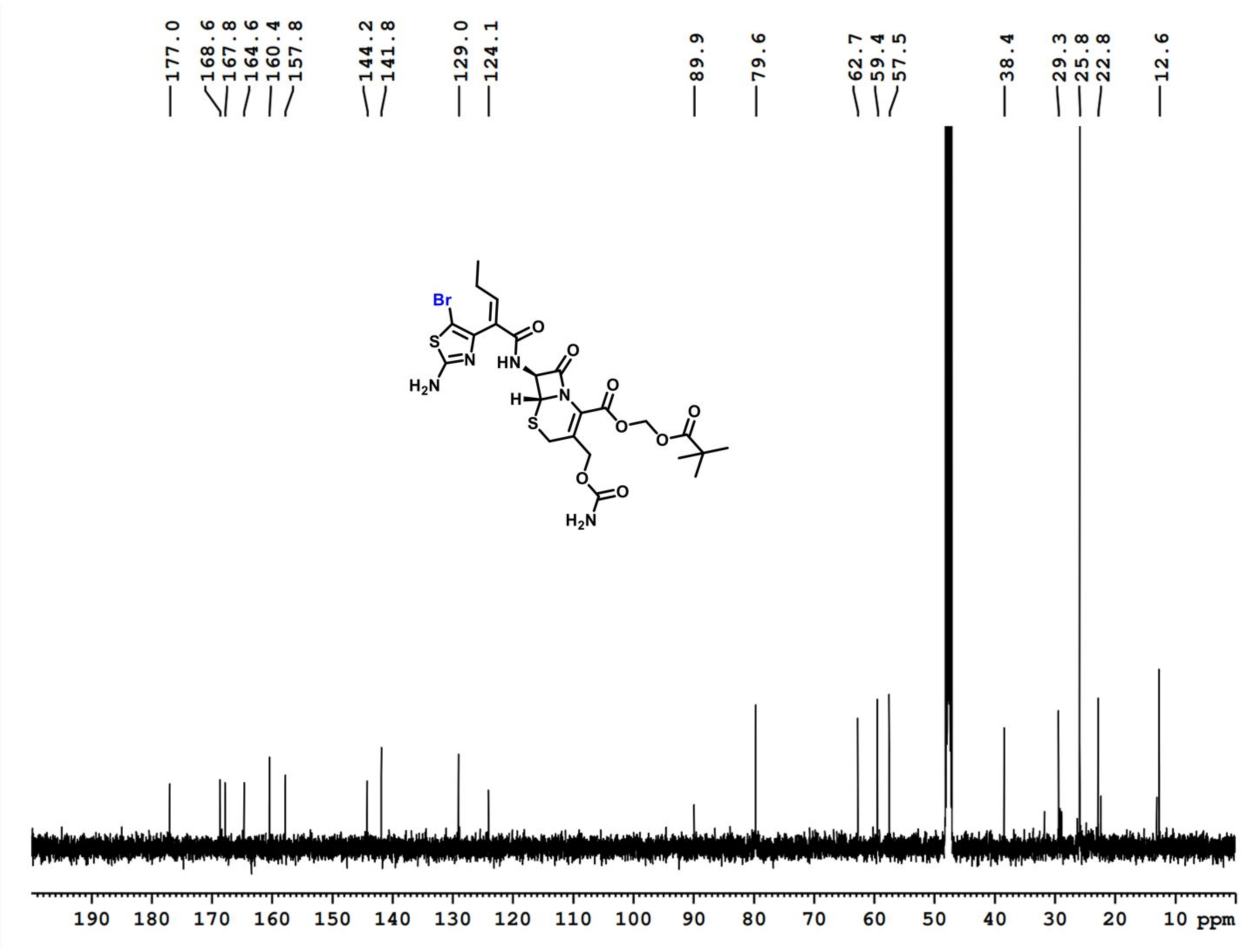

Figure S53: $^{13}C\{^{1}H\}$ NMR spectrum (Methanol-d_4, 125 MHz) of **12-Br**.

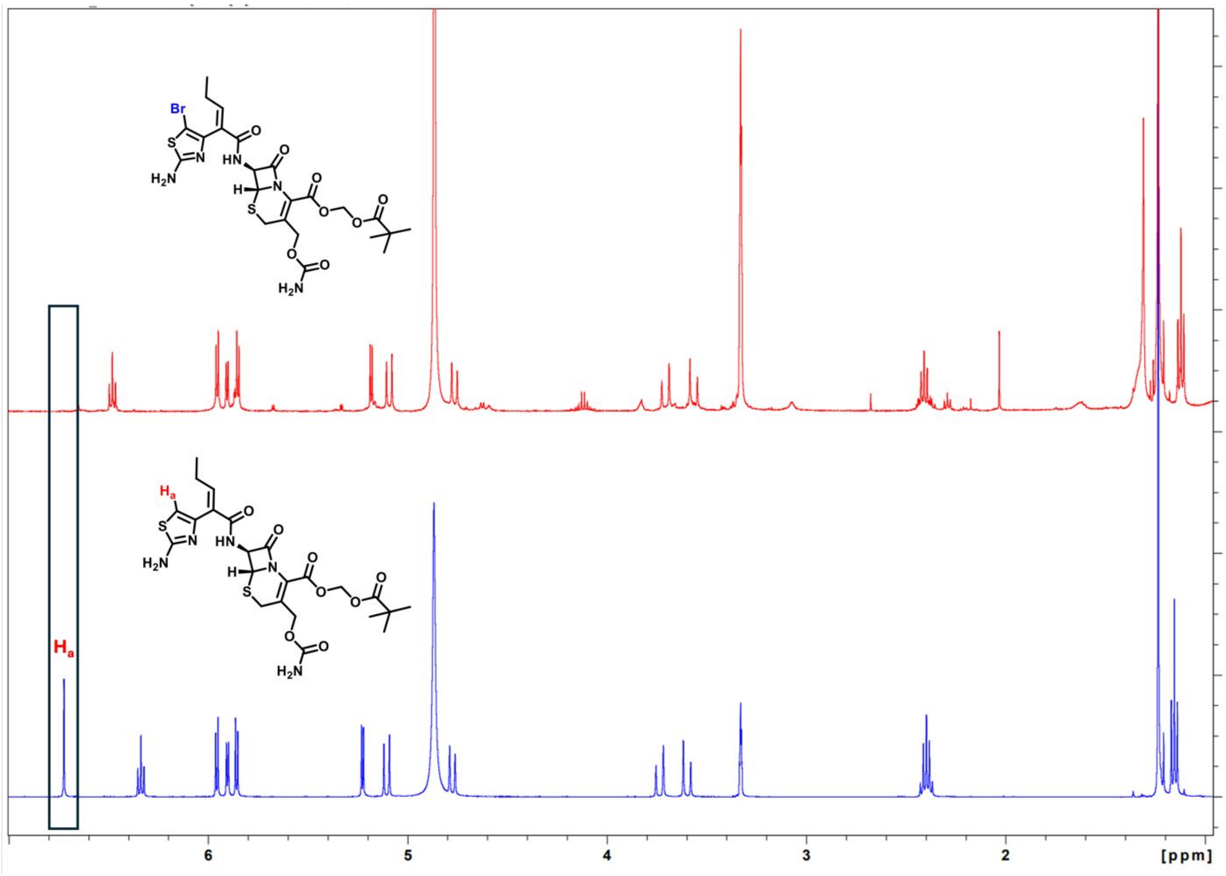

Figure S54: Overlaid ^{1}H-NMR spectra of **12-Br** (top, in red) and **12** (bottom, blue) with the key singlet observed in the aromatic region in the bottom spectra boxed that is missing in the top spectra implying that the aromatic proton of the 2-aminothiazole moiety of **12** was replaced by the bromine atom in **12-Br**.

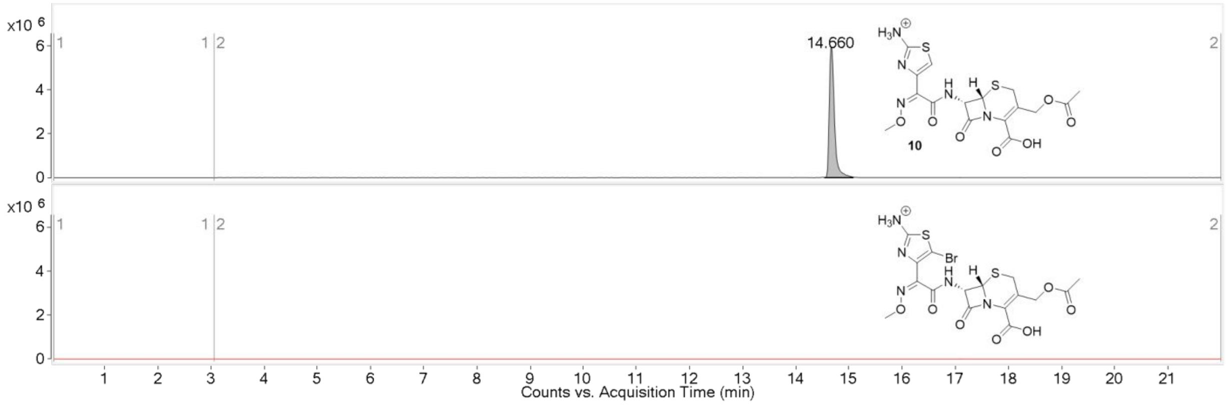

Figure S55: [M+H]$^{1+}$ EICs demonstrating presence of substrate **10** (top panel) and monobrominated product (bottom panel) detected to be produced in a negative control enzymatic reaction wherein the catalyst CcVHPO1 was omitted from the reaction.

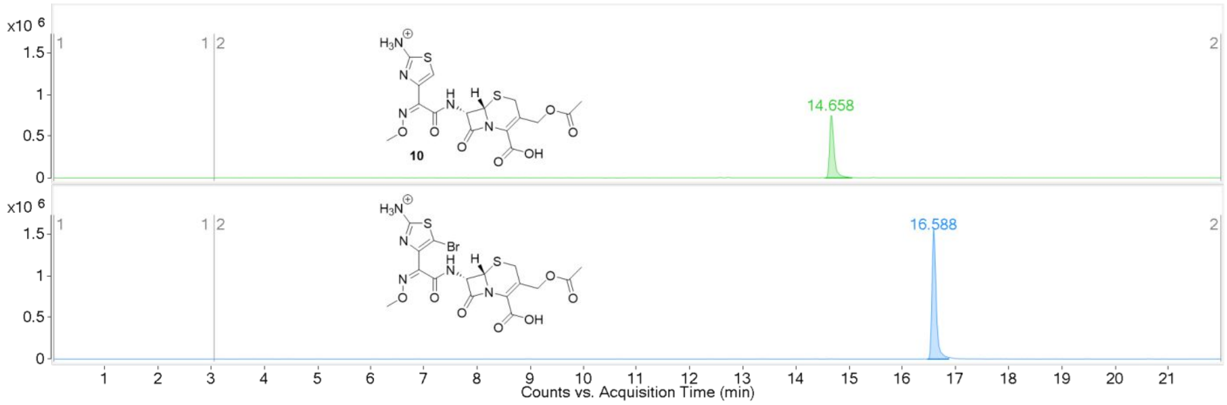

Figure S56: [M+H]$^{1+}$ EICs demonstrating presence of substrate **10** (top panel) and monobrominated product (bottom panel) detected to be produced in an enzymatic reaction using CcVHPO1.

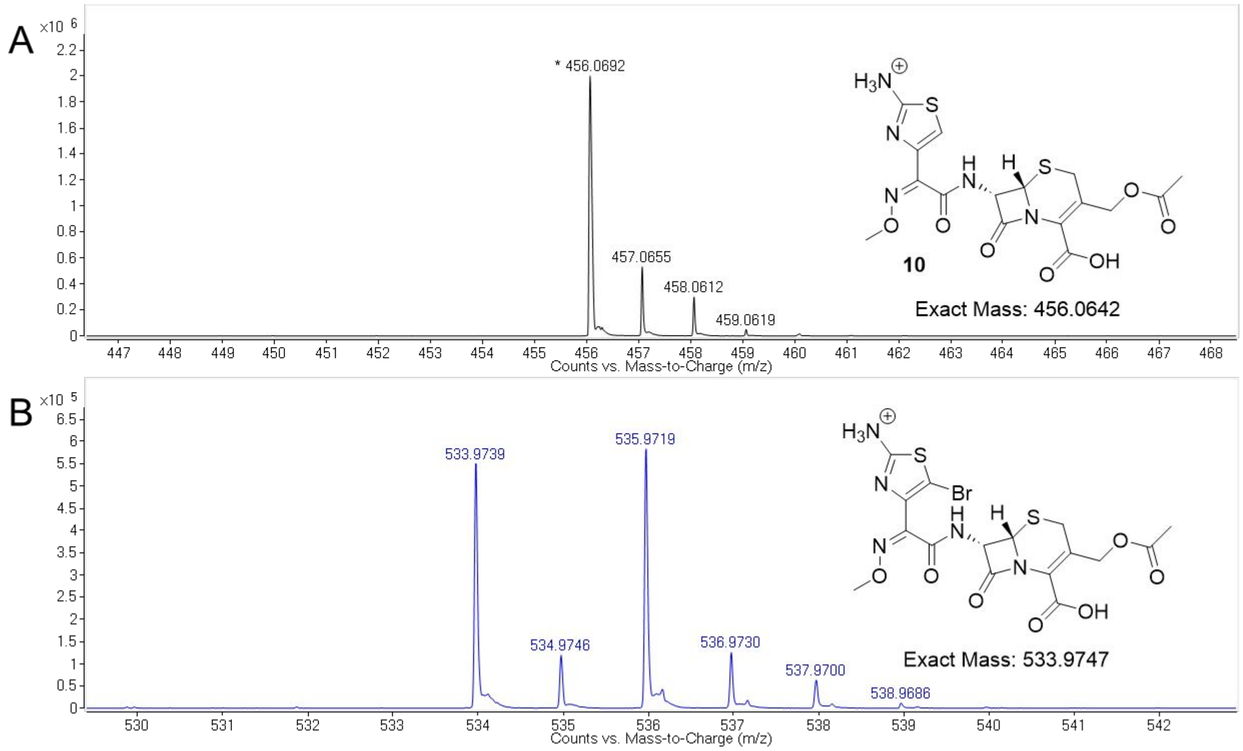

Figure S57: (**A**) $[M+H]^{1+}$ MS^1 spectra of **10**. (**B**) $[M+H]^{1+}$ MS^1 spectra for monobrominated **10**.

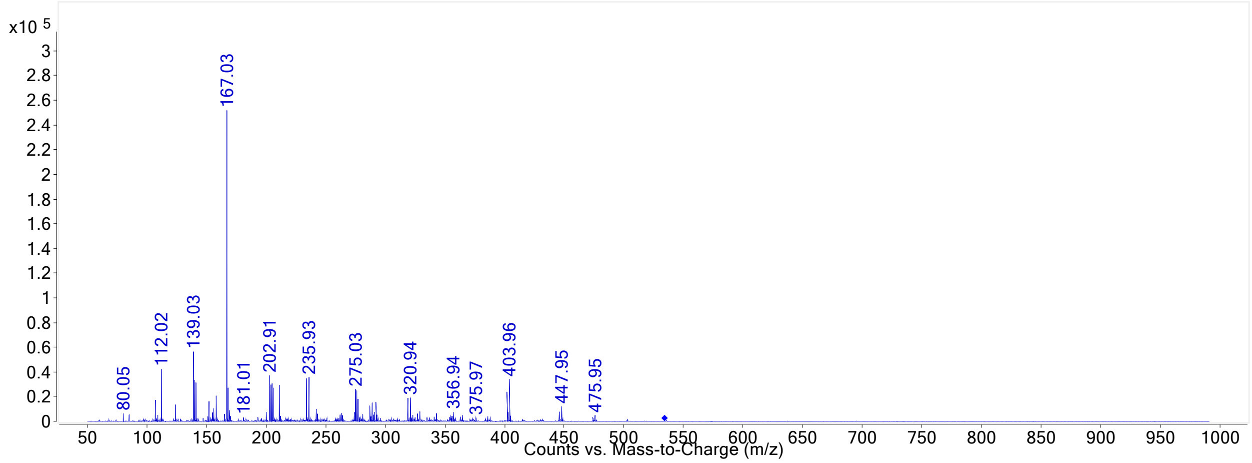

Figure S58: MS^2 fragmentation spectra for mono brominated **10**.

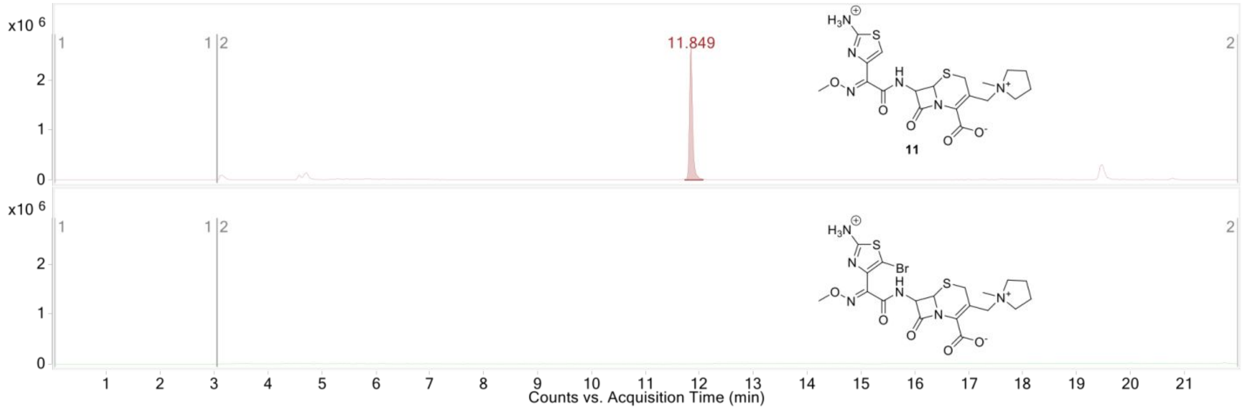

Figure S59: [M+H]$^{1+}$ EICs demonstrating presence of substrate **11** (top panel) and monobrominated product (bottom panel) detected to be produced in a negative control enzymatic reaction wherein the catalyst CcVHPO1 was omitted from the reaction.

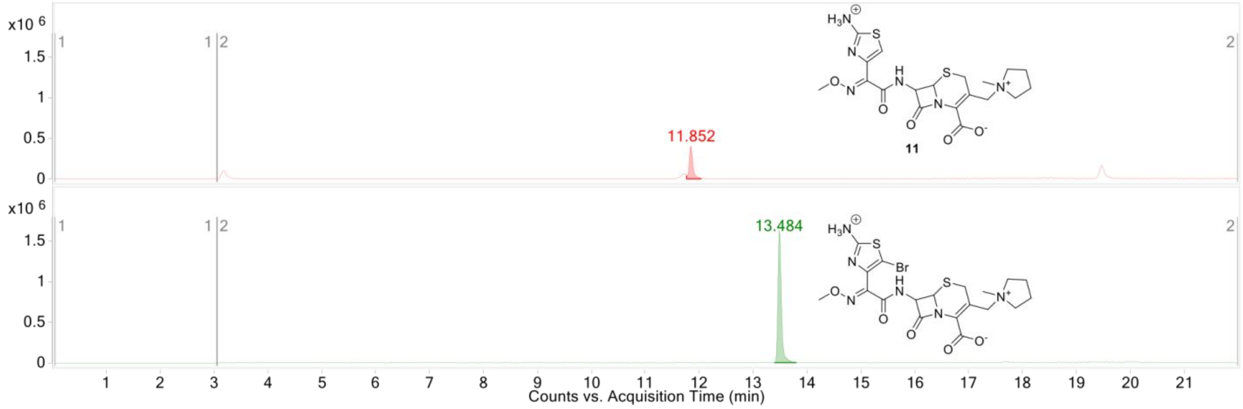

Figure S60: [M+H]$^{1+}$ EICs demonstrating presence of substrate **11** (top panel) and monobrominated product (bottom panel) detected to be produced in an enzymatic reaction using CcVHPO1.

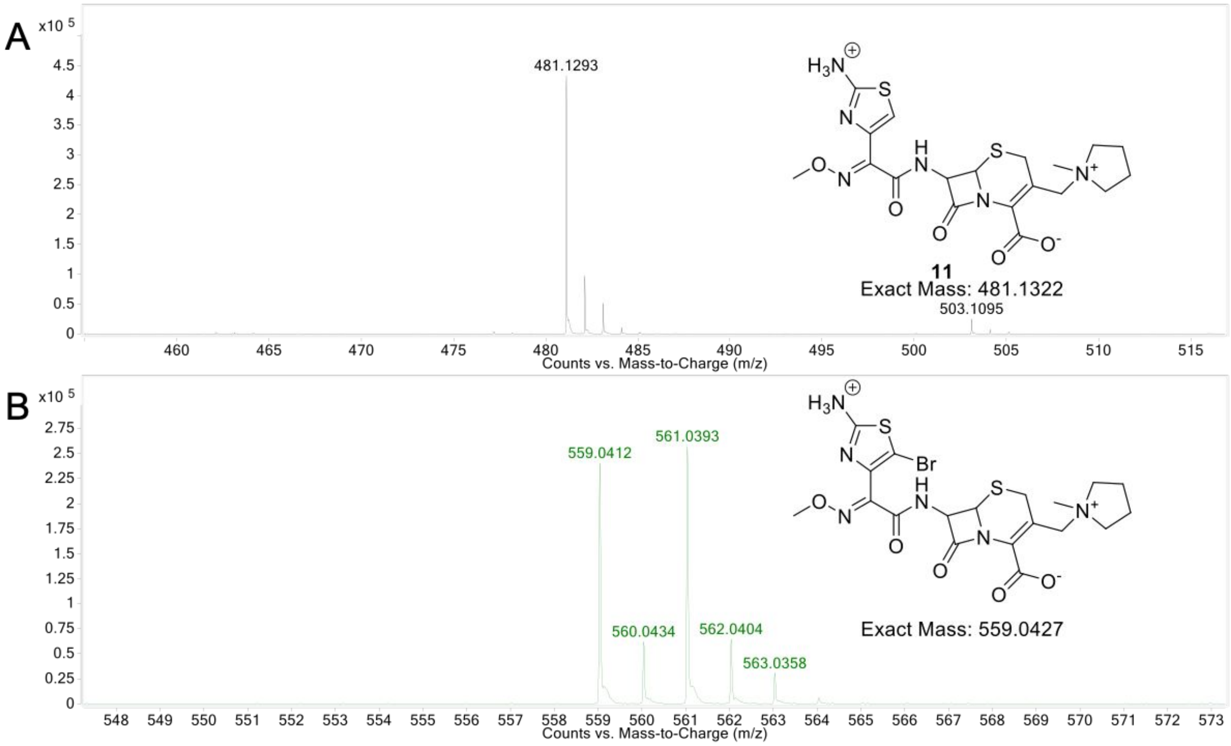

Figure S61: (**A**) $[M+H]^{1+}$ MS^1 spectra of **11**. (**B**) $[M+H]^{1+}$ MS^1 spectra for monobrominated **11**.

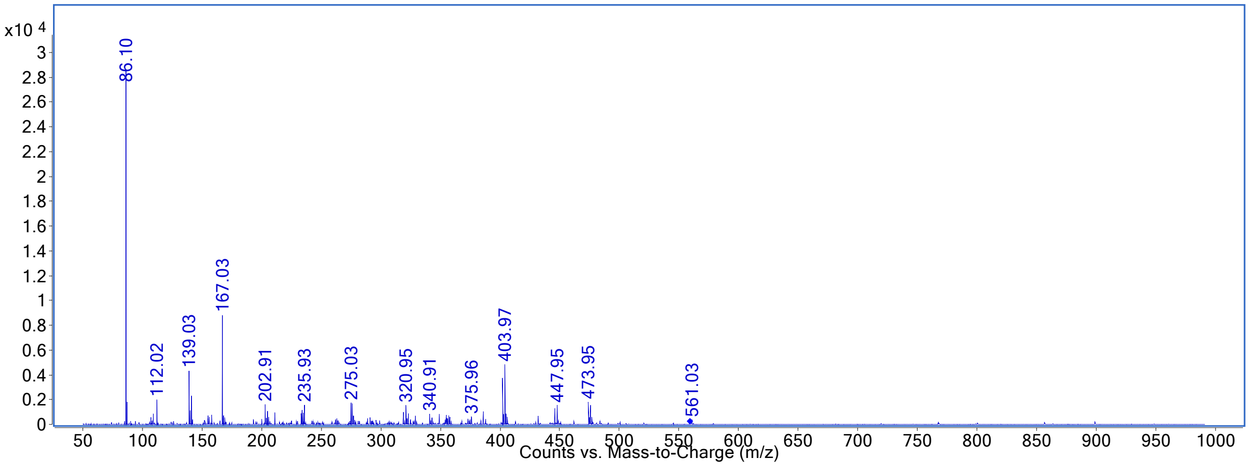

Figure 62: MS^2 fragmentation spectra for mono brominated **11**.

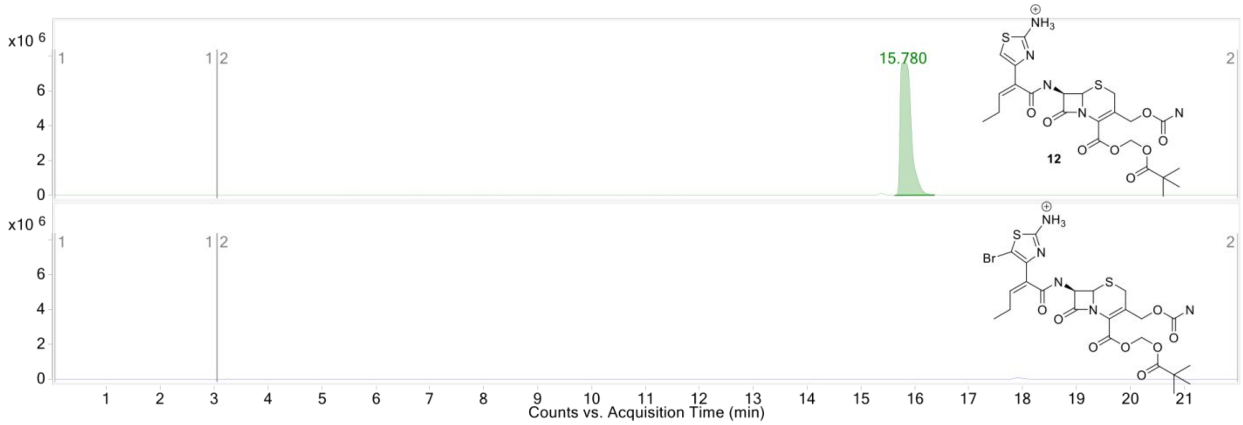

Figure S63: [M+H]$^{1+}$ EICs demonstrating presence of substrate **12** (top panel) and monobrominated product (bottom panel) detected to be produced in a negative control enzymatic reaction wherein the catalyst CcVHPO1 was omitted from the reaction.

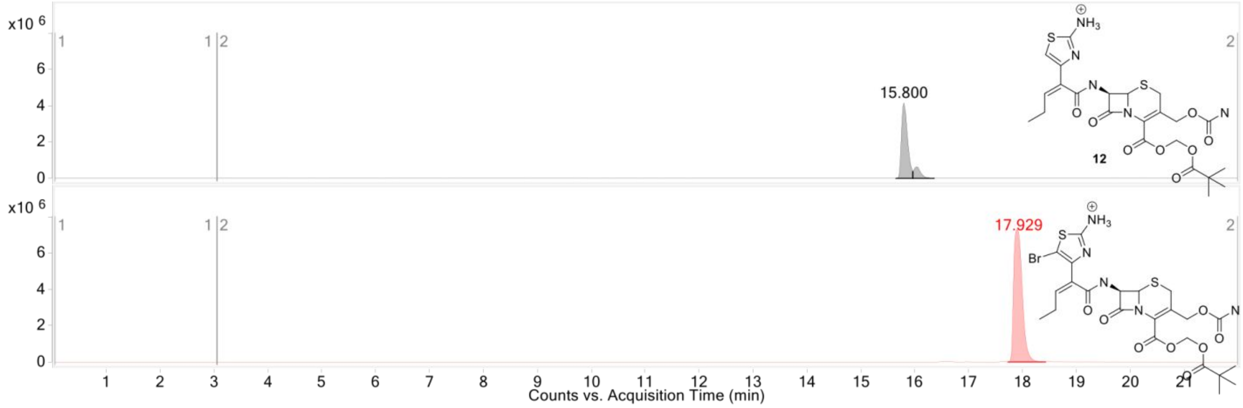

Figure S64: [M+H]$^{1+}$ EICs demonstrating presence of substrate **12** (top panel) and monobrominated product (bottom panel) detected to be produced in an enzymatic reaction using CcVHPO1.

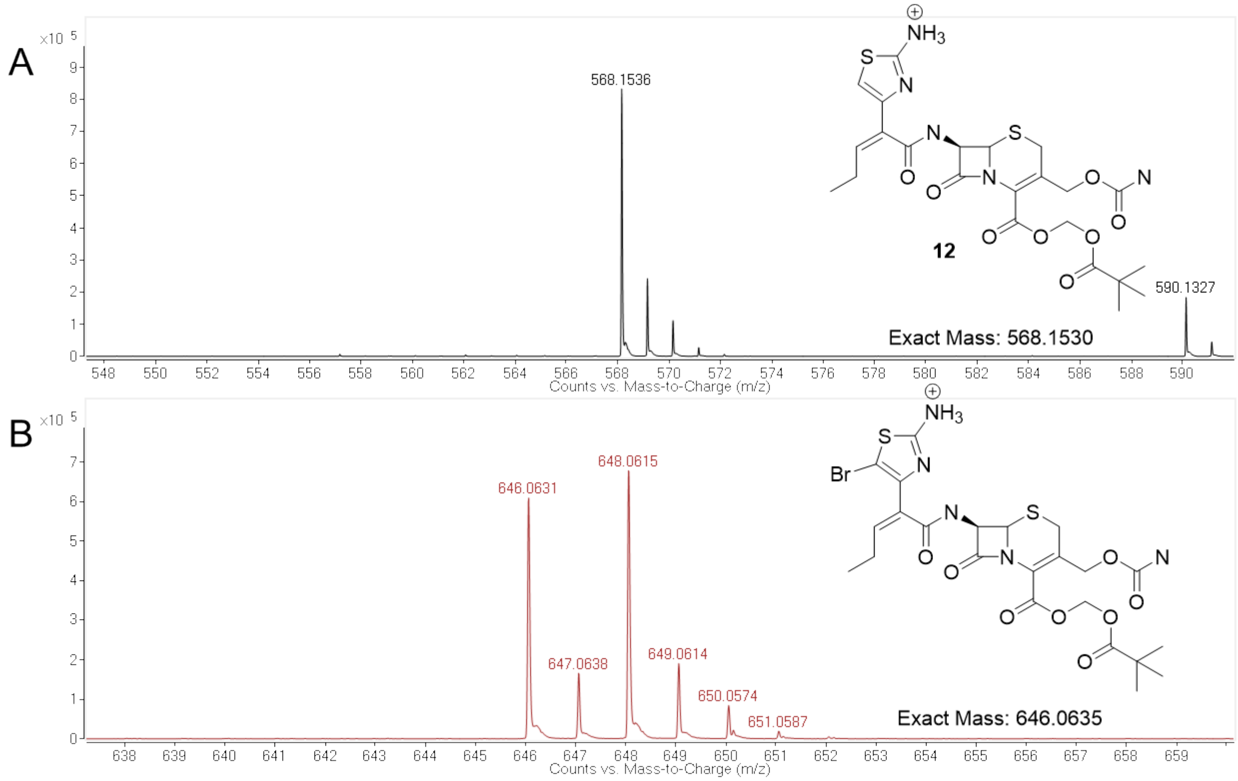

Figure S65: (**A**) [M+H]$^{1+}$ MS^1 spectra of **12**. (**B**) [M+H]$^{1+}$ MS^1 spectra for monobrominated **12**.

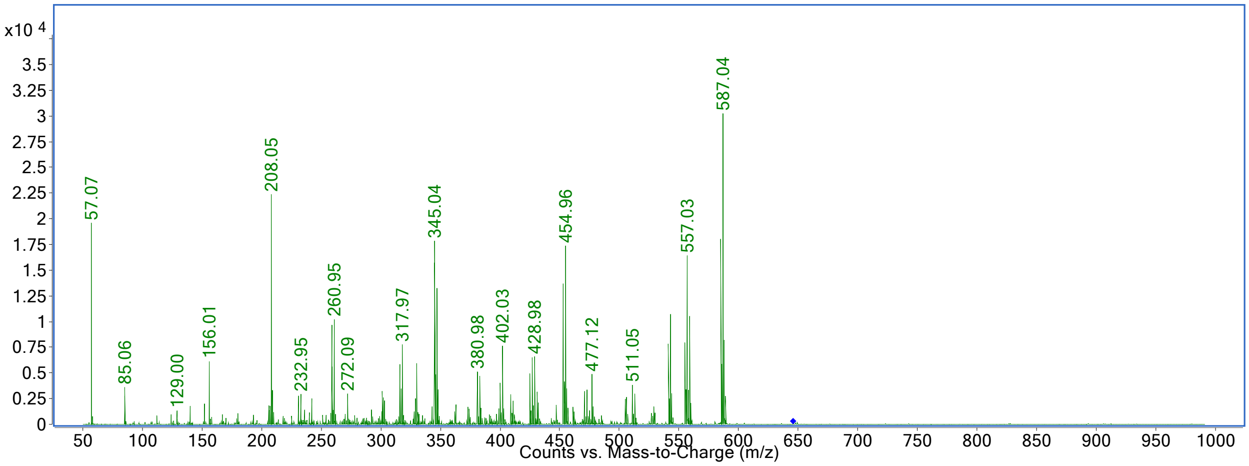

Figure S66: MS^2 fragmentation spectra for monobrominated **12**.

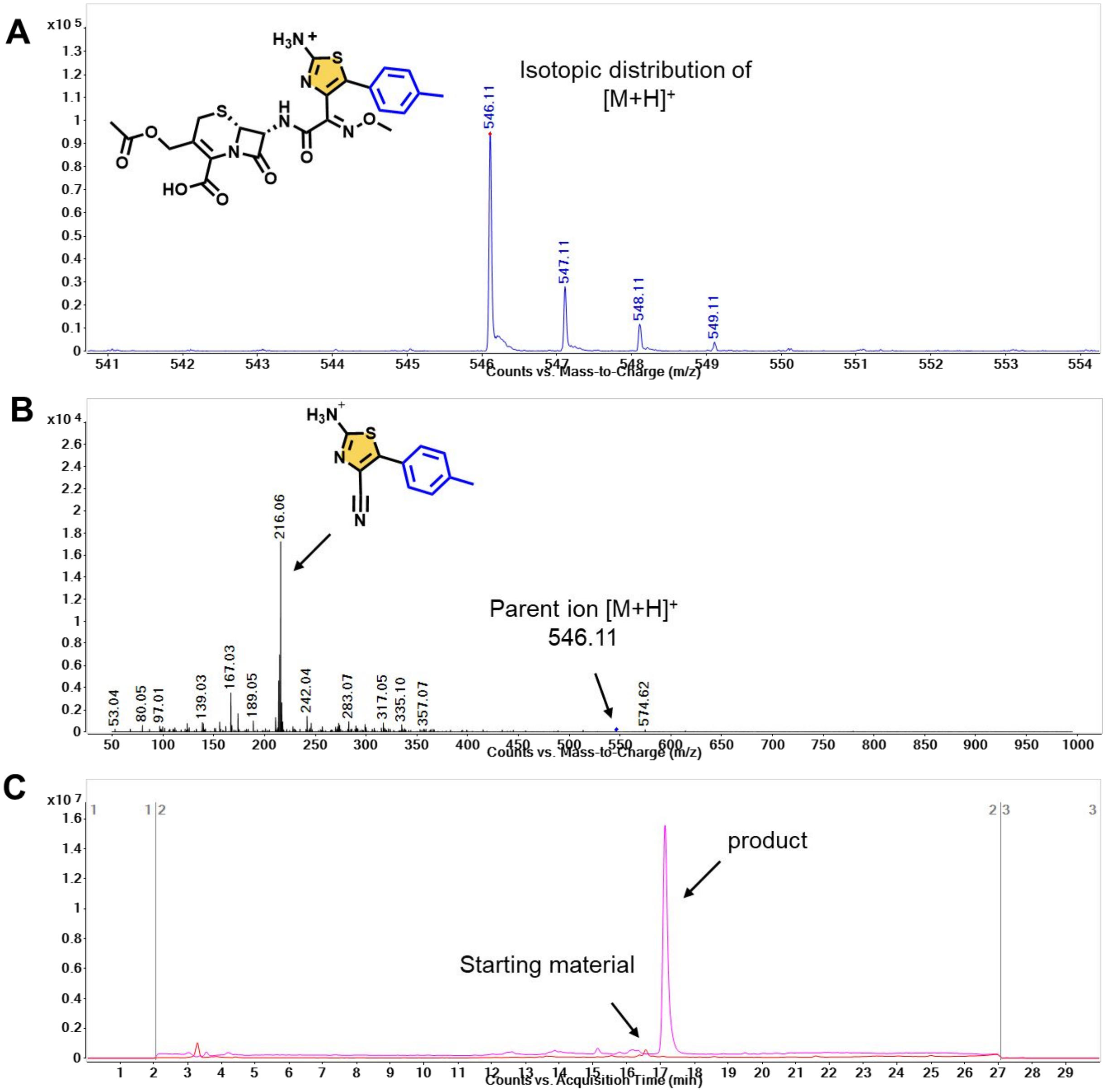

Figure S67: (A) Isotopic distribution of the SMCC product wherein a tolyl group has been added to molecule **10**. (**B**) MS^2 spectra for the SMCC product with a key product ion structurally annotated. **(C)** EICs monitoring product formation and unconverted substrate in the SMCC reaction in pink and red, respectively. The EICs were generated using the most abundant $[M+H]^+$ ions.

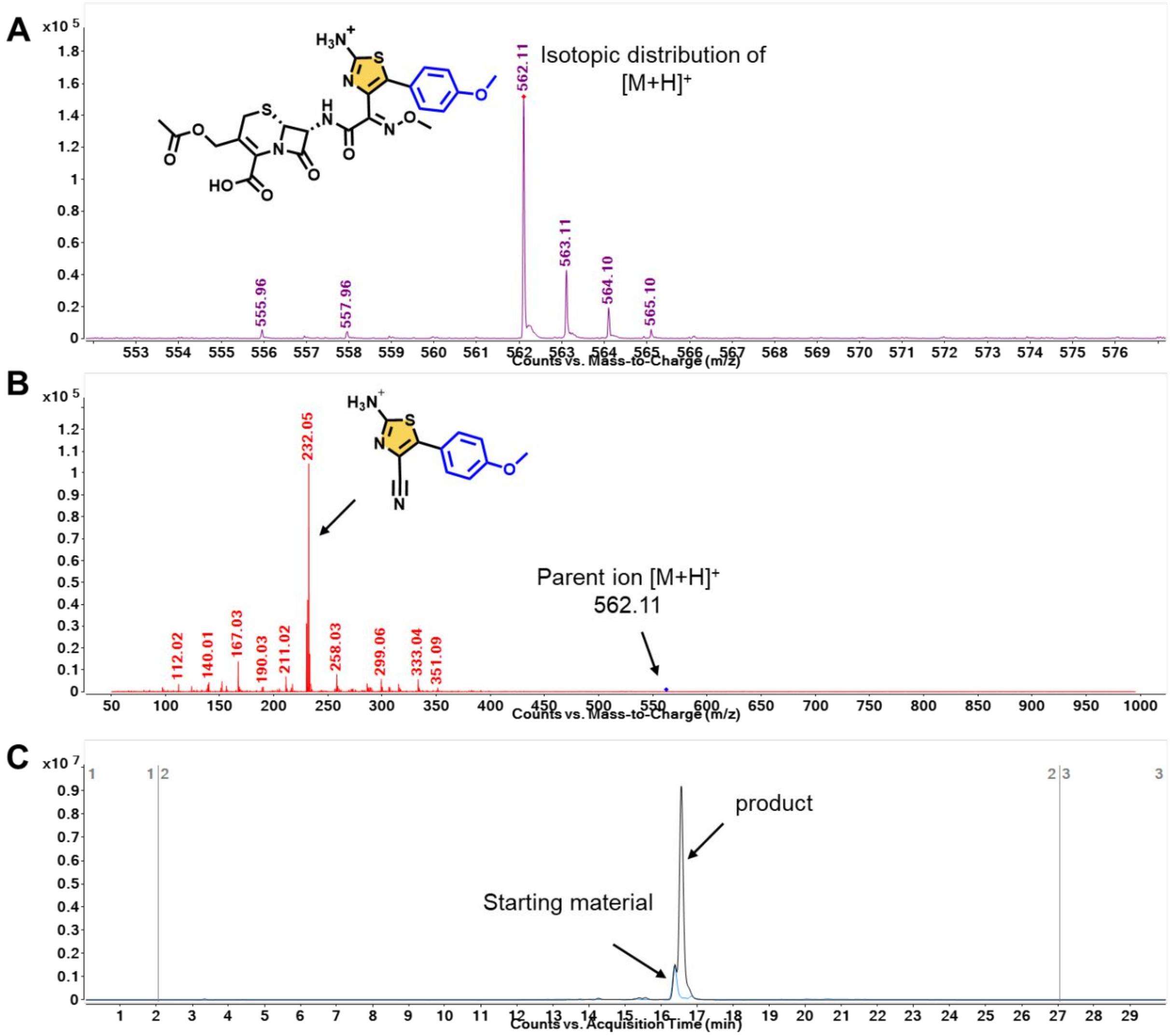

Figure S68: (A) Isotopic distribution of the SMCC product wherein a *p*-methoxyphenyl group has been added to molecule **10**. (**B**) MS^2 spectra for the SMCC product with a key product ion structurally annotated. **(C)** EICs monitoring product formation and unconverted substrate in the SMCC reaction in black and cyan, respectively. The EICs were generated using the most abundant $[M+H]^+$ ions.

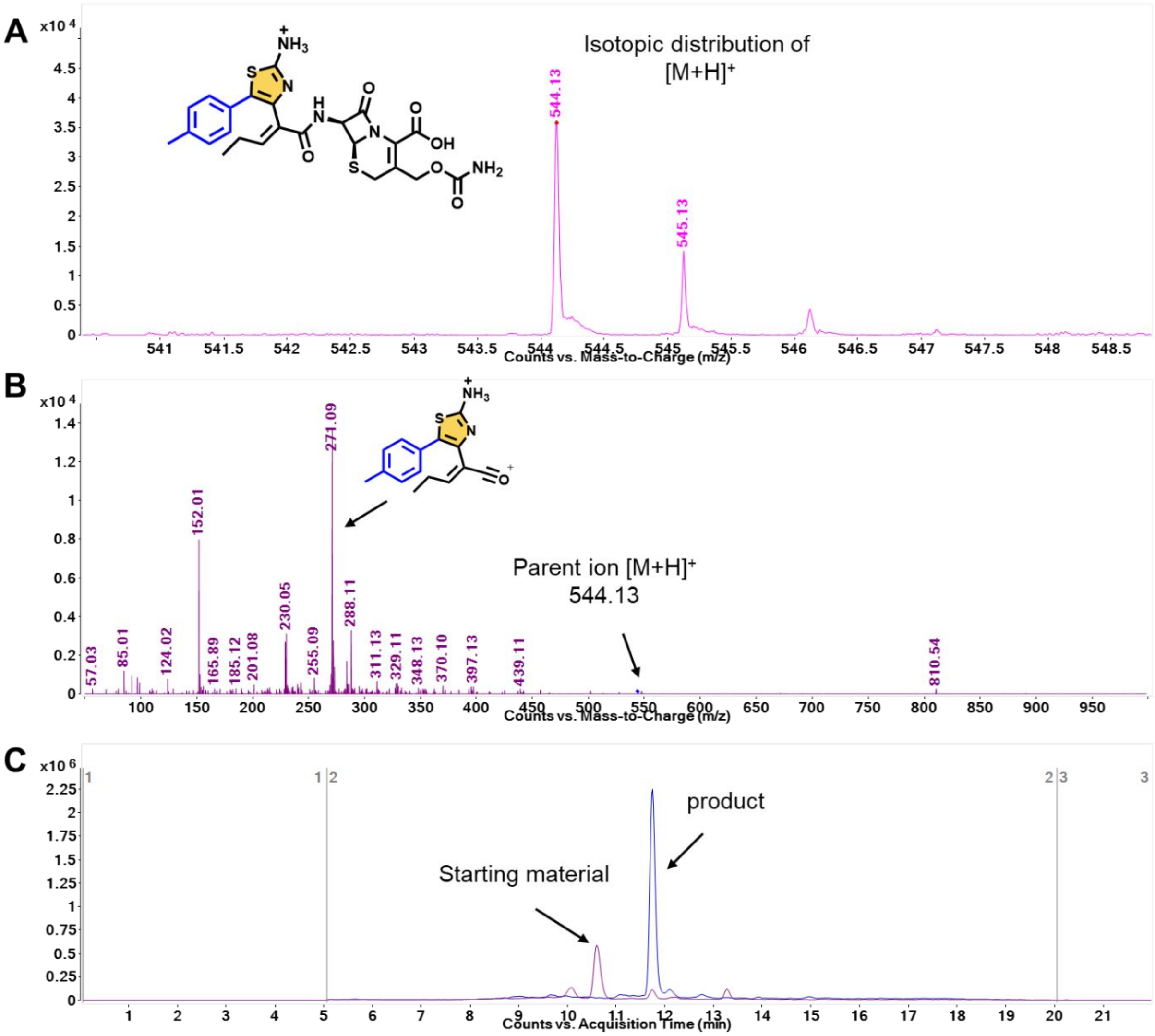

Figure S69: (A) Isotopic distribution of the SMCC product wherein a tolyl group has been added to molecule **12**. (**B**) MS^2 spectra for the SMCC product with a key product ion structurally annotated. **(C)** EICs monitoring product formation and unconverted substrate in the SMCC reaction in blue and purple, respectively. The EICs were generated using the most abundant $[M+H]^+$ ions.

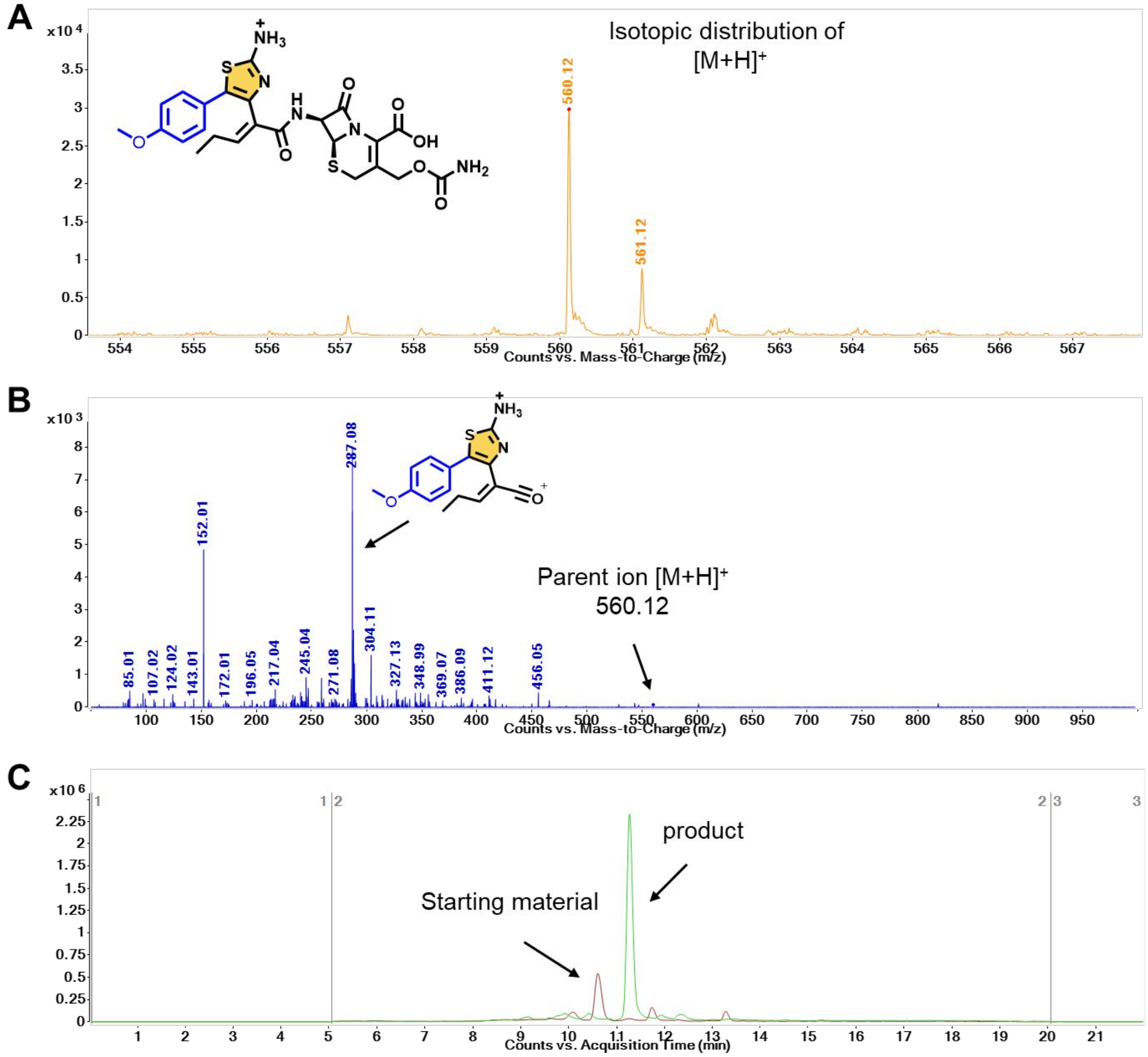

Figure S70: (A) Isotopic distribution of the SMCC product wherein a *p*-methoxyphenyl group has been added to molecule **12**. (**B**) MS^2 spectra for the SMCC product with a key product ion structurally annotated. **(C)** EICs monitoring product formation and unconverted substrate in the SMCC reaction in green and brown, respectively. The EICs were generated using the most abundant [M+H]$^+$ ions.

SUPPLEMENTARY REFERENCES

1. Thapa, H. R.; Lin, Z.; Yi, D.; Smith, J. E.; Schmidt, E. W.; Agarwal, V., Genetic and biochemical reconstitution of bromoform biosynthesis in *Asparagopsis* lends insights into seaweed reactive oxygen species enzymology. *ACS Chemical Biology* **2020,** *15*, 1662-1670.

2. Škedelj, V.; Perdih, A.; Brvar, M.; Kroflič, A.; Dubbée, V.; Savage, V.; O'Neill, A. J.; Solmajer, T.; Bešter-Rogač, M.; Blanot, D.; Hugonnet, J.-E.; Magnet, S.; Arthur, M.; Mainardi, J.-L.; Stojan, J.; Zega, A., Discovery of the first inhibitors of bacterial enzyme d-aspartate ligase from Enterococcus faecium (Aslfm). *European Journal of Medicinal Chemistry* **2013,** *67*, 208-220.

3. Zhao, R.; Gove, S.; Sundeen, J. E.; Chen, B.-C., A new facile synthesis of 2-aminothiazole-5-carboxylates. *Tetrahedron Letters* **2001,** *42*, 2101-2102.

4. Uzelac, E. J.; Rasmussen, S. C., Synthesis of Brominated Thiazoles via Sequential Bromination–Debromination Methods. *The Journal of Organic Chemistry* **2017,** *82*, 5947-5951.

5. Gao, Z.; Gouverneur, V.; Davis, B. G., Enhanced aqueous Suzuki–Miyaura coupling allows site-specific polypeptide 18F-labeling. *Journal of the American Chemical Society* **2013,** *135*, 13612-13615.

6. Thapa, H. R.; Agarwal, V., Obligate brominating enzymes underlie bromoform production by marine cyanobacteria. *Journal of Phycology* **2021,** *57*, 1131-1139.
